# Regulatory network of miRNA, lncRNA, transcription factor and target immune response genes in bovine mastitis

**DOI:** 10.1038/s41598-021-01280-9

**Published:** 2021-11-09

**Authors:** Ashley R. Tucker, Nicole A. Salazar, Adeola O. Ayoola, Erdoğan Memili, Bolaji N. Thomas, Olanrewaju B. Morenikeji

**Affiliations:** 1 Department of Biomedical Sciences, Rochester Institute of Technology, 153 Lomb Memorial Drive, Rochester, NY 14623 USA; 2 Kunming Institute of Zoology, Chinese Academy of Sciences, Kunming, China; 3 Department of Animal and Dairy Sciences, Mississippi State University, Mississippi State, MS 39762 USA; 4 Division of Biological and Health Sciences, University of Pittsburgh at Bradford, 300 Campus Drive, Bradford, PA 16701 USA

**Keywords:** Computational biology and bioinformatics, Genetics, Immunology, Microbiology, Molecular biology, Diseases

## Abstract

Pre- and post-transcriptional modifications of gene expression are emerging as foci of disease studies, with some studies revealing the importance of non-coding transcripts, like long non-coding RNAs (lncRNAs) and microRNAs (miRNAs). We hypothesize that transcription factors (TFs), lncRNAs and miRNAs modulate immune response in bovine mastitis and could potentially serve as disease biomarkers and/or drug targets. With computational analyses, we identified candidate genes potentially regulated by miRNAs and lncRNAs base pair complementation and thermodynamic stability of binding regions. Remarkably, we found six miRNAs, two being bta-miR-223 and bta-miR-24-3p, to bind to several targets. LncRNAs NONBTAT027932.1 and XR_003029725.1, were identified to target several genes. Functional and pathway analyses revealed lipopolysaccharide-mediated signaling pathway, regulation of chemokine (C-X-C motif) ligand 2 production and regulation of IL-23 production among others. The overarching interactome deserves further in vitro*/*in vivo explication for specific molecular regulatory mechanisms during bovine mastitis immune response and could lay the foundation for development of disease markers and therapeutic intervention.

## Introduction

Bovine mastitis, caused by a variety of organisms, including Staphylococci, Streptococci, and Enterobacteria, is a mammary gland infection posing a great threat to the dairy industry, and is the costliest disease affecting cattle, due to its impact on the quality and quantity of milk production^1,2^. The immune response to pathogens in the mammary gland is highly complex, having to first identify the pathogen, recruit the appropriate immune cells, remove the pathogen, and then terminate the inflammatory response^3^. Recent studies have shown that mammary epithelial cells (MEC) initiate this process by changing the expression of various genes which are responsible for a wide range of immunological and inflammatory responses^4^. The regulatory elements involved in the over- or under-expression of these genes during bovine mastitis are yet to be fully understood and may be a potential area for initiating prevention and control of a global, economically devastating disease in the dairy industry.

Of recent, attention has shifted to regulatory elements, such as miRNAs, lncRNAs, and TFs, possibly influencing the expression of host immune genes^5,6^. MiRNAs are short, non-coding RNA that bind to the 3′ untranslated region (UTR), 5′UTR and coding regions of an mRNA to post-transcriptionally modify genes, repressing their translation into their final protein product^5,7^. Likewise, lncRNAs are long non-coding RNA transcripts, harboring a 5′ cap and 3′ poly (A) tail but no functional open reading frame, and as such cannot encode proteins^8,9^. This heterogeneous class of RNAs can modulate gene expression and protein synthesis at the transcriptional and post-transcriptional levels via complementary base pairing^10^. LncRNAs play key roles in regulating several cellular and developmental processes, including genomic imprinting, DNA methylation, splicing and chromatin modification^11,12^. With an abundance of binding sites for miRNA and mRNA, lncRNA can act as ceRNA (competing endogenous RNA) and are significant regulatory elements in post-transcriptional gene expression^13,14^. For example, lncRNA adipocyte differentiation-associated long noncoding RNA (ADNCR) targets miR-204 significantly regulating Sirtuin 1 (SIRT1) gene expression at the mRNA and protein level, inhibiting adipogenesis in the process^15^. Additionally, Li and colleagues showed that lncRNA H19 targets miRNAs let-7b and miR-200b, competes endogenously with transcription factor Sp1 (SP1), thereby regulating the expression of TGF-β-receptor 2 (TGFBR2) gene in cancers^9^. On the other hand, TFs are proteins capable of altering or activating gene-expression level^16,17^.

Here, we propose the need to understand the crosstalk between miRNA, lncRNA and TFs, and the role they play in regulating genes associated with bovine mastitis, including the possibility of translation to drug treatment or disease prevention therapies. To do this, we identified candidate miRNAs, lncRNAs and TFs, and their potential role in bovine mastitis via in silico analyses. Current algorithms, relying on base pair complementation, evolutionary conservation, and thermodynamic stability of binding regions, have been shown to be useful in predicting miRNA, lncRNA and TF binding sites on target genes^18,19^. Multiple miRNA databases such as miRWalk^20^, miRNet^21^, and TargetScan^22^ compute potential miRNA-mRNA interactions, while the role of individual miRNA can be inferred through functional analysis with Gene Ontology (GO)^23^. Similarly, lncRNA prediction binding software including NONCODE^24^, lncRNA2Target^25^, and lncTar^19^ have been useful in guiding bench experiments thereby saving time and resources. Knowing the interconnectivity of key regulatory elements involved in bovine mastitis is potentially useful in disease detection, especially in subclinical cases. If a regulatory element plays a key role in disease pathogenesis or in the immune response, this may be an ideal drug target for treatment.

## Results

### Dataset of candidate genes associated with immune response to bovine mastitis

**Figure 1:**
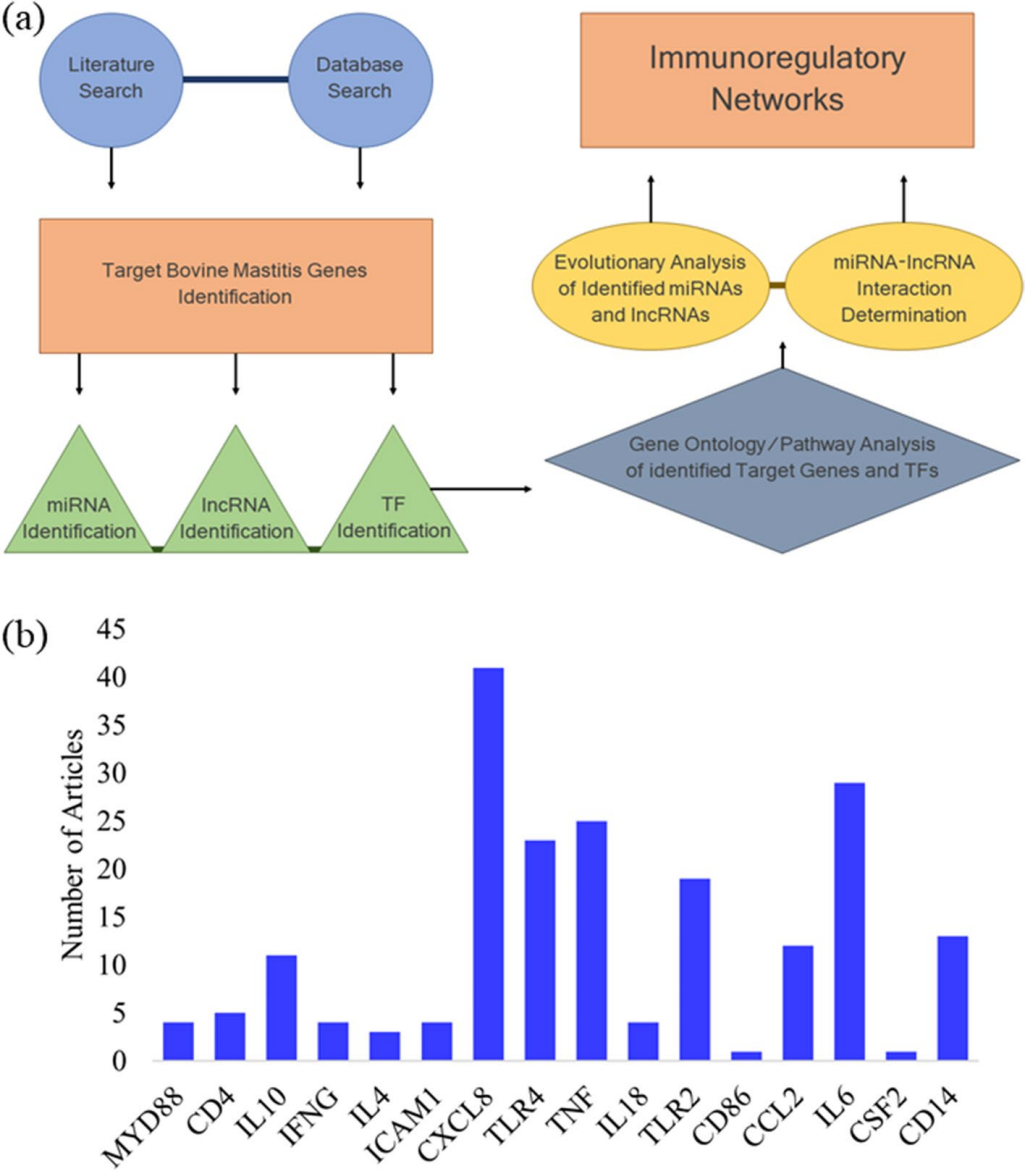
Step-by-step pipeline of analysis procedures (**a**) and number of articles mentioning each of the 16 target bovine mastitis genes (**b**). Figure created with Microsoft PowerPoint (2013) (https://www.microsoft.com/en-us/microsoft-365/powerpoint).

**Figure 2:**
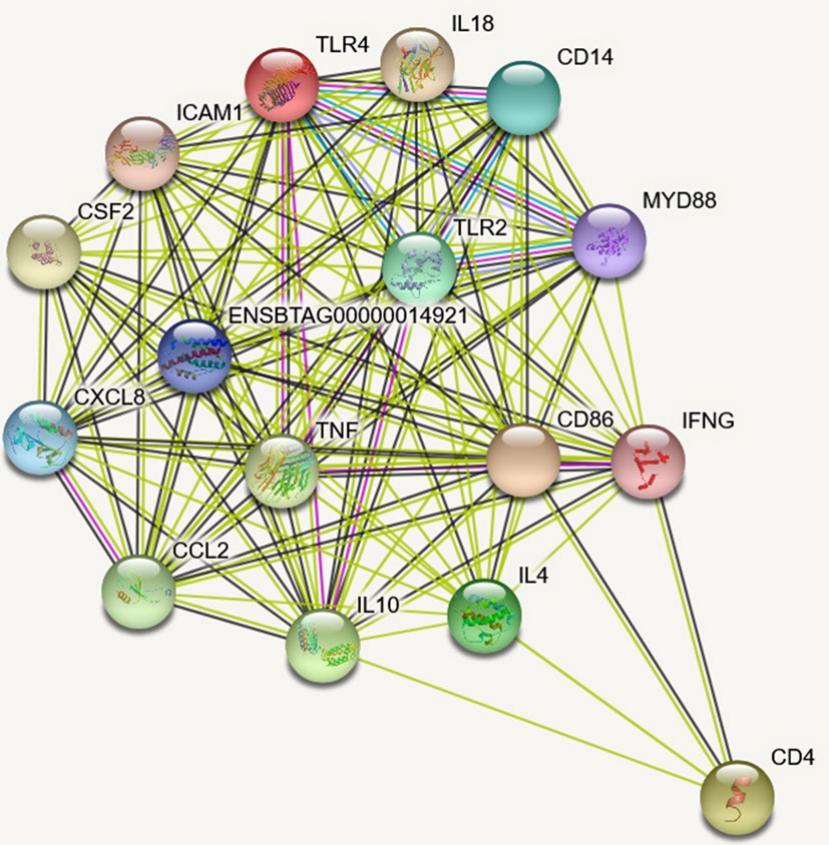
Network of 16 target bovine mastitis genes created using STRING (string-db.org). The Ensembl ID in the network refers to IL-6. Edge color legend (blue: from curated database, pink: experimentally determined, green: text mining, brown: co-expression).

Our initial search on PubMed using the keywords “bovine mastitis” and “gene expression” generated 276 articles. Following filtering, a total of 109 articles were selected and added to the pipeline (Fig. 1a) for candidate genes identification. These articles, including experimental, meta-analysis, and reviews mentioned 919 individual genes associated with host immune response to bovine mastitis. Of importance to our analysis are genes with multiple mentions, including CXCL8 and IL-6 referenced in 41 and 29 articles, respectively (Fig. 1b). Comparing the 919 genes found during our search with the 20 genes generated from Genomatix, yielded 16 genes that were common to both search methods (Fig S1). These genes are MYD88, CD4, IL-10, IFNγ, IL-4, ICAM1, CXCL8, TLR4, TNFα, IL-18, TLR2, CD86, CCL2, IL-6, CSF2, and CD14 (Table 1). The interactions between these candidate genes reveal TLR4 and TLR2 are considerably more linked to other genes (Fig. 2), while CD4 on the other hand had the least interaction.

**Table 1:** List of candidate bovine mastitis genes and their accession number, genomic location, and strand type.

Candidate gene	Accession number	Genomic location	Strand (+/−)
MYD88	NM_001014382	22:11625515–11629955	+
CD4	NM_001103225	5:103631360–103654878	–
IL-10	NM_174088	16:4550836–4555318	–
IFNγ	NM_174086	5:45624462–45629336	+
IL-4	NM_173921	7:21696248–21704136	−
ICAM1	NM_174348	7:14787173–14797855	+
CXCL8	NM_173925	6:88810418–88814572	+
TLR4	NM_174198	8:107057826–107068836	+
TNFα	NM_173966	23:27716168–27719047	−
IL-18	NM_174091	15:22475988–22502331	−
TLR2	NM_174197	17:3954019–3967242	−
CD86	NM_001038017	1:66543642–66610926	+
CCL2	NM_174006	19:15902777–15905368	−
IL-6	NM_173923	4:31454749–31459131	+
CSF2	NM_174027	7:22398938–22401303	−
CD14	NM_174008	7:51762895–51765768	−

### miRNAs bind to the candidate genes in bovine mastitis

**Figure 3:**
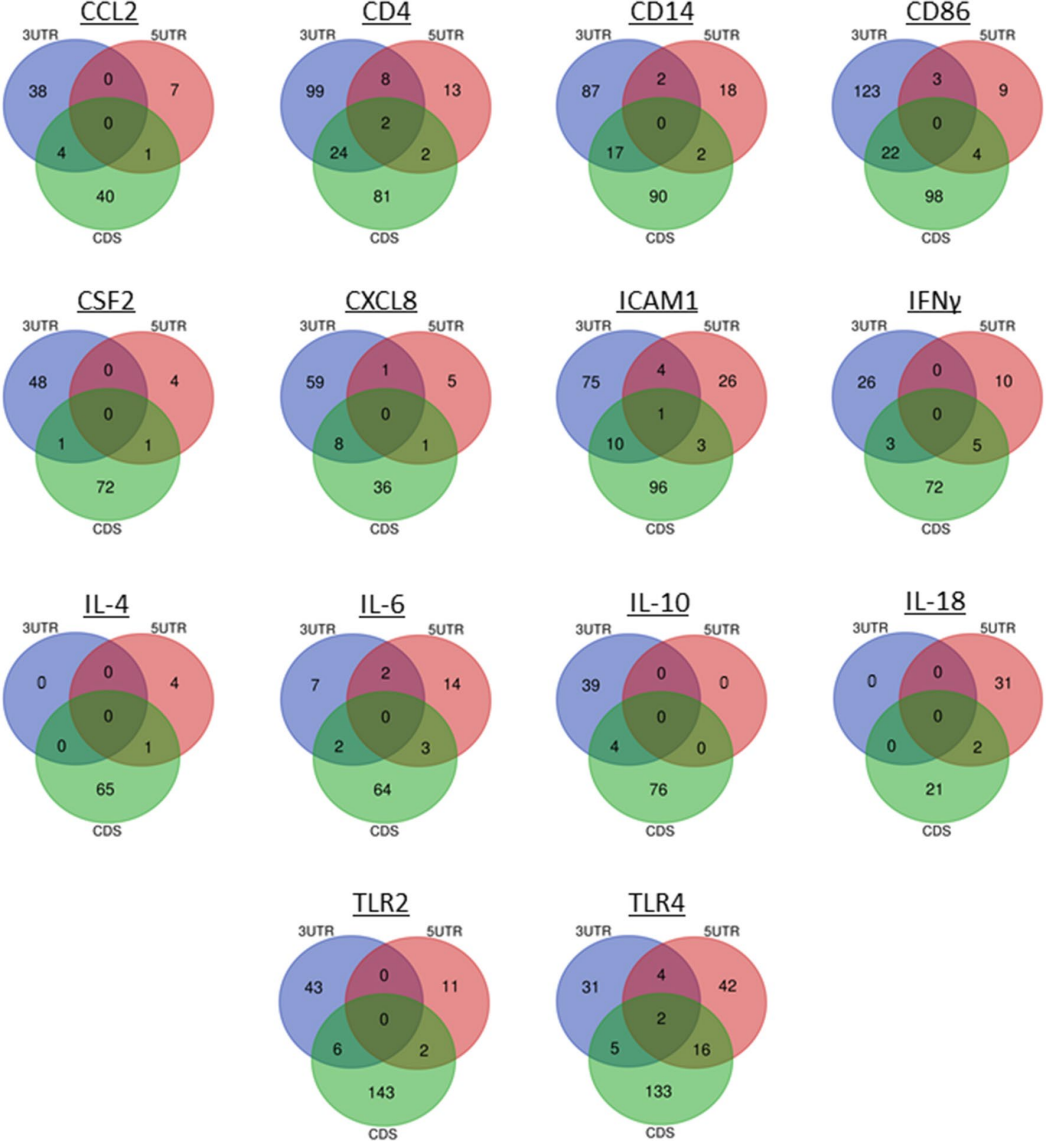
Venn Diagrams (http://bioinformatics.psb.ugent.be/webtools/Venn/) for 14 out of 16 target bovine mastitis genes showing the number of miRNA binding to the 3′ UTR, CDS, and 5′ UTR. The overlapping region of each diagram represents miRNA that bind to all three regions of the target gene.

**Figure 4:**
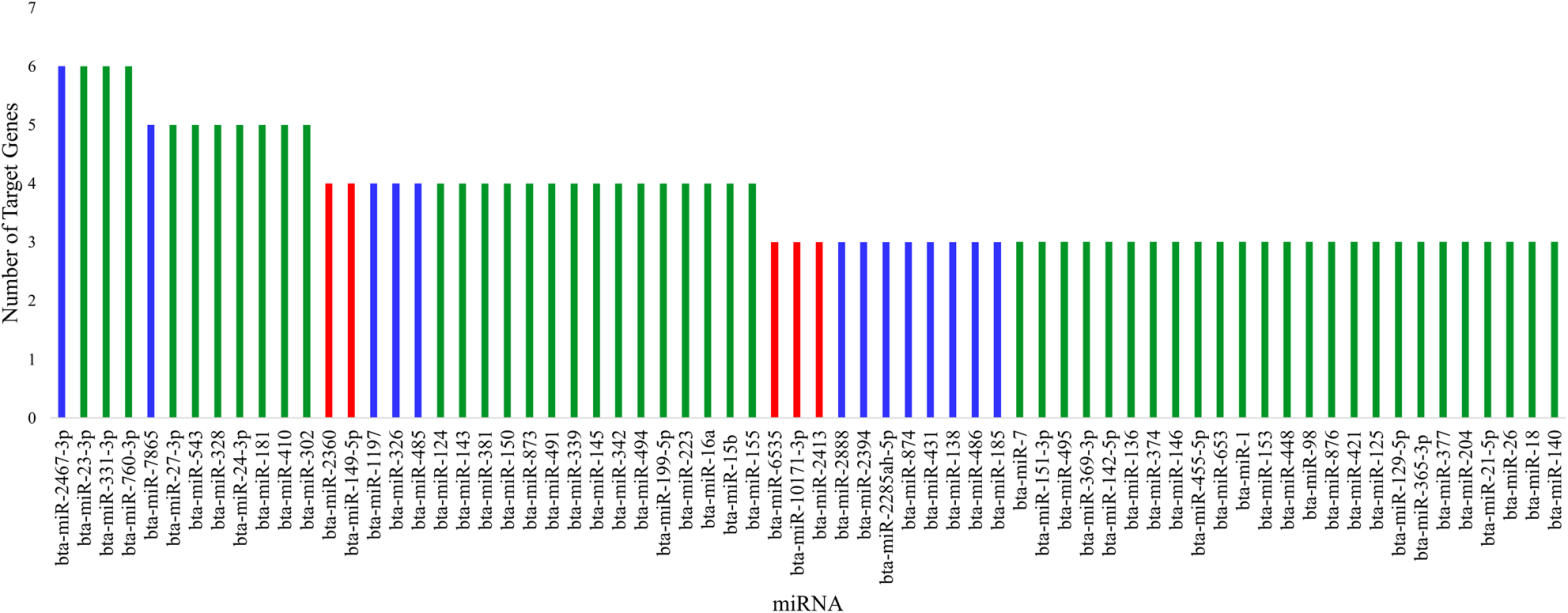
miRNA predicted to bind to three or more target genes. The red bars are miRNA predicted to bind to 3′ UTR, CDS, and 5′ UTR. The blue bars represent miRNA binding to two of the three regions. The green bars are miRNA predicted to bind to one of the three regions. Figure created with Microsoft Excel (2013) (https://www.microsoft.com/en-us/microsoft-365/excel).

Our analyses explored the complementary binding of miRNAs to the 3′UTR, CDS, and 5’UTR regions of the candidate genes. We report a total of 768 miRNAs binding either the 3′ UTR, CDS, or 5′ UTR of the 16 target genes (Table S2). With Bioinformatics and Evolutionary Genomics (BEG), five miRNAs were uncovered to bind all three regions within the same gene; bta-miR-2338 and bta-miR-2433 to CD14, bta-miR-2373-3p to ICAM1, bta-miR-2328-3p and bta-miR-2356 to TLR4 (Fig. 3). In addition, a substantial number of miRNAs bound to two or more regions within the same gene (Fig. 3), Displaying all the miRNA predicted to bind to three or more target genes is Fig. 4, which also depicts whether the miRNA binds one (green), two (blue), or three (red) of the possible regions (3′UTR, 5′UTR, and CDS). The complete list of miRNAs binding three or more target genes is depicted in Table S3. Likewise, we found six miRNAs; bta-miR-24-3p, bta-miR-149-5p, bta-miR-223, bta-miR-185, bta-miR-874, and bta-miR-328, commonly predicted by the three software as shown (Fig S2; Table 2).

**Table 2:** Significant miRNA predicted by all three software and their mRNA sequence, accession number, genomic location, strand type and length.

miRNA	mRNA sequence	Accession	Genomic location	Strand (+ /−)	Length (bp)
bta-miR-149-5p	UCUGGCUCCGUGUCUUCACUCCC	MI0021115	3:120921662–120921751	+	23
bta-miR-185	UGGAGAGAAAGGCAGUUCCUGA	MI0009758	17: 75173467–75173545	+	22
bta-miR-223	UGUCAGUUUGUCAAAUACCCCA	MI0009782	X: 94562822–94562929	−	22
bta-miR-24-3p	UGGCUCAGUUCAGCAGGAACAG	MIMAT0003840			22
bta-miR-328	CUGGCCCUCUCUGCCCUUCCGU	MI0009800	18: 35062240–35062331	−	22
bta-miR-874	CUGCCCUGGCCCGAGGGACCGA	MI0009900	7: 50886238–50886313	−	22

These miRNAs are from the Venn diagram generated through the overlap of the three softwares-miRWalk, miRNet an TargetScan.

### lncRNAs interact with mRNA target inferring their functionalities

**Figure 5:**
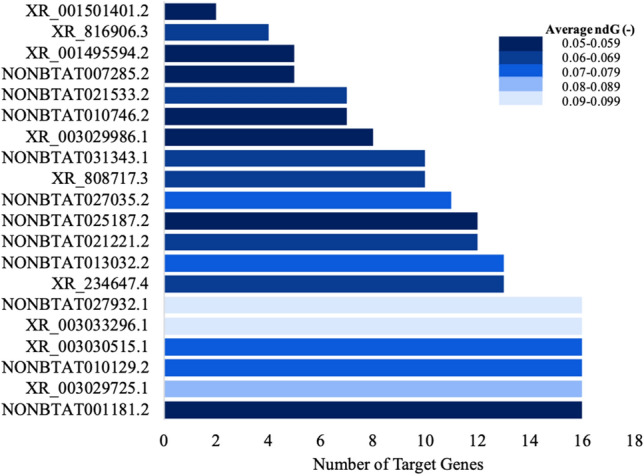
The 20 lncRNA and the number of target genes they are predicted to bind to. The darkest shade of blue corresponds to the lowest (-) ndG and the lightest shade of blue corresponds to the highest (-) ndG. Figure created with Microsoft Excel (2013) (https://www.microsoft.com/en-us/microsoft-365/excel).

**Figure 6:**
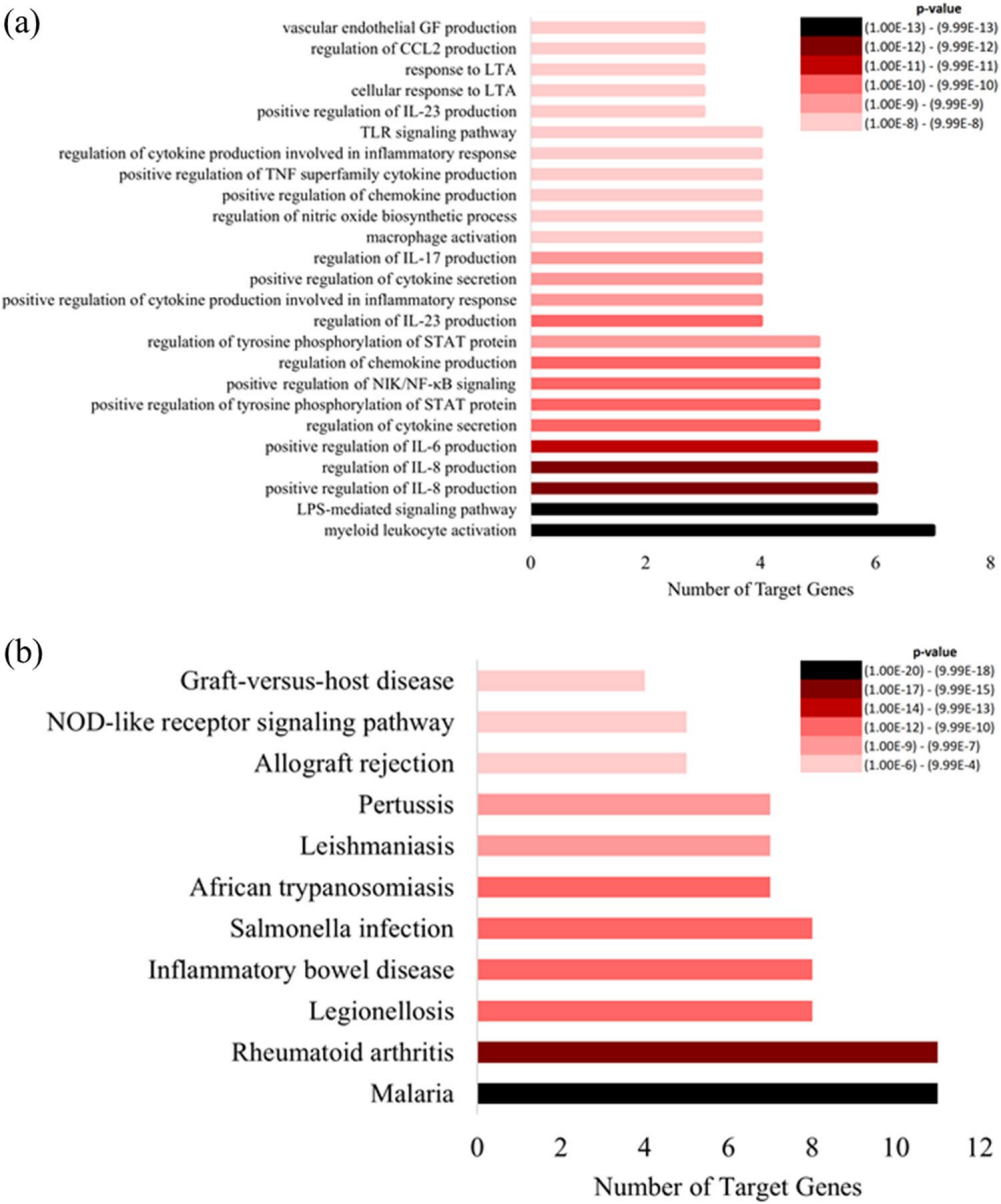
The biological processes and the number of target genes involved (**a**); and pathways and corresponding number of target genes (**b**). In both (**a**) and (**b**), black bars indicate biological processes/pathways predicted with the lowest *p*-value; lightest red bar represents predictions with the greatest *p*-value. Figure created with Microsoft Excel (2013) (https://www.microsoft.com/en-us/microsoft-365/excel).

NONCODE database search revealed 22 bovine lncRNAs that matched our prediction criteria. Genomic location and strand sense for each lncRNAs are shown in Table S4. From the analysis, we identified 20 lncRNAs that bind to our target mRNAs with a ndG < − 0.05 (Fig. 5; Table S4), with 8 of them targeting more than 13 of disease associated genes (Table 3). Of the 8 lncRNA, six (NONBTAT001181.2, XR_003029725.1, NONBTAT010129.2, XR_003030515.1, XR_003033296.1, NONBTAT027932.1) targeted all 16 genes, while NONBTAT027932.1, XR_003033296.1, and XR_003029725.1 could bind at least one target gene with an average ndG greater than -0.08. LncRNA NONBTAT001181.2-IL-18 and XR_003029725.1 showed a high affinity for IL-18 with thresholds less than − 0.1. Additionally, NONBTAT027932.1 could bind MYD88, CD4, ICAM1, CD14, TLR2, and TNFα with binding free energy less than − 0.1 (Table S4). Eight lncRNAs that bind 13 or more candidate genes with lower ndGs were selected for network and conservational analysis (Table 3). Most of the identified lncRNAs are not yet annotated, therefore, we inferred their gene ontology and pathways based on their potential mRNA targets/genes. Identified ontologies and associated pathways are shown in Table 4 and Fig. 6a and b.

**Table 3:** Significant lncRNA predicted to target the most candidate genes.

List of lncRNA	Number of target genes	List of target genes
NONBTAT001181.2	16	MYD88, CD4, IFNγ, IL-4, ICAM1, IL-18, CD86, CSF2, CCL2, TLR4, CXCL8, IL-10, IL-6, CD14, TLR2, TNFα
XR_003029725.1	16	MYD88, CD4, IFNγ, IL-4, ICAM1, IL-18, CD86, CSF2, CCL2, TLR4, CXCL8, IL-10, IL-6, CD14, TLR2, TNFα
NONBTAT010129.2	16	MYD88, CD4, IFNγ, IL-4, ICAM1, IL-18, CD86, CSF2, CCL2, TLR4, CXCL8, IL-10, IL-6, CD14, TLR2, TNFα
XR_003030515.1	16	MYD88, CD4, IFNγ, IL-4, ICAM1, IL-18, CD86, CSF2, CCL2, TLR4, CXCL8, IL-10, IL-6, CD14, TLR2, TNFα
XR_003033296.1	16	MYD88, CD4, IFNγ, IL-4, ICAM1, IL-18, CD86, CSF2, CCL2, TLR4, CXCL8, IL-10, IL-6, CD14, TLR2, TNFα
NONBTAT027932.1	16	MYD88, CD4, IFNγ, IL-4, ICAM1, IL-18, CD86, CSF2, CCL2, TLR4, CXCL8, IL-10, IL-6, CD14, TLR2, TNFα
XR_234647.4	13	MYD88, CD4, IFNγ, IL-4, ICAM1, IL-18, CSF2, TLR4, CXCL8, IL-10, CD14, TLR2, TNFα
NONBTAT013032.2	13	MYD88, CD4, ICAM1, IL-18, CD86, CSF2, CCL2, TLR4, CXCL8, IL-10, CD14, TLR2, TNFα

**Table 4:** Gene ontology biological processes, molecular functions, and cellular components of bovine mastitis target genes.

	Gene (s)	Raw *p*-value
**GO biological process**		
LPS-mediated signaling pathway	CCL2, TLR4, MYD88, CD14, IL-18, TNFα	2.58E−13
Myeloid leukocyte activation	IL-4, IFNγ, TLR4, CXCL8, IL-18, CSF2, TNFα	4.92E−13
Positive regulation of interleukin-8 production	TLR2, TLR4, MYD88, CD14, IL-6, TNFα	1.30E−12
Regulation of interleukin-8 production	TLR2, TLR4, MYD88, CD14, IL-6, TNFα	6.92E−12
Positive regulation of interleukin-6 production	TLR2, IFNγ, TLR4, MYD88, IL-6, TNFα	2.01E−11
Regulation of interleukin-23 production	IFNγ, TLR4, MYD88, CSF2	1.34E−10
Regulation of cytokine secretion	TLR2, IFNγ, CD14, IL-10, TNFα	2.50E−10
Positive regulation of tyrosine Phosphorylation of STAT protein	IL-4, IFNγ, IL-6, IL-18, CSF2	4.67E−10
Positive regulation of NIK/NF-kappaB signaling	TLR2, TLR4, CD14, IL-18, TNFα	5.65E−10
Regulation of chemokine production	TLR2, TLR4, MYD88, IL-6, TNFα	8.12E−10
Regulation of tyrosine phosphorylation of STAT protein	IL-4, IFNγ, IL-6, IL-18, CSF2	1.14E−09
Positive regulation of cytokine production involved in inflammatory response	TLR4, MYD88, IL-6, TNFα	4.01E−09
Positive regulation of cytokine secretion	IFNγ, CD14, IL-10, TNFα	4.70E−09
Regulation of interleukin-17 production	IFNγ, TLR4, MYD88, IL-18	6.36E−09
Macrophage activation	IL-4, IFNγ, TLR4, TNFα	1.40E−08
Regulation of nitric oxide biosynthetic process	IFNγ, TLR4, IL-10, TNFα	1.76E−08
Positive regulation of interleukin-23 production	IFNγ, MYD88, CSF2	2.30E−08
Positive regulation of chemokine production	TLR2, TLR4, IL-6, TNFα	2.98E−08
Response to lipoteichoic acid	TLR2, TLR4, CD14	3.45E−08
Cellular response to lipoteichoic acid	TLR2, TLR4, CD14	3.45E−08
Positive regulation of tumor necrosis factor superfamily cytokine production	IFNγ, TLR4, MYD88, CD14	3.61E−08
Regulation of cytokine production involved in inflammatory response	TLR4, MYD88, IL-6, TNFα	3.96E−08
Toll-like receptor signaling pathway	TLR2, TLR4, MYD88, CD14	5.16E−08
Vascular endothelial growth factor production	IL-6, TNF, TNFα	6.78E−08
Regulation of chemokine (C-X-C motif) ligand 2 production	TLR4, MYD88, TNFα	6.78E−08
**GO molecular function**		
NAD(P) + nucleosidase activity	TLR2, TLR4	2.64E-05
NAD + nucleotidase, cyclic ADP-ribose generating	TLR2, TLR4	2.64E-05
NAD + nucleosidase activity	TLR2, TLR4	2.64E-05
Toll-like receptor binding	TLR2, MYD88	3.86E-05
Lipopeptide binding	TLR2, CD14	8.93E-05
Pattern recognition receptor activity	TLR2, TLR4	9.97E-05
**GO cellular component**		
LPS receptor complex	TLR4, CD14	8.80E-06
Phagocytic cup	TLR4, TNFα	1.47E-04

### Biological, molecular and cellular function of target genes and pathway analysis

The pathway analysis of 16 target genes identified 25 biological processes (Fig. 6a). Myeloid leukocyte activation with a *p*-value of 4.92E−13, vascular endothelial growth factor (VEGF) production, regulation of chemokine (C-X-C motif) ligand 2 production and lipopolysaccharide (LPS)-mediated signaling pathway (*p*-value; 2.58E−13) (Table 4) are identified. Of importance are the three significant biological processes concomitantly identified from the three databases including LPS-mediated signaling pathway, regulation of IL-23 production, and positive regulation of chemokine production. DAVID analysis identified eight biological processes, three molecular functions and one cellular component, while 11 disease pathways were identified including malaria and rheumatoid arthritis that featured most candidate genes during bovine mastitis with *p*-values of 9.20E−19 and 4.60E−16, respectively (Fig. 6b; Table S5).

### Catalog of transcription factors, biological processes and molecular function

Seventeen TFs were identified as significant by at least two software (Table 5; Table S6). All 17 TFs appear to have the potential to regulate the transcription of the 16 genes including CD4, MYD88, ICAM1, TLR4, TLR2, IL-18, and CD86.The genomic location and other details of the 17 TFs are presented in Table 5. In addition, functional analysis identified 3 biological processes, 2 molecular functions, and 1 cellular component involving two to three TFs, with a *p*-value less than 0.0005 (Table 6).

**Table 5:** List of transcription factors predicted by AnimalTFDB and GeneXplain, their genomic location and strands.

COMMON TFs from the TWO database	Accession number	Genomic location	Strand (+ /−)
BCL6	ENSBTAG00000001511	1:79557257–79612240	(−)
CREB1	ENSBTAG00000005474	2:95845037–95898849	(−)
FOXA2	ENSBTAG00000012407	13:41542415–41547044	(−)
FOXM1	ENSBTAG00000015875	5:106886408–106900942	(−)
JUN	ENSBTAG00000004037	3:87265922–87268047	(−)
GATA1	ENSBTAG00000003184	X:86899691–86906632	(−)
HSF1	ENSBTAG00000020751	14:612908–634769	(+)
IRF1	ENSBTAG00000031231	7:21938453–21946840	(+)
SP1	ENSBTAG00000003021	5:26607078–26643492	(+)
SP2	ENSBTAG00000013740	19:38533093–38565741	(+)
SMAD1	ENSBTAG00000002835	17:12739131–12825260	(+)
TBP	ENSBTAG00000007686	9:104096534–104106636	(−)
TCF12	ENSBTAG00000002586	10:53023683–53085010	(−)
ZNF143	ENSG00000166478	11:9458955–9529888	(−)
ZNF274	ENSBTAG00000013353	18:65546272–65569269	(−)
ZNF384	ENSBTAG00000017072	5:103755842–103776263	(−)
ZNF92	ENSG00000146757	7:65373253–65401682	(−)

**Table 6:** Gene ontology biological processes, molecular functions, and cellular components of predicted TFs.

	TFs	Raw *p*-value
**GO biological process**		
Positive regulation by host of viral transcription	JUN, SP1	5.94E−05
Regulation of regulatory T cell differentiation	BCL6, IRF1	1.11E−04
Negative regulation of cell aging	FOXM1, BCL6	1.37E−04
**GO molecular function**		
Intronic transcription regulatory region sequence-specific DNA binding	BCL6, HSF1	4.31E−05
cAMP response element binding	JUN, CREB1	7.83E−05
**GO cellular component**		
Euchromatin	JUN, SP1, CREB1	1.11E−06

### Sequence conservation of bovine miRNAs and lncRNAs in other species

Sequence analysis was performed to assess the evolutionary conservation of bovine lncRNAs and miRNAs across various species (Table S1). We examined the significant miRNA (n = 6) and lncRNA (n = 8) identified previously with their homologous sequence from 14 other species. Our analysis of the six miRNAs reveal bta-miR-223 to be highly conserved in all 15 species, without a single deviant polymorphism (Fig S3). The evolutionary closeness of cow and wild yak is also seen in bta-miR-149-5p, bta-miR-328, and bta-miR-185, but are distantly related in bta-miR-24-3p and bta-miR-874 (Fig S4a–c). The phylogenetic analysis of bta-miR-223 defines cow having the closest identity with wild yak and the remaining ruminants (goat and sheep) as orthologs (Fig S4b). The alignment of bta-miR-874 showed high conservation between cow, mouse, and megabat, with phylogenetic analysis having these three sharing a common node. No other tree had these three species this close in proximity.

The length of lncRNA allow for greater variation within the gene throughout evolution. Analysis of the evolutionary alignment reveal that each lncRNA was not completely conserved in the 15 species; however, the majority have similar base pair sequences in conserved regions. Out of the 8 lncRNA, 7 have bovine lncRNA to be most closely related to wild yak, followed by other ruminants (Fig S5a–d). The tree of NONBTAT027932.1 show wild yak to be relatively distant from cow, sheep, and goat compared to the species relation in the other lncRNA analysis. Five out of eight lncRNA have human, gorilla, and chimpanzee clustered with the same origin.

### Dysregulation of lncRNA-miRNA interaction on immune response in bovine mastitis

**Figure 7:**
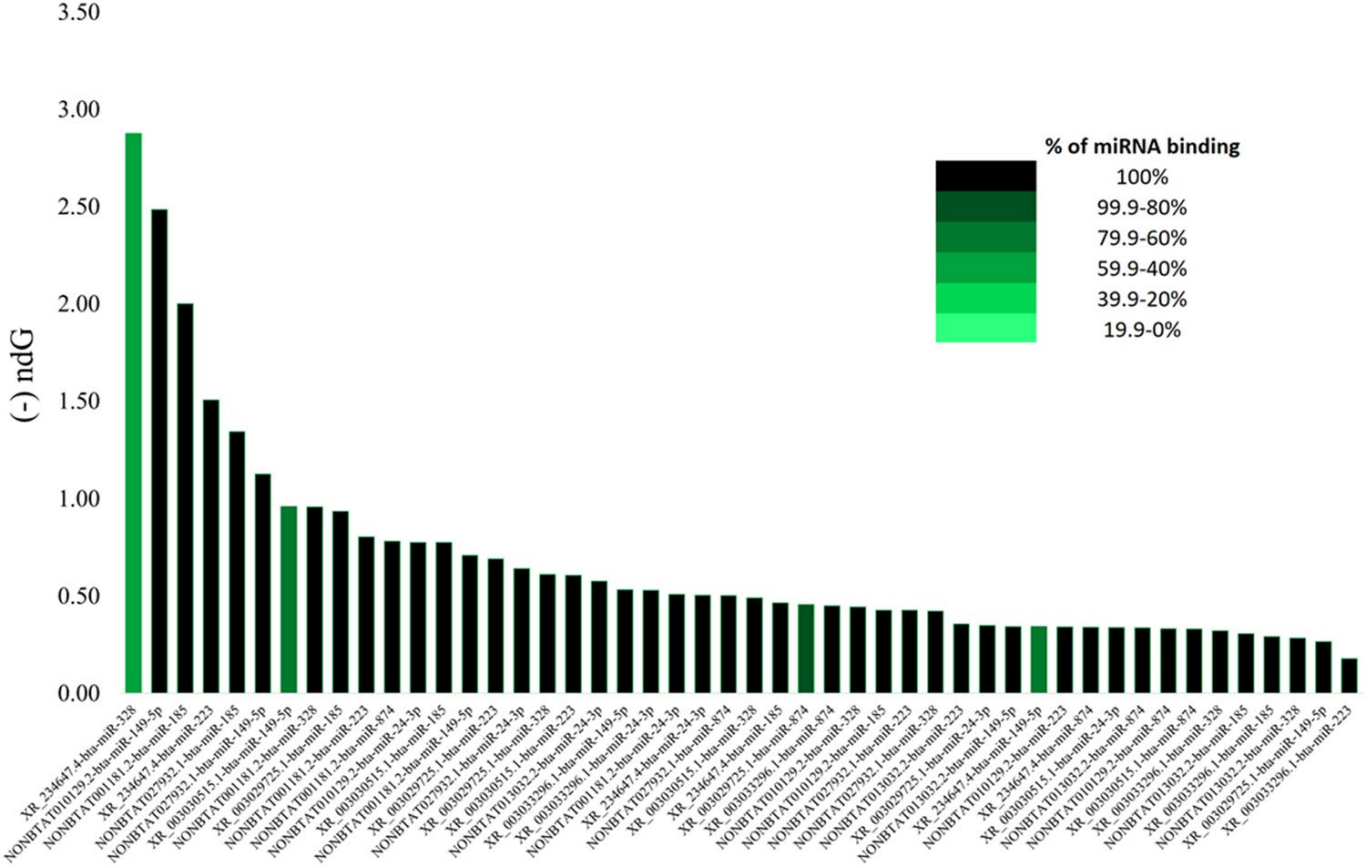
miRNA-lncRNA predicted binding with their normalized binding free energy (ndG). The black bars indicate the entire miRNA binds to the lncRNA; the lighter the green bar, the less complementary basing between miRNA and lncRNA. Figure created with Microsoft Excel (2013).

Our analysis showed several miRNA potential binding sites on lncRNA, based on the complementary base pairing and normalized binding free energies (ndG) values. Of interest, we found 8 out of 22 lncRNAs possessing binding sites for the entire length of these miRNAs, with the exception of 4 out of the 48 pairings (Fig. 7). The ndG values range from -0.1774 to -2.875. lncRNA XR_003033296.1 and bta-miR-223 had the greatest ndG while lncRNA XR_234647.4 and bta-miR-328 had the lowest ndG. The lower the ndG, the greater the possibility that the two RNA will bind. The vast majority of miRNA bind their entire length to the lncRNA except four, the shortest binding length is 10 (out of 22 base pairs) between bta-miR-223 and XR_234647.4. Additional results on lncRNA-miRNA binding are presented in Table S7.

### Combined network and molecular interactome of lncRNAs, miRNAs, TFs and immune genes

**Figure 8:**
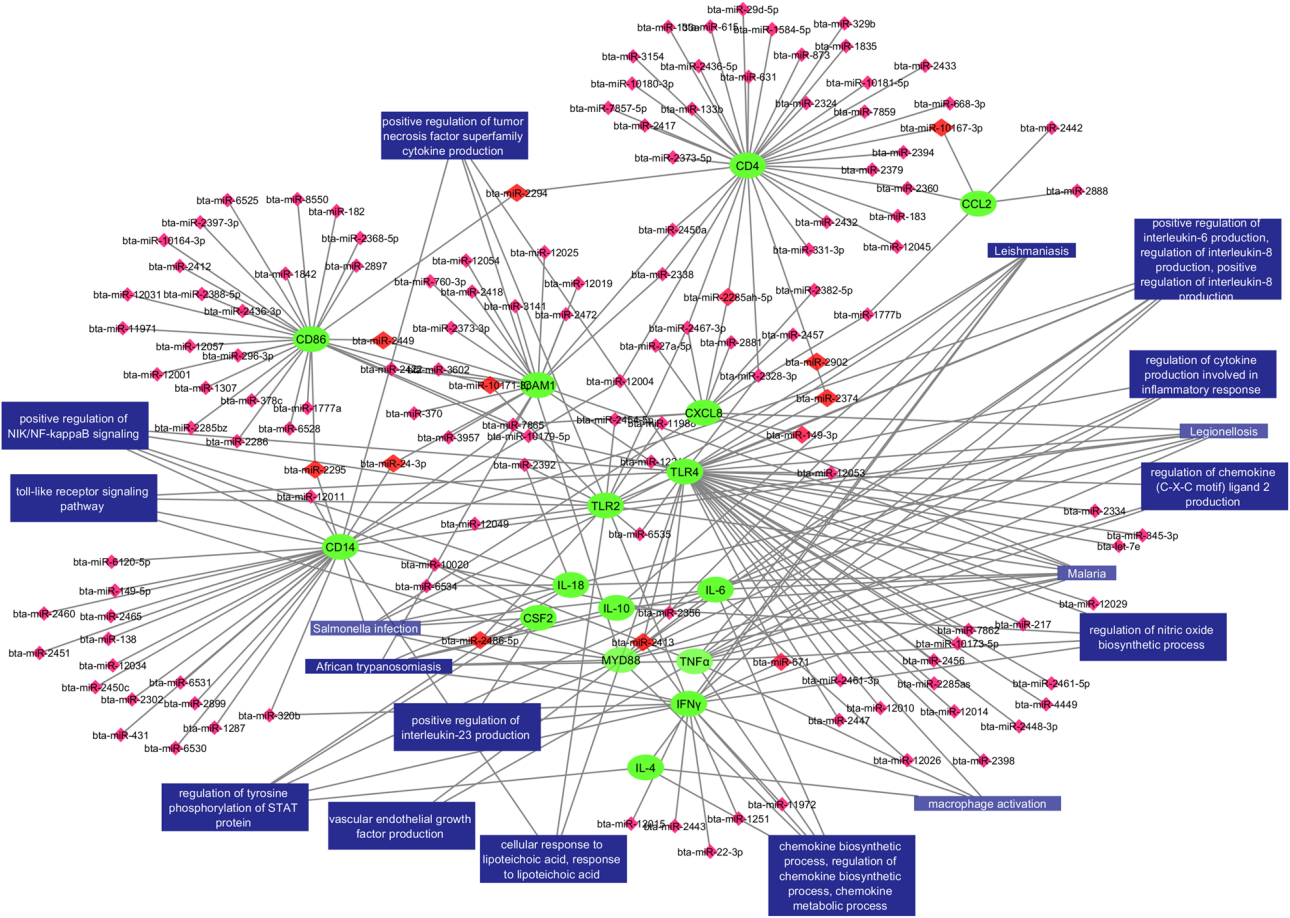
Network interactome of miRNA, their target genes, and the gene ontologies of the target genes. The pink diamonds are miRNA binding to a single target gene while the red diamonds are miRNA binding to two or more target genes. The green circles are the 16 target genes and the blue rectangles are the biological processes, molecular functions, cellular components, and pathways. (Cytoscape version 3.7.2; https://cytoscape.org/).

**Figure 9:**
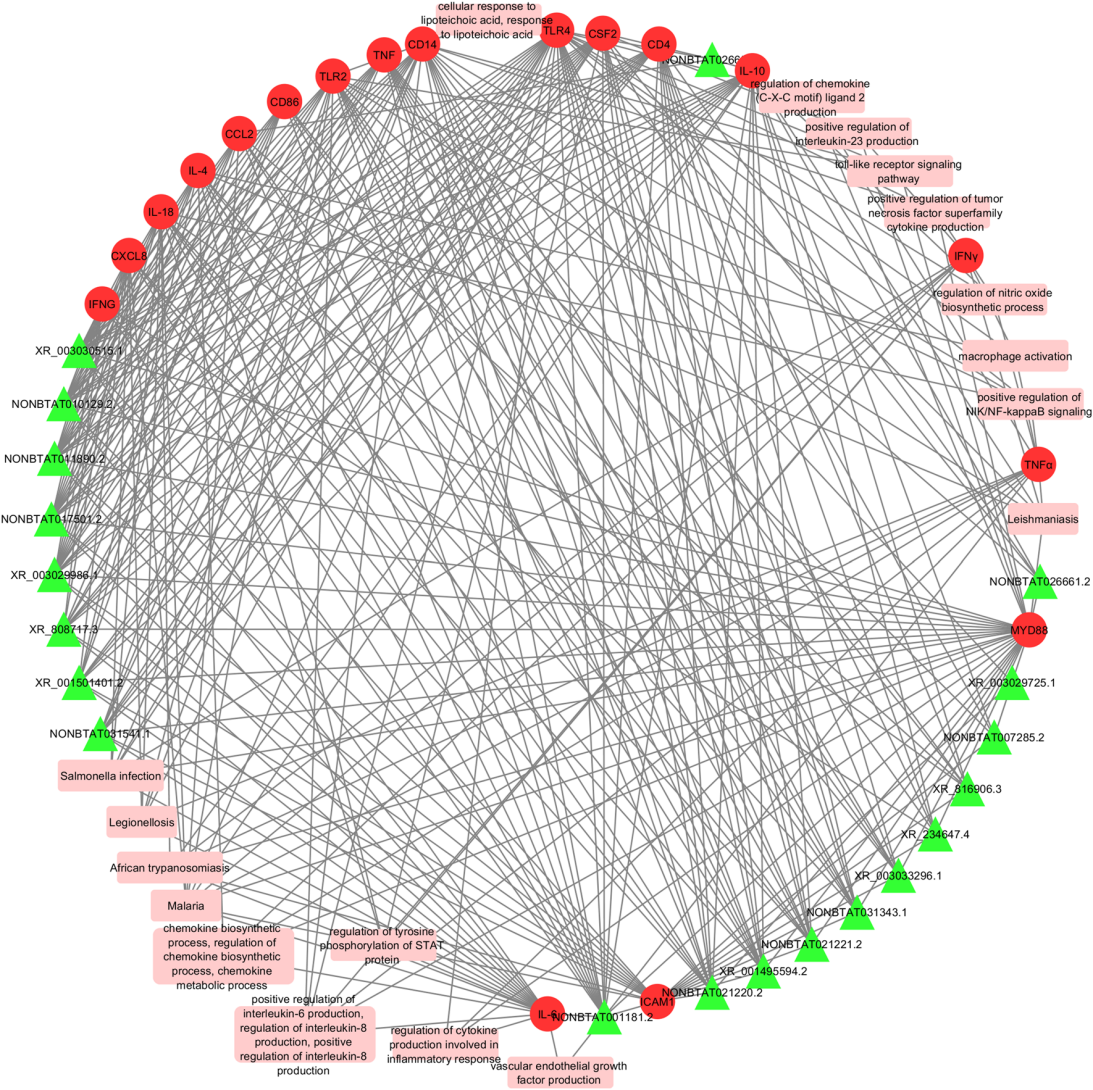
A network incorporating lncRNA, their target genes and corresponding gene ontologies. The green triangles are the lncRNA, red circles represent the target bovine mastitis genes, and the pink rectangles are the biological processes, molecular functions, cellular components, and pathways (Cytoscape version 3.7.2; https://cytoscape.org/).

**Figure 10:**
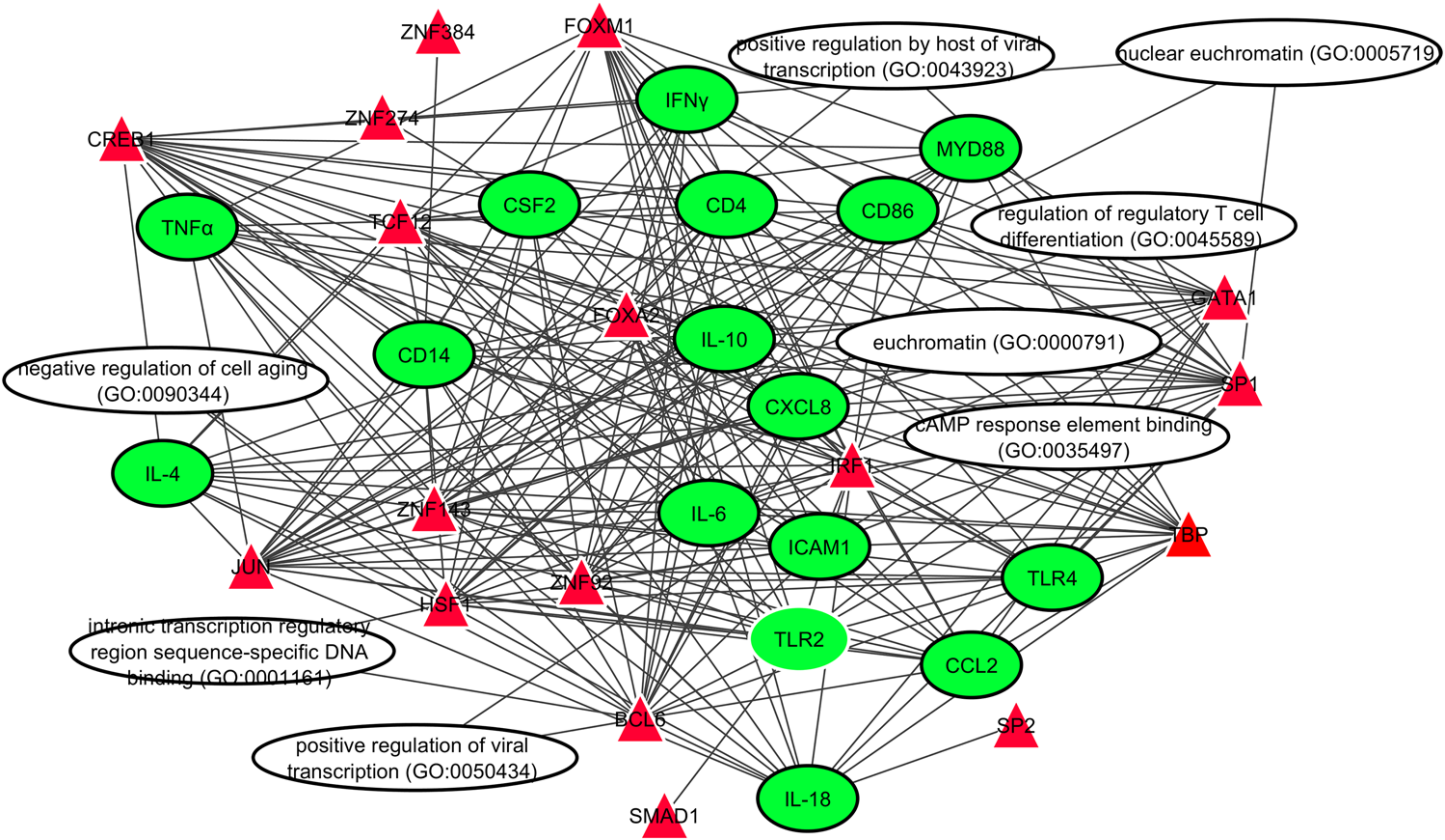
Network interactome of transcription factors (TFs), target genes, and gene ontologies. The red triangles are TFs; green circles are gene targets; while the ovals represent the biological processes, molecular functions, cellular components, and pathways (Cytoscape version 3.7.2; https://cytoscape.org/).

**Figure 11:**
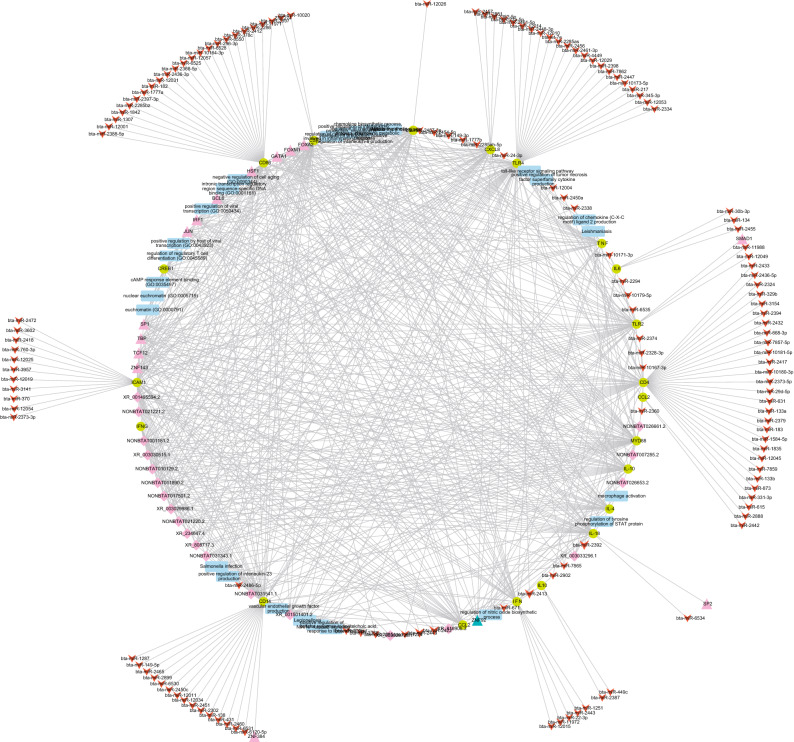
A network incorporating miRNA, lncRNA, TFs, target genes, and gene ontologies. The red arrowheads represent miRNA; purple diamonds represent lncRNA; teal triangles represent TFs; green circles represent target genes, and the blue rectangles biological processes, molecular functions, cellular components, and pathways (Cytoscape version 3.7.2; https://cytoscape.org/).

Figures 8, 9, 10 and 11 show molecular interactions between miRNAs, lncRNAs, TFs, target genes and pathways. Figure 8 in particular show the connections between the bovine mastitis target genes, miRNA, and the pathways/biological processes of the genes. CD4 (36) and TLR4 (36) have the most miRNAs targeting them, as opposed to IL-4, CSF2, and IL-18 (one, two and two miRNAs respectively). Some miRNAs are seen targeting more than one gene such as bta-miR-2294 (targeting CD4 and CD86) and bta-miR-2374 (targeting TLR4 and CD4). Interestingly, we found some miRNAs that bind to multiple genes involved in the same pathway, such as bta-miR-671 targeting TLR4 and IFNγ, both of which are involved in macrophage activation; bta-miR-2413 targeting IFNγ, IL-10, and TLR4, all of which are involved in the regulation of nitric oxide biosynthetic process.

The functional annotation of each lncRNA was inferred from the target genes using GO Enrichment Analysis and the DAVID KEGG algorithm program. Several of these lncRNAs are involved in multiple ontologies (Fig. 9). Specifically, NONBTAT001181.2 could bind to IL-6 to regulate its expression, thereby perturbing functional pathways like vascular endothelial growth factor, regulation of cytokine production, which are involved in inflammatory response, and regulation of tyrosine phosphorylation of STAT protein pathways. LncRNA XR_003030515.1 targets TLR4 and is connected to cellular response to lipoteichoic acid, macrophage activation, NAD + nucleosidase activity, and regulation of interleukin-23 production. LncRNA NONBTAT027932.1 targets CD14 and is involved in positive regulation of NIK/NF-κB signaling, regulation of interleukin-8 production, regulation of cytokine secretion, LPS-mediated signaling pathway, lipopeptide binding, and LPS receptor complex (Fig. 9). Figure 10 shows the interconnections between the 17 TFs, 16 target genes, and the pathways they are involved in. Both CREB1 and JUN have numerous connections directed toward the target genes and pathways.

The final immunoregulatory integrated network in our analysis contains miRNAs, lncRNAs, TFs, target genes, and biological pathways and their extensive interconnections (Fig. 11). We show an important connectivity between lncRNAs, miRNAs, bovine mastitis immune genes and that their biological functions have all been mapped on the network. The outer circle contains mostly miRNAs, with the exception of 3 TFs, that bind only to a single target gene. The inner circle consists of miRNAs binding to more than one target gene, lncRNAs, TFs, target genes, and biological pathways. LncRNAs are found densely in the top inner circle and can be sporadically located in the outer circle. Our results indicate that the majority of lncRNAs, miRNAs, and TFs interactions overlapped and are involved in several pathways, including biological processes, cellular components, and molecular functions.

## Discussion

Bovine mastitis is detrimental to the dairy industry with significant impact on farms in developing countries^26,27^. Mastitis can become chronic and highly contagious, and the effective treatment options available are suboptimal, one reason being the cost associated with antibiotic residue in the milk^28^. Regulatory factors have been associated with other diseases, such as the identification of a lncRNA that may be an intuitive preventive and therapeutic treatment option for atherosclerosis^29^. Novel key lncRNA and TFs were identified in lung adenocarcinoma regulatory networks, offering new drug targets for therapeutic treatment^30^. In another study, miR-99b was found to repress the expression of IGF1R and mTOR, ultimately acting as an onco-suppressor in clear cell renal cell carcinoma^31^. To our knowledge, this is the first report examining all three regulatory elements (miRNA, lncRNA and TFs) in the context of bovine mastitis, with the aim to identify elements influencing expression of candidate genes regulating immune response.

Through our systematic review and computational mining, we generated a list of genes playing critical roles in immune response during bovine mastitis. TLR2 and TLR4 were substantially connected to other candidate genes through our network, which is expected given their involvement in pro-inflammatory and innate immune response^32^. Specifically, TLR4 recognizes lipopolysaccharides found in the outer membrane of gram-negative bacteria and TLR2 recognizes lipoteichoic acid found in the cell wall of gram-positive bacteria^33^. Pathogen recognition is imperative to the initiation of the immune response, and without TLR4 and TLR2, identification of bacteria in bovine mammary epithelial cells would be impaired. IL-8, IL-6 and TNFα, the three most common genes in our analysis, are all inflammatory cytokines or chemokines, and have been shown to be upregulated in bovine mammary epithelial cells stimulated with LPS^34^.

Of importance, our results identified six miRNAs binding to several target genes with the potential to regulate their expression during infection. Interestingly, previous studies have shown bta-miR-24-3p, bta-miR-328, bta-miR-223, and bta-miR-185to be associated with bovine immune response. Bta-miR-24-3p has been associated with serum antibody response to *Mycoplasma bovis* in beef cattle^35^. Bta-miR-185 had increased expression in milk from a *S. aureus* infected cow compared to a healthy cow. Ma et al.^36^ identified bta-miR-185 targeting five genes including MLLT3, DYRK1B and NPR2. Other studies have demonstrated the involvement of bta-miR-223 in bovine mastitis immune response^32,37^, and also in other immune responses during lung infection, bacterial peritonitis, and cutaneous leishmaniasis^38–41^. The other two miRNAs identified in our study, bta-mir-149-5p and bta-miR-874, have been associated with fat levels in beef cattle^42,43^. Our findings, along with previous publications, collectively demonstrate the involvement of these miRNAs in immune response and should be strongly considered for further laboratory studies. In addition, our study demonstrated that miRNAs that could bind to all three regions on a single gene had a greater probability of binding the target gene, and ultimately perturb the disease pathways the target genes are involved in.

Furthermore, we identified important interactions between certain lncRNAs and mRNAs that could mediate host immune response at the post-transcriptional level. LncRNAss are becoming recognized for their involvement in several biogenetic processes that may regulate disease pathogenesis^14,44^. We hypothesized that lncRNAs that bind to multiple mRNA target sequences could serve as drug targets and have the potential to modify or regulate immune signaling pathways, ameliorating the over- or under-production of cytokines or chemokine during infection. Importantly, lncRNAs could directly bind to the mRNA, causing degradation and prevent translation to the expected protein. Of interest, we also found several lncRNAs with binding sites for miRNAs, for example XR_003033296.1-bta-miR-223 and lncRNA XR_234647.4-bta-miR-328. These interactions could prevent the negative regulatory effect of miRNA on the mRNA targets during disease perturbation. Gene ontology analysis reveal several of these miRNAs and lncRNAs to be associated with myeloid leukocyte activation, LPS-mediated signaling pathway, and positive regulation of IL-8 production. These miRNAs and lncRNAs interactions may be an avenue to manipulate the regulatory elements involved in immune response to bovine mastitis. The knowledge is potentially useful in disease detection/diagnosis, especially in subclinical cases and may be an ideal target for treatment^15,45^.

Additionally, our pathway analysis revealed seven genes, IL-4, IFNγ, TLR4, CXCL8, IL-18, CSF2 and TNFα, are involved in the morphological and behavioral changes of macrophages, monocytes, granulocytes, and dendritic cells (DCs) and their recruitment into infection sites. Therefore, the miRNA binding to these seven gene transcripts, degrading their mRNA in the process, would have a deleterious effect on the immune response needed to eliminate the pathogen. Our study indicated bta-miR-328 and bta-miR-223 bind to three out of the seven genes involved in this pathway. A recent study found the overexpression of bta-miR-223 in Mac T cells attenuating the inflammatory response induced by lipoteichoic acid derived from *S. aureus*, by targeting the Cbl proto-oncogeneB (CBLB), positively correlated with the expression of P13K, AKT, and NF-κB p65^37^. Fang and others concluded that bta-miR-223 is a central post-transcriptional regulator in mammary tissue when infected with *S. aureus* by altering the expression of key innate immune response genes, one being CXCL14^32^. Other studies have also identified bta-miR-223 as a key miRNA expressed in mammary tissue during bovine mastitis^46^. Given our findings and previous reports, we surmise that these six miRNAs, especially bta-miR-223 would play significant dysregulatory role on immune response during bovine mastitis and could serve as biomarker or drug target, deserving further in vivo and in vitro investigation to clarify.

Our study showed seven significant transcription factors including SP1, SF1 and JUN targeting several genes within the biological pathways. SP1, JUN and CREB are commonly found in almost all the significant biological pathways (molecular function, biological and cellular processes), resulting from their function as activators and repressors. SP1 has been identified as one of the first eukaryotic trans activators to regulate numerous mammalian genes, often in association with other transcription factors, playing major roles, including cell cycle regulation, hormonal activation and apoptosis^47,48^. JUN directly transcribes the expression of epidermal growth factor receptor thereby positively regulating the proliferation and differentiation of keratinocytes^49^.

Nuclear transcription factor-κ (NF-κB) signaling is a major player in the regulation of innate and adaptive immune responses and is widely studied for its role in mastitis progression and pathogenesis. Many inflammatory genes contain binding sites for NF-κB in their promoter regions, thus active NF-κB regulates expression by directly binding to target genes in the nucleus^4^. Cytokines (IL-6, IL-1β, TNFα) and chemokine (CXCL8) are associated with increased expression in LPS-stimulated mammary glands^8,50,51^. These pro-inflammatory cells disrupt the blood-milk barrier causing slowed milk production and painful clinical manifestations in cattle with mastitis. Given the immunological and inflammatory importance, lncRNAs targeting mediators (TLR2, TLR4, MYD88, CD14, ICAM-1, IL-6, IL-1β, TNFα, and CXCL8) of the NF-κB signaling pathway may act as biomarkers for disease diagnosis and drug therapeutics by blocking lncRNAs from interacting with the target genes.

A variety of growth factors and inflammatory signals induce CREB and subsequently mediate the transcription of genes that contain a cAMP-responsive element. Interestingly, we report that miRNAs could bind and regulate IL-6, IL-10, and TNFα, which possess the cAMP-responsive element^52^. IRF1, through the regulation of autoimmunity, inflammation, viral infections, and innate and adaptive immune responses, is also responsible for the protection of host cells^53^.

Conservation analysis reveal all six miRNAs to be almost entirely conserved between the 15 species. The bta-miR-223 was highly conserved in all 15 species, without a single base pair difference. The conservation of these miRNAs throughout evolution indicates their importance and benefits to the host, further validating our results. These findings also suggest a drug targeting one of these miRNAs can be used across multiple species. This also suggests the possibility of experimental drug trials on smaller animals that have a higher rate of reproduction, such as a mouse or rat, following in vivo/in vitro elucidation.

Network interactome of miRNA, target genes, pathways, and cellular functions give a visualization of the connections between them, allowing for comprehension of the broad picture- regulatory element to target gene to biological pathway. For example, bta-miR-671 is predicted to bind to IFNγ and TLR4 in at least two of the three mRNA regions, and both IFNγ and TLR4 are involved in the positive regulation of tumor necrosis factor superfamily cytokine production. Therefore, bta-miR-671 may be a strong target gene as it affects a biological pathway through two different genes. One of our identified miRNAs, bta-miR-24-3p binds to CD14 in at least two regions. CD14 is involved in cellular response to lipoteichoic acid, toll-like receptor signaling pathway, positive regulation of tumor necrosis factor superfamily cytokine production, positive regulation of cytokine secretion, positive regulation of NIK/NF-κB signaling, regulation of interleukin-8 production, and LPS-mediated signaling pathway, all of which play major roles in immune response. Bta-miR-149-3p targets both CXCL8 and TLR4, both of which are involved in myeloid leukocyte activity. This interactome ties our findings together and allows for miRNA to be seen as possible therapeutic targets.

## Conclusions

The present study provides computational evidence that miRNA, lncRNA, and TFs could regulate the host immune response to bovine mastitis. These regulatory element interactomes provide an insight into immune gene dysregulation during infection. The knowledge, which is useful in disease diagnosis, especially in subclinical cases, may be ideal targets for antibiotic treatment. Our data indicated 16 immune-related genes as key components involved in host immune response to bovine mastitis. We identified 6 miRNA, 8 lncRNA, and 17 TFs that could potentially regulate expression of our candidate genes and were deemed significant to our study. Of importance, we reported miRNA bta-miR-223 to be highly conserved across species and targeting multiple genes. Likewise, 6 lncRNAs including NONBTAT027932.1 and XR_003029725.1 bind to all candidate genes while SP1, JUN, and CREB transcription factors were involved in a majority of ontology processes. The crosstalk between these regulatory elements was particularly important to our study, providing a new direction for disease studies in farm animals. Functional network analyses reveal complex interactions, suggesting that our identified significant elements would be effective drug targets for treatment and prevention through vaccination. To validate these predictions, further work is needed with in vitro and in vivo analyses that examine the expression or regulatory role of miRNAs, lncRNAs and TFs on host immune response in bovine mastitis.

## Methods

### Extraction and verification of significantly regulated genes in bovine mastitis

The analytical pipeline employed for this study, starting from literature search to interaction networks (Fig. 1a), including some modifications, have been described previously^7,54^. Briefly, to obtain experimentally validated genes associated with bovine mastitis, we carried out an extensive search for significantly mentioned genes, utilizing several databases. First, LitInspector (Genomatix version 3.10, Munich, Germany) was used to scan all literature-curated datasets derived from high and low throughput experiments with a Medical Subject Headings (MeSH) on bovine mastitis, as described^7^. We obtained a MeSH-Term ID of C22.196.5819 and a p-value of 48E-31. Identified gene sets were filtered based on the number of reports from Google Scholar and Web of Science articles, pathway enrichment and gene ontology (GO)^55^. Second, we carried out a systematic review on PubMed (https://www.ncbi.nlm.nih.gov/pubmed/) with combination of keywords including bovine mastitis + gene expression, to catalog genes from published literature in the database. We matched the identified genes from both search methods with Bioinformatics and Evolutionary Genomics (BEG) Venn diagram generator (http://bioinformatics.psb.ugent.be/webtools/Venn/) to identify common genes. The overlapped genes between the two lists were selected as our candidate genes (total of 16 genes) and were included for further analysis (Table 1). We then constructed a protein–protein interaction network of the candidate genes with Search Tool for the Retrieval of Interacting Genes database (STRING-DB; string-db.org) to determine molecular interactions and possible mechanisms for co-expression and interconnectivity, from both text mining and experimentally determined datasets^56^.

### Prediction of miRNA for bovine mastitis candidate genes

The locations of the 16 identified genes from our analysis were determined within the *Bos taurus* reference genome (ARS-UCD1.2) and complete transcript sequences were downloaded from GenBank. The transcript information and accession numbers are listed in Table 1. Furthermore, using *Bos taurus* as the reference organism, we searched for miRNAs that could bind complementarily against the entire region of each of our candidate genes, including the 5′-UTR, CDS, and 3′-UTR, using the prediction software miRWalk^20^. MiRNA binding to any of the three regions with a maximum p-value of 1 was used for further selection. To evade false positives in our analysis, the generated list of miRNAs was cross referenced with two other prediction software, miRNet^21^ and TargetScan^22^ as described^7^. Using Venn generator, the overlap of predicted miRNA from the three software were selected as significant for further analysis (Table 2).

### Identification of lncRNAs associated with immune response during bovine mastitis

In order to identify putative lncRNAs within cattle genome that may be binding to the transcript sequences thereby regulating expression, we performed a genome-wide search based on the publicly available curated data from four databases: NONCODE^24^, a comprehensive knowledge based non-coding RNA database that specifically focuses on lncRNA; LncTar^19^ a lncRNA-RNA prediction software using the minimum binding free energy between RNA sequences; Ensembl (http://useast.ensembl.org/index.html), a vertebrate genome browser supporting analysis software, and LncRNA2Target v2.0^25^, a comprehensive database of lncRNA-target relationships, manually curated from published lncRNA-focused articles. Briefly, we searched each database for lncRNAs using cow as reference. Due to the high number of lncRNAs within the genome, we made some assumptions and applied the most stringent filter criteria using: 1. With the assumption more exons imply high quality transcripts, we selected only lncRNAs with exon number greater than two. 2. Assuming longer transcript length gives better quality transcripts, transcript length > 200 bp were selected. 3. Knowing a lower prediction score means a higher quality transcript; prediction score less than − 0.2 was used. 4. Finally, we selected only lncRNAs that meet criteria 1–3 and were also found in minimum of three out of the four databases. Briefly, complete sequences of each lncRNA were retrieved from NONCODE and each sequence searched on the bovine genome ARS-UCD1.2. using the Ensembl BLAT/BLAST genomic sequencing tools. NCBI BLAST was used to identify available accession numbers for each lncRNA (Table 3). Using LncTar, each mRNA gene was used as target to determine if bovine lncRNAs bind to the mRNA targets. Based on the normalized free binding energy (ndG) value from the nucleotide base pairing, dataset with less than − 0.05 were considered significant and used as candidates for further analysis^19^. To avoid false positive lncRNAs, we used LncRNA2Target v2.0 as a second lncRNA-mRNA prediction software with human homology for the 16 target genes to determine bovine lncRNA binding sites. We located all identified human lncRNAs counterparts on the bovine genome ARS-UCD1.2. The lncRNAs found in bovine genome ARS-UCD1.2 and the human genome GRCh38 e were significant to our study.

### Prediction of significant transcription factors

Transcription factors and their corresponding binding sites were predicted for the genes using different prediction software; AnimalTFDB^57^ and GeneXplain^58^, so as to increase result accuracy. AnimalTFDB was employed using the default settings with the *p*-value cut off at the minimum of e-06. For GeneXplain, both Match and P-Match algorithms were adjusted to use the cut off selection “1.0 and 1.0” as matrix similarity cut-off and core similarity cut-off. We included only seventeen TFs that appeared in the results of at least two software for further analysis.

### Functional and pathway analysis of candidate genes associated with immune response to bovine mastitis

Functional enrichment analysis, using geneontology.org, was performed on the 16 candidate genes to determine the molecular functions, biological processes and cellular components the genes are involved in as described^14,59^, with slight changes. Using the fold enrichment of greater than 100, the false discovery rate (FDR) of < 10^−5^, and the *p*-value of < 10^−7^, we catalogue the biological processes and cellular components. The molecular functions were selected based on the following criteria; fold enrichment > 100, FDR < 0.001, and the *p*-value was < 0.01. To validate these findings, we carried out functional analysis with Database for Annotation, Visualization and Integrated Discovery (DAVID)^60^, using the same parameters as stated previously. Results common in both programs were used for further analysis. Significant pathways were generated using KEGG program algorithms with fold enrichment of > 40, while the p-value and FDR were < 10^−4^.

### Sequence conservation analysis of significant miRNA and lncRNA

Using miRBase^61^, candidate miRNA sequences were retrieved and ascertained using BLAST against cow, dog, cat, pig, human, gorilla, chicken, goat, rat, megabat, horse, mouse, wild yak, sheep, and chimpanzee for conservation analysis (Table S1). MEGAX^62^ and MultiAlin^63^ were used to align miRNA sequences from each species to determine the regions of conservation and construct a phylogenetic tree. The tree was exported as a Newick text-file and uploaded to iTOL^64^ for further editing. Furthermore, lncRNA sequences extracted from NONCODE were compared using multiple sequence alignment and phylogenetic analyses to detect evolutionary profile across 15 species using MEGA X. The phylogenetic trees were also imported to iTOL for further editing and visualization.

### Prediction of lncRNA-miRNA interactions associated with bovine mastitis pathogenesis

We hypothesized that a complementary base pairing or binding of miRNA and lncRNA would be an important mechanism to manipulate the regulatory elements involved in immune response to bovine mastitis. To do this, we predicted the possible miRNA binding sites on the lncRNA sequences and determine their normalized binding free energy (ndG) based on the complementary base pairing, using LncTar^19^. Furthermore, miRNA, lncRNA, their target genes, and the biological processes were connected on a critical network using Cytoscape version 3.7.2, as described previously^54,65^.

### Combined interactome network of lncRNAs, miRNAs, TFs and immune gene targets

To provide information on the connectivity as well as to identify the molecular mechanisms for gene regulation between the candidate genes, miRNAs, lncRNAs, and TFs, we created an interactome network with Cytoscape software, version 3.7.2. Network of different nodes were created based on all identified RNAs, TFs and associated pathways, while the network edges determine the relationship between the regulatory elements and pathways. Our interactome modeling took the advantage of modeling information in process pathways of Database for Annotation, Visualization and Integrated Discovery (DAVID), Gene ontology (GO) and KEGG (www.genome.jp/kegg), creating new global biological associations within the dataset.

## Supplementary Information

Supplementary Legends.

Supplementary Figure S1.

Supplementary Figure S2.

Supplementary Figure S3.

Supplementary Figure S4.

Supplementary Figure S4.

Supplementary Figure S4.

Supplementary Figure S5.

Supplementary Figure S5.

Supplementary Figure S5.

Supplementary Figure S5.

Supplementary Table S1.

Supplementary Table S2.

Supplementary Table S3.

Supplementary Table S4.

Supplementary Table S5.

Supplementary Table S6.

Supplementary Table S7.

## Data Availability

The datasets analyzed in this study are available from multiple repositories (see accession numbers in Tables 1, 2, and 5). Additional accession numbers are provided in Tables S1 and S4.
