# Supplementary material for: Regulatory network of miRNA, lncRNA, transcription factor and target immune response genes in bovine mastitis
Source: Sci Rep. 2021 Nov 9;11:21899. doi: 10.1038/s41598-021-01280-9 (PMC8578396; doi:10.1038/s41598-021-01280-9)
Supplement: Supplementary file 1 — Supplementary Legends. [file 41598_2021_1280_MOESM1_ESM.docx]

**Supplementary Figure Legends**

**Figure S1.** Venn diagram (<http://bioinformatics.psb.ugent.be/webtools/Venn/>) of bovine mastitis immune genes harvested from systematic review and Genomatix, meta-data. In total was 919 genes identified from the systematic review (purple) and 20 from Genomatix software (pink). The overlap region represents the 16 target bovine mastitis genes used for further analysis in this study.

**Figure S2.** Venn diagram (<http://bioinformatics.psb.ugent.be/webtools/Venn/>) involving the three miRNAs prediction software; miRWalk, miRNet, and TargetScan. The blue circle represents the number of miRNAs predicted by miRWalk (693), pink miRNet (47), and green Target Scan (146). The center region represents the six miRNAs used for further analysis in this study.

**Figure S3.** Multiple sequence alignment of the six predicted bovine miRNAs and corresponding genes in 15 other species using MultiAlin^62^ (<http://multalin.toulouse.inra.fr/multalin/>); bta-miR-24-3p, bta-miR-149-5p, bta-miR-185, bta-miR-223, bta-miR-328, and bta-miR-874. The highlighted yellow region within the gene sequences represents the miRNA sequence. Red indicates regions of high consensus and blue low consensus.

**Figure S4a-c.** Evolutionary analysis of the six miRNAs generated from the multiple sequence alignment using MEGA-X^62^ and iTOL (<https://itol.embl.de/>) (A-C); phylogenetic trees of bta-miR-24-3p and bta-miR-149-5p and their corresponding gene in the 15 other species (A); phylogenetic trees of bta-miR-185 and bta-miR-223 (B); phylogenetic trees of bta-miR-328 and bta-miR-874 (C).

**Figure S5a-d.** Evolutionary analysis of the eight lncRNA generated from multiple sequence alignment using MEGA-X^62^ and iTOL(<https://itol.embl.de/>) (A-D); phylogenetic trees of NONBTAT001181.2 and NONBTAT007847.2 (A); phylogenetic trees of NONBTAT011890.2 and NONBTAT010129.2 (B); phylogenetic trees of NONBTAT013032.2 and NONBTAT017501.2 (C); and phylogenetic trees for NONBTAT021220.2 and NONBTAT027932.1.
