# Supplementary material for: Regulatory network of miRNA, lncRNA, transcription factor and target immune response genes in bovine mastitis
Source: Sci Rep. 2021 Nov 9;11:21899. doi: 10.1038/s41598-021-01280-9 (PMC8578396; doi:10.1038/s41598-021-01280-9)
Supplement: Supplementary file 2 — Supplementary Figure S1. [file 41598_2021_1280_MOESM2_ESM.pdf]

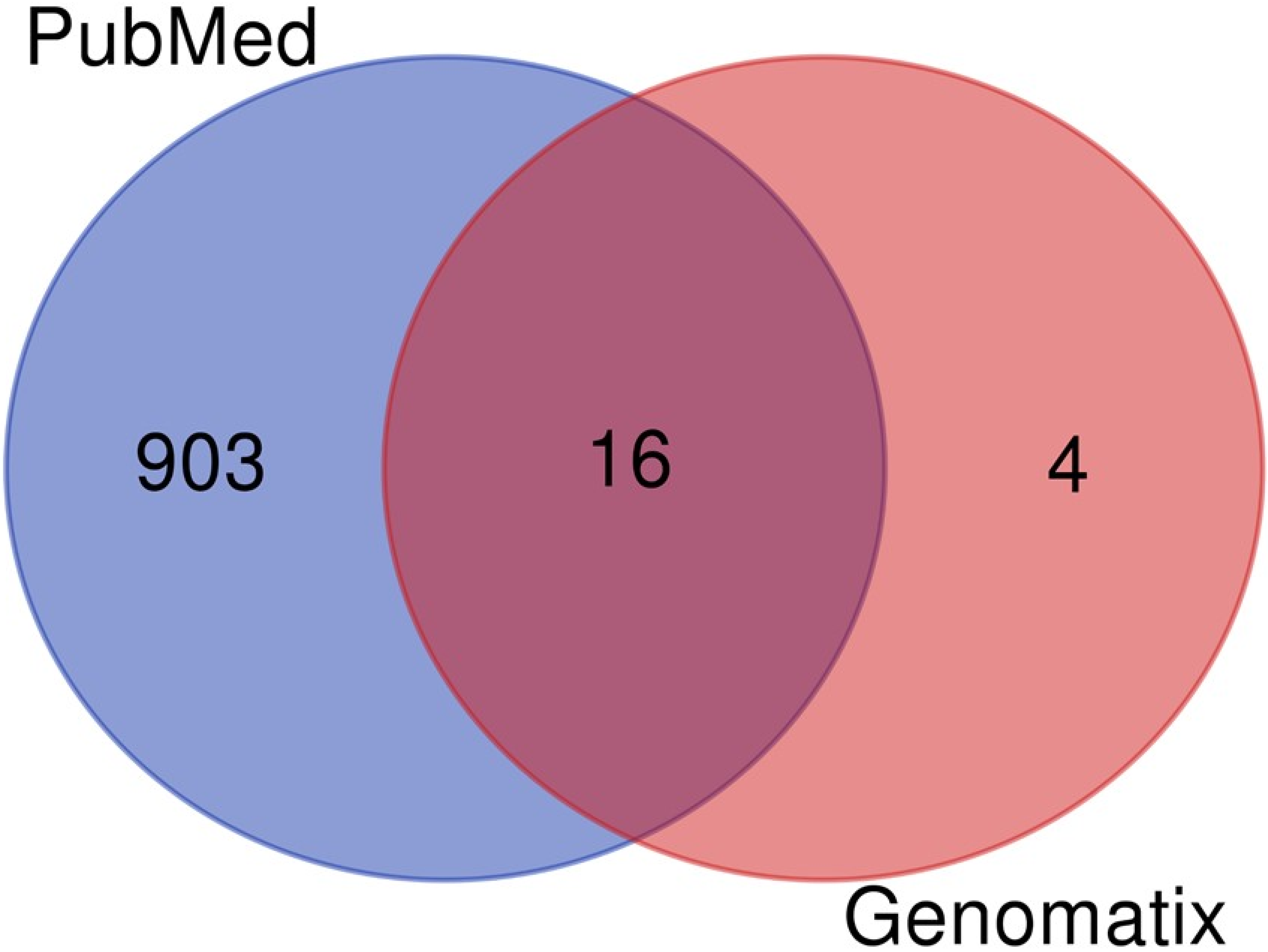

Supplementary Figure 1. Venn diagram of bovine mastitis immune genes harvested from meta-analysis and Genomatix. In total was 919 genes identified from the meta-analysis (purple) and 20 from Genomatix software (pink). The overlap region represents the 16 target bovine mastitis genes used for further analysis in this study.
