# Supplementary material for: Regulatory network of miRNA, lncRNA, transcription factor and target immune response genes in bovine mastitis
Source: Sci Rep. 2021 Nov 9;11:21899. doi: 10.1038/s41598-021-01280-9 (PMC8578396; doi:10.1038/s41598-021-01280-9)
Supplement: Supplementary file 3 — Supplementary Figure S2. [file 41598_2021_1280_MOESM3_ESM.pdf]

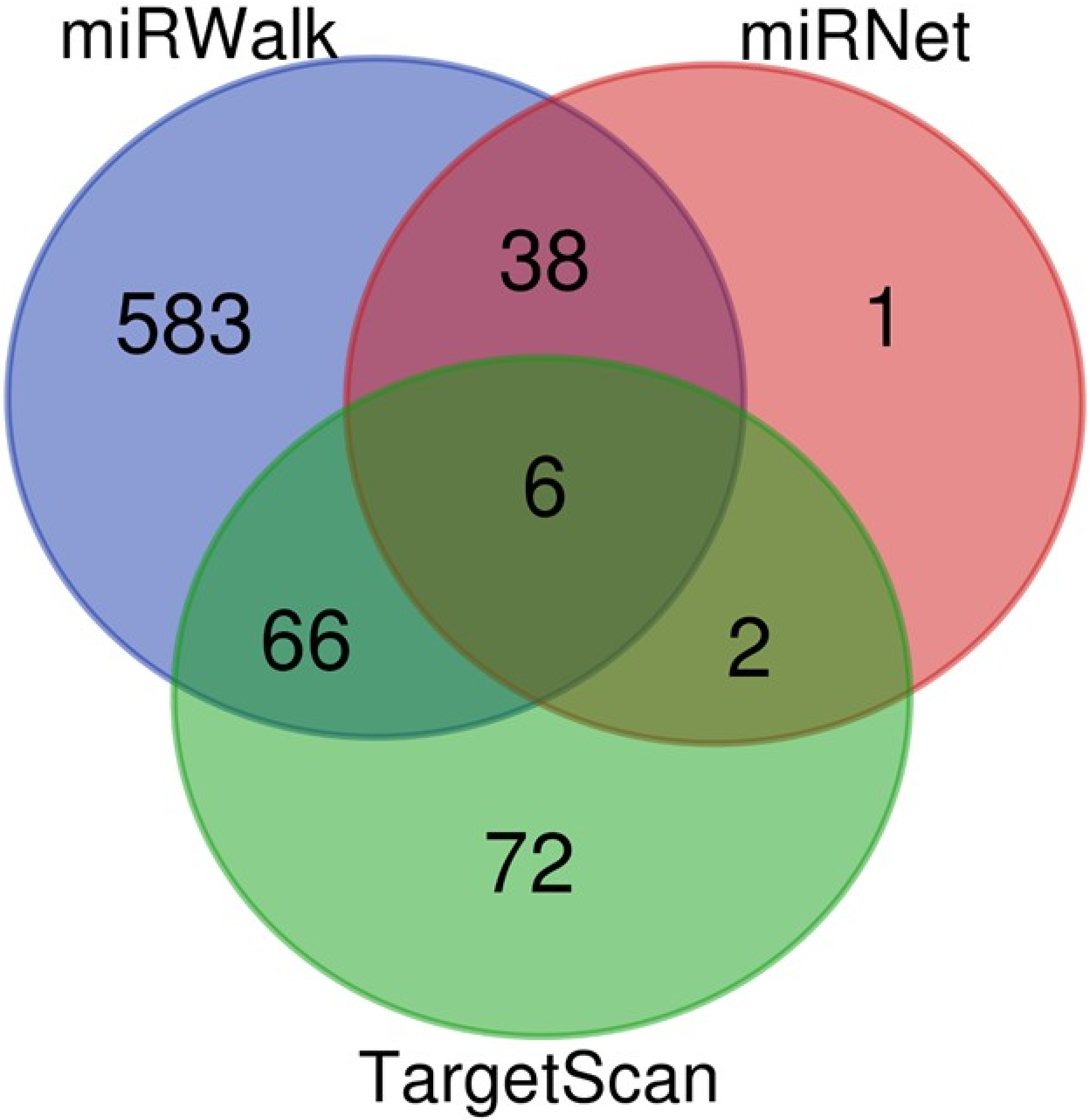

Supplementary Figure 2. Venn diagram involving the three miRNAs prediction software; miRWalk, miRNet, and TargetScan. The blue circle represents the number of miRNA predicted by miRWalk (693), pink miRNet (47), and green Target Scan (146). The center region represents the six miRNAs used for further analysis in this study.
