# Supplementary material for: Regulatory network of miRNA, lncRNA, transcription factor and target immune response genes in bovine mastitis
Source: Sci Rep. 2021 Nov 9;11:21899. doi: 10.1038/s41598-021-01280-9 (PMC8578396; doi:10.1038/s41598-021-01280-9)
Supplement: Supplementary file 4 — Supplementary Figure S3. [file 41598_2021_1280_MOESM4_ESM.pdf]

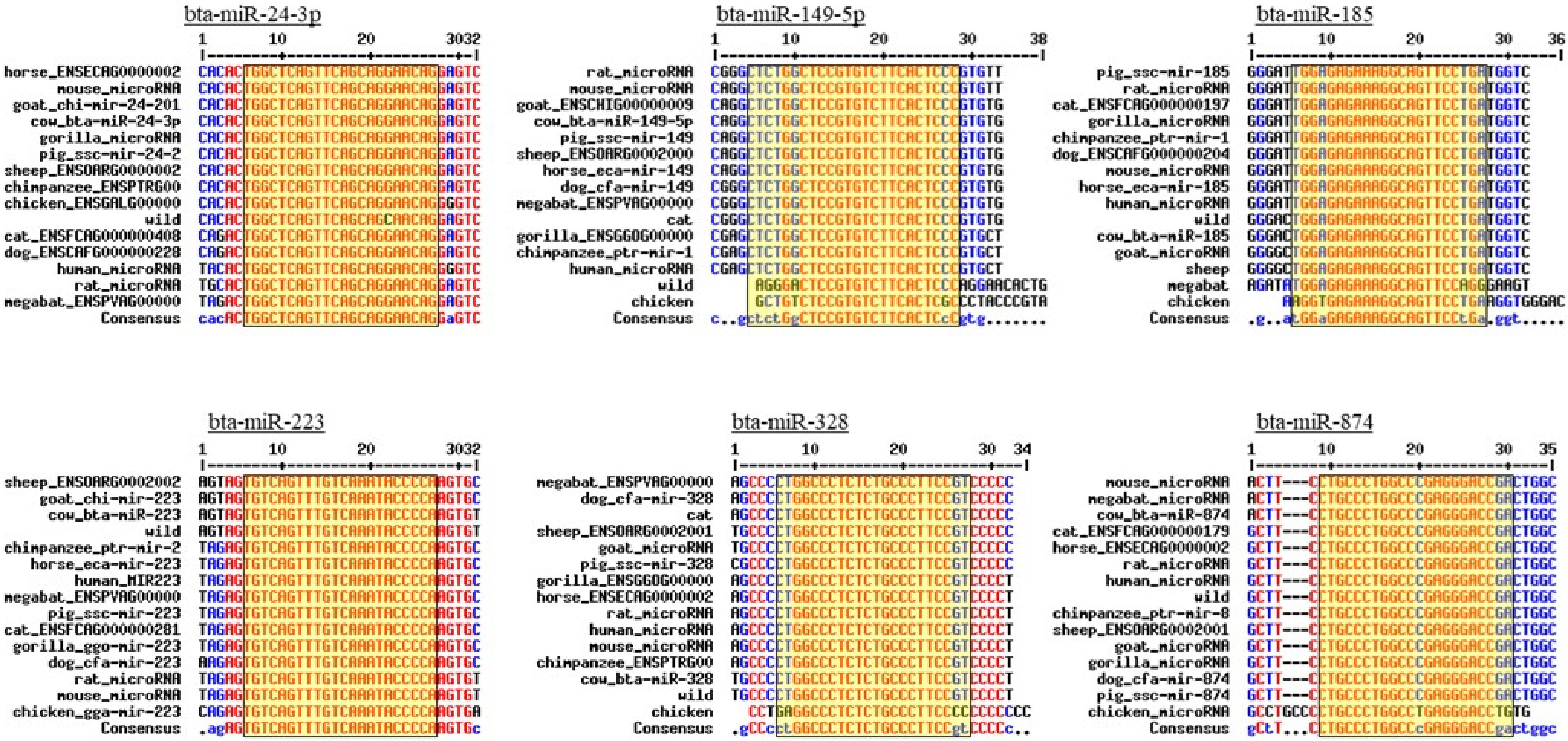

Supplementary Figure 3. Multiple sequence alignment of the six predicted bovine miRNAs and corresponding genes in 15 other species using MultiAlin; bta-miR-24-3p, bta-miR-149-5p, bta-miR-185, bta-miR-223, bta-miR-328, and bta-miR-874. The highlighted yellow region within the gene sequences represent the miRNA sequence. Red indicates regions of high consensus and blue low consensus.
