# Supplementary material for: Regulatory network of miRNA, lncRNA, transcription factor and target immune response genes in bovine mastitis
Source: Sci Rep. 2021 Nov 9;11:21899. doi: 10.1038/s41598-021-01280-9 (PMC8578396; doi:10.1038/s41598-021-01280-9)
Supplement: Supplementary file 7 — Supplementary Figure S4. [file 41598_2021_1280_MOESM7_ESM.pdf]

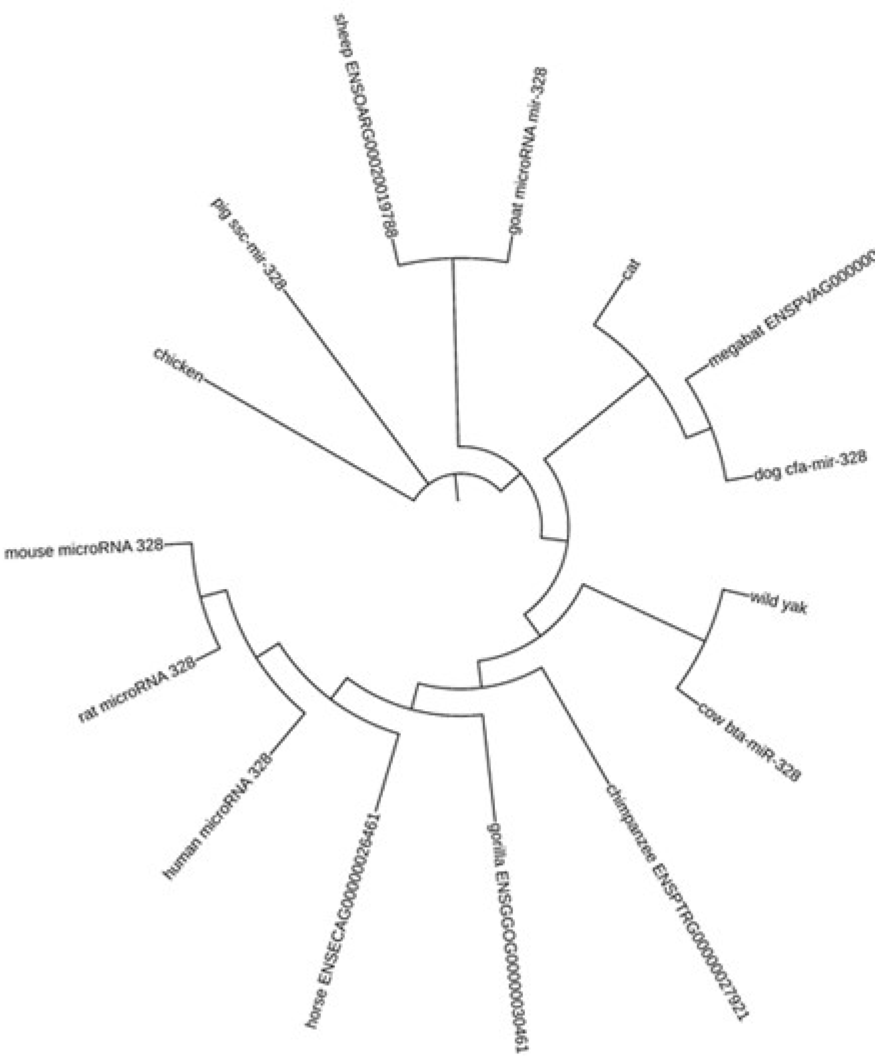

bta-miR-328

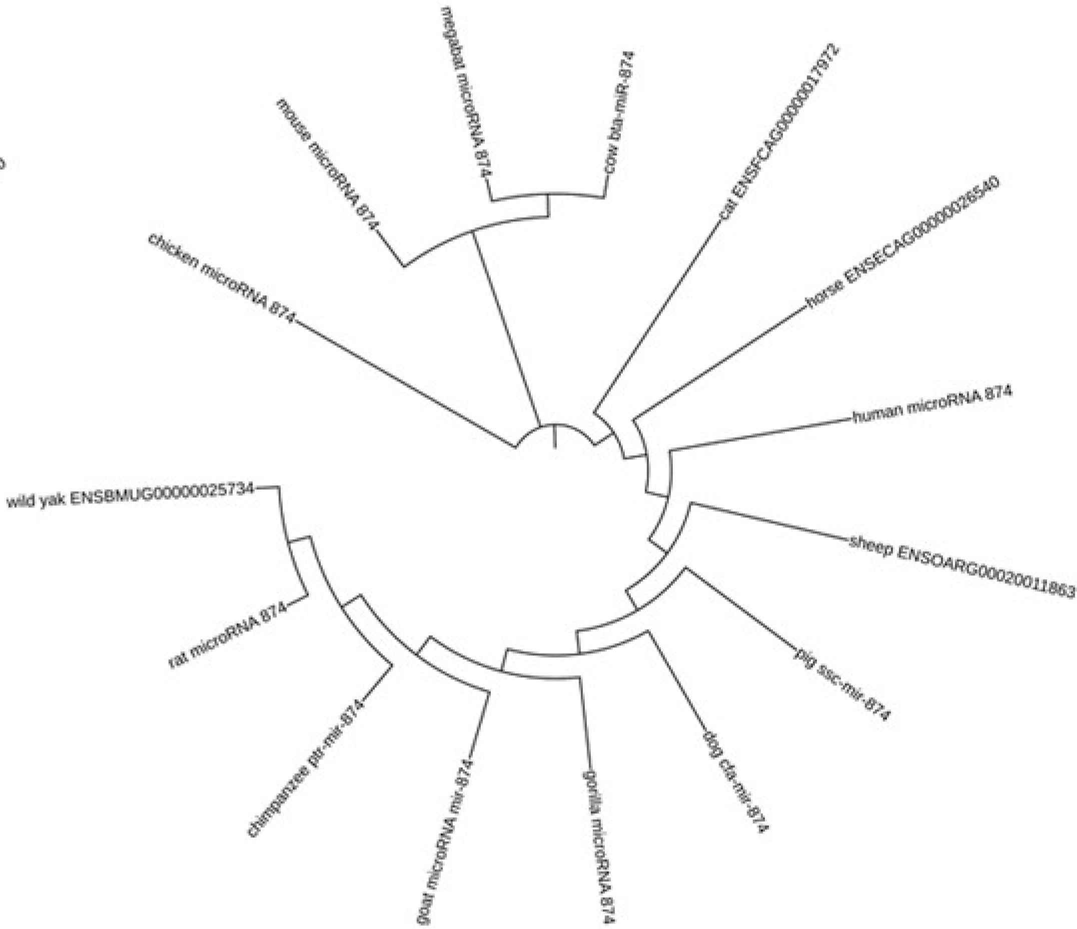

bta-miR-874

Supplementary Figure 4a-c. Evolutionary analysis of the six miRNAs generated from the multiple sequence alignment using MEGA-X and iTOL(A-C); phylogenetic trees of bta-miR-24-3p and bta-miR-149-5p and their corresponding gene in the 15 other species (A); phylogenetic trees of bta-miR-185 and bta-miR-223 (B); phylogenetic trees of bta-miR-328 and bta-miR-874 (C).
