# Supplementary figures and images for: Regulatory network of miRNA, lncRNA, transcription factor and target immune response genes in bovine mastitis
Source: Sci Rep. 2021 Nov 9;11:21899. doi: 10.1038/s41598-021-01280-9 (PMC8578396; doi:10.1038/s41598-021-01280-9)

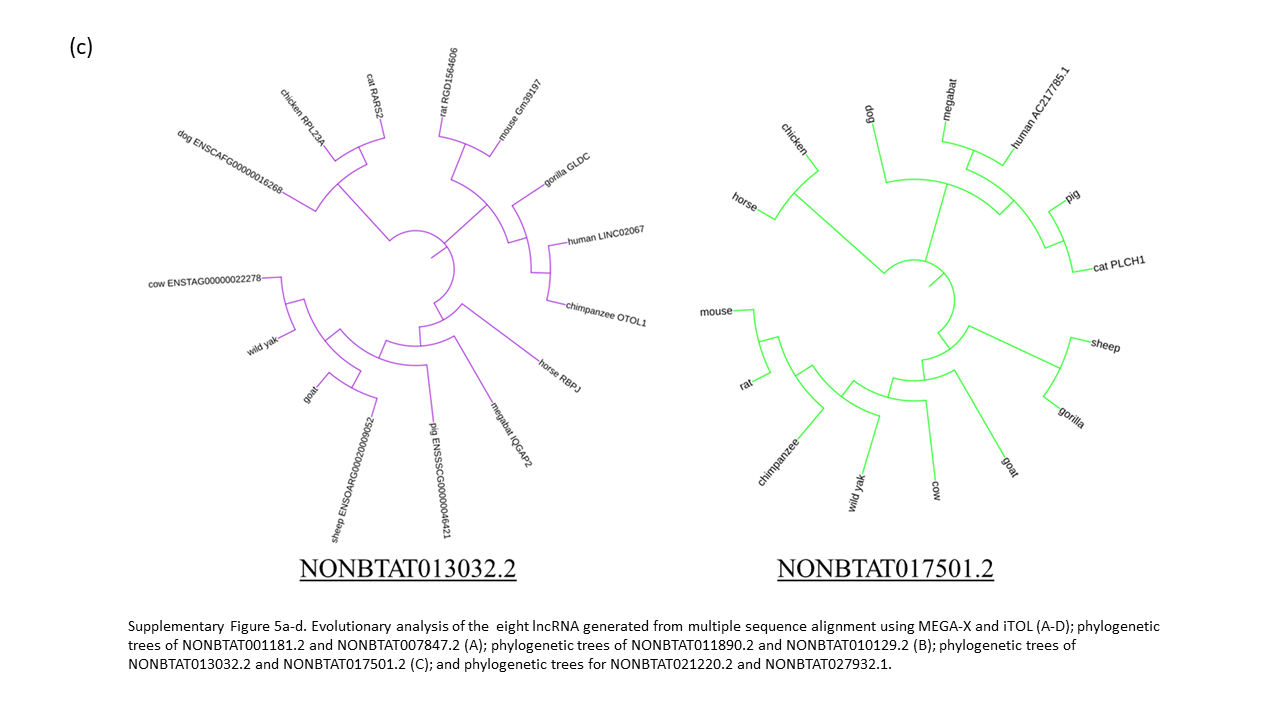

Supplement: Supplementary file 10 — Supplementary Figure S5. [file 41598_2021_1280_MOESM10_ESM.png]
