# Supplementary material for: Regulatory network of miRNA, lncRNA, transcription factor and target immune response genes in bovine mastitis
Source: Sci Rep. 2021 Nov 9;11:21899. doi: 10.1038/s41598-021-01280-9 (PMC8578396; doi:10.1038/s41598-021-01280-9)
Supplement: Supplementary file 11 — Supplementary Figure S5. [file 41598_2021_1280_MOESM11_ESM.pdf]

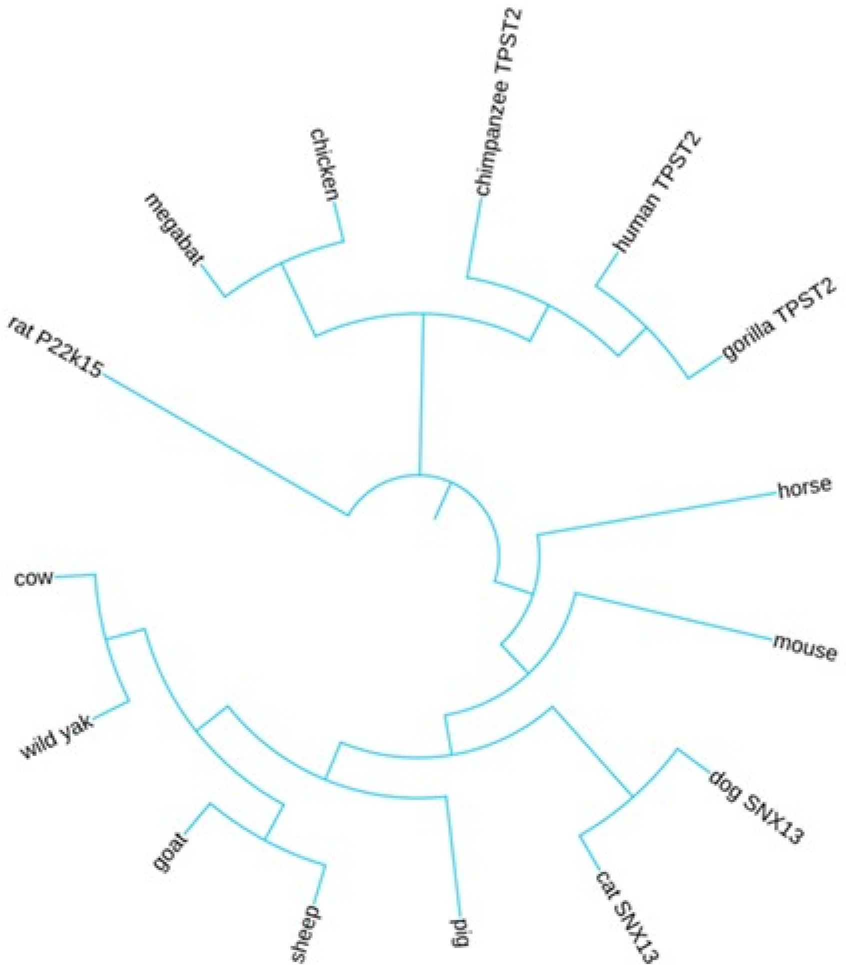

NONBTAT021220.2

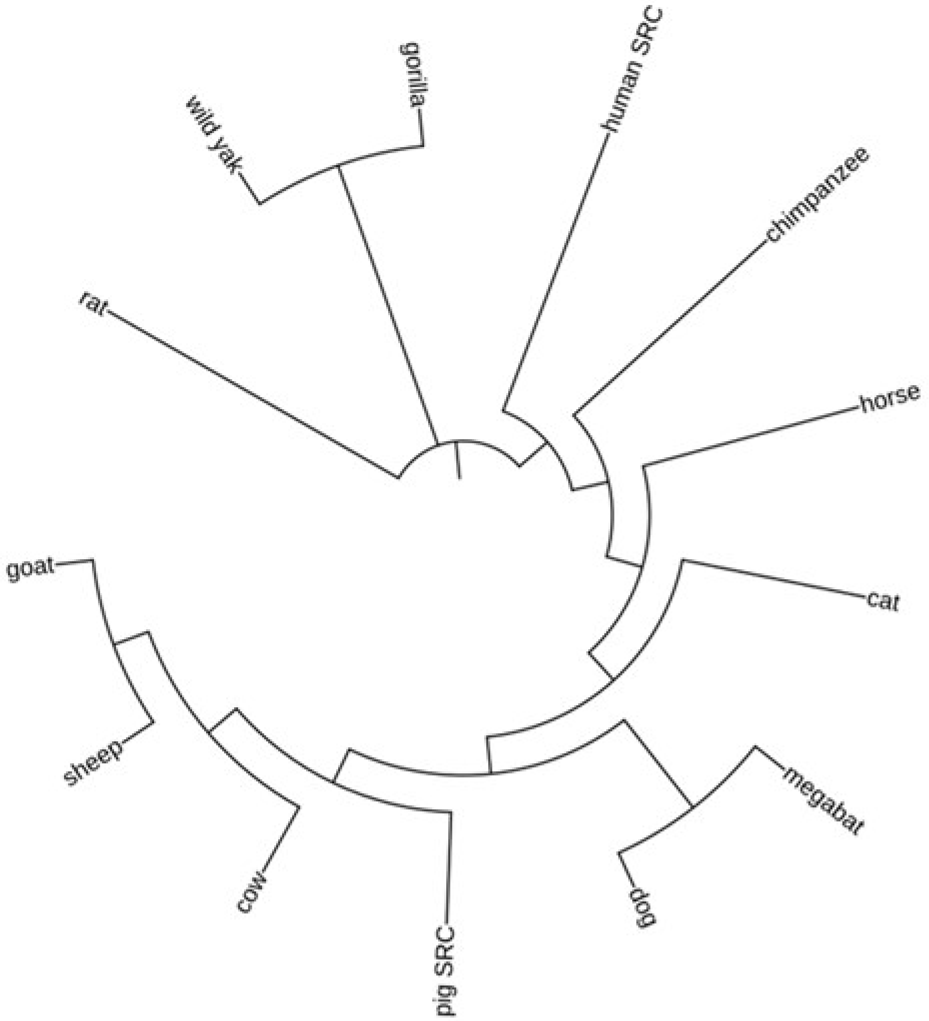

NONBTAT027932.1

Supplementary Figure 5a-d. Evolutionary analysis of the eight lncRNA generated from multiple sequence alignment using MEGA-X and iTOL (A-D); phylogenetic trees of NONBTAT001181.2 and NONBTAT007847.2 (A); phylogenetic trees of NONBTAT011890.2 and NONBTAT010129.2 (B); phylogenetic trees of NONBTAT013032.2 and NONBTAT017501.2 (C); and phylogenetic trees for NONBTAT021220.2 and NONBTAT027932.1.
