# Supplementary material for: Regulatory network of miRNA, lncRNA, transcription factor and target immune response genes in bovine mastitis
Source: Sci Rep. 2021 Nov 9;11:21899. doi: 10.1038/s41598-021-01280-9 (PMC8578396; doi:10.1038/s41598-021-01280-9)
Supplement: Supplementary file 12 — Supplementary Table S1. [file 41598_2021_1280_MOESM12_ESM.docx]

| **Species** | **Binomial Nomenclature** | **Assembly ID** | **Transcript ID** |
| --- | --- | --- | --- |
| Cow | Bos taurus | ARS-UCD1.2 | GCA_002263795.2 |
| Cat | Felis catus | Felis_catus_9.0 | GCA_000181335.4 |
| Chicken | Gallus gallus | GRCg6a | GCA_000002315.5 |
| Chimpanzee | Pan troglodytes | Pan_tro_3.0 | GCA_000001515.5 |
| Dog | Canis lupus familiaris | CanFam3.1 | GCA_000002285.2 |
| Goat | Capra hircus | ARS1 | GCA_001704415.1 |
| Gorilla | Gorilla gorilla gorilla | gorGor4 | GCA_000151905.3 |
| Horse | Equus caballus | EquCab3.0 | GCA_002863925.1 |
| Human | Homo sapiens | GRCh38.p13 | GCA_000001405.28 |
| Megabat | Pteropus vampyrus | pteVam1 | GCA_000151845.1 |
| Mouse | Mus musculus | GRCm38.p6 | GCA_000001635.8 |
| Pig | Sus scrofa | Sscrofa11.1 | GCA_000003025.6 |
| Rat | Rattus norvegicus | Rnor_6.0 | GCA_000001895.4 |
| Sheep | Ovis aries | Oar_rambouillet_v1.0 | GCA_002742125.1 |

**Supplementary Table 1.** Characteristics of species sequences used to create phylogenetic trees for candidate lncRNA and miRNA.
