# Supplementary material for: Regulatory network of miRNA, lncRNA, transcription factor and target immune response genes in bovine mastitis
Source: Sci Rep. 2021 Nov 9;11:21899. doi: 10.1038/s41598-021-01280-9 (PMC8578396; doi:10.1038/s41598-021-01280-9)
Supplement: Supplementary file 13 — Supplementary Table S2. [file 41598_2021_1280_MOESM13_ESM.docx]

S**upplementary Table 2.** miRNA and their target gene as predicted by miRWalk**.**

| **3’UTR Binding Region** | | | | **CDS Binding Region** | | | | | | **5’UTR Binding Site** | | | |
| --- | --- | --- | --- | --- | --- | --- | --- | --- | --- | --- | --- | --- | --- |
| **miRNA** | **Target Gene** | **# of Pairings** | **Binding Region Length** | | **miRNA** | **Target Gene** | **# of Pairings** | **Binding Region Length** | **miRNA** | | **Target Gene** | **# of Pairings** | **Binding Region Length** |
| bta-miR-221 | CD86 | 19 | 20 | | bta-miR-103 | CD86 | 15 | 17 | bta-miR-453 | | CD86 | 16 | 21 |
| bta-miR-27a-5p | CD86 | 19 | 27 | | bta-miR-21-3p | CD86 | 15 | 16 | bta-miR-2295 | | CD86 | 13 | 15 |
| bta-miR-27a-3p | CD86 | 12 | 13 | | bta-miR-107 | CD86 | 15 | 18 | bta-miR-2299-3p | | CD86 | 16 | 19 |
| bta-miR-484 | CD86 | 18 | 26 | | bta-miR-15b | CD86 | 17 | 20 | bta-miR-2322-5p | | CD86 | 15 | 18 |
| bta-miR-484 | CD86 | 15 | 18 | | bta-let-7d | CD86 | 13 | 18 | bta-miR-2364 | | CD86 | 16 | 19 |
| bta-miR-128 | CD86 | 16 | 23 | | bta-miR-124a | CD86 | 11 | 12 | bta-miR-2388-5p | | CD86 | 15 | 21 |
| bta-miR-199a-5p | CD86 | 17 | 22 | | bta-miR-148b | CD86 | 19 | 25 | bta-miR-2893 | | CD86 | 10 | 15 |
| bta-miR-27b | CD86 | 12 | 13 | | bta-miR-93 | CD86 | 20 | 26 | bta-miR-2897 | | CD86 | 16 | 22 |
| bta-miR-31 | CD86 | 15 | 25 | | bta-miR-23b-5p | CD86 | 14 | 19 | bta-miR-2450d | | CD86 | 17 | 23 |
| bta-miR-140 | CD86 | 15 | 18 | | bta-miR-497 | CD86 | 11 | 12 | bta-miR-10171-3p | | CD86 | 16 | 21 |
| bta-miR-181b | CD86 | 17 | 22 | | bta-miR-124b | CD86 | 11 | 12 | bta-miR-10179-5p | | CD86 | 16 | 21 |
| bta-miR-193a-3p | CD86 | 18 | 26 | | bta-miR-144 | CD86 | 13 | 14 | bta-miR-10180-3p | | CD86 | 15 | 20 |
| bta-miR-345-5p | CD86 | 19 | 21 | | bta-miR-182 | CD86 | 16 | 19 | bta-miR-10181-5p | | CD86 | 17 | 21 |
| bta-miR-210 | CD86 | 17 | 22 | | bta-miR-185 | CD86 | 17 | 25 | bta-miR-11971 | | CD86 | 14 | 16 |
| bta-miR-22-5p | CD86 | 13 | 17 | | bta-miR-299 | CD86 | 17 | 23 | bta-miR-2285bb | | CD86 | 22 | 41 |
| bta-miR-23a | CD86 | 14 | 18 | | bta-miR-329b | CD86 | 14 | 17 | bta-miR-12001 | | CD86 | 17 | 22 |
| bta-miR-425-5p | CD86 | 13 | 16 | | bta-miR-340 | CD86 | 12 | 15 | bta-miR-2299-3p | | CD86 | 16 | 19 |
| bta-miR-455-3p | CD86 | 11 | 14 | | bta-miR-362-5p | CD86 | 13 | 15 | bta-miR-2450d | | CD86 | 17 | 23 |
| bta-miR-150 | CD86 | 18 | 27 | | bta-miR-483 | CD86 | 15 | 20 | bta-miR-2285bb | | CD86 | 22 | 41 |
| bta-miR-532 | CD86 | 10 | 11 | | bta-miR-488 | CD86 | 14 | 15 | bta-miR-30b-3p | | IL-6 | 13 | 15 |
| bta-let-7e | CD86 | 13 | 15 | | bta-miR-500 | CD86 | 21 | 32 | bta-miR-423-5p | | IL-6 | 14 | 18 |
| bta-miR-143 | CD86 | 14 | 15 | | bta-miR-658 | CD86 | 20 | 25 | bta-miR-93 | | IL-6 | 12 | 16 |
| bta-miR-147 | CD86 | 16 | 21 | | bta-miR-760-3p | CD86 | 16 | 17 | bta-miR-365-5p | | IL-6 | 19 | 34 |
| bta-miR-181d | CD86 | 17 | 19 | | bta-miR-1307 | CD86 | 17 | 24 | bta-miR-296-3p | | IL-6 | 18 | 21 |
| bta-miR-182 | CD86 | 12 | 13 | | bta-miR-2286 | CD86 | 15 | 19 | bta-miR-671 | | IL-6 | 19 | 41 |
| bta-miR-183 | CD86 | 11 | 12 | | bta-miR-2287 | CD86 | 20 | 27 | bta-miR-2294 | | IL-6 | 13 | 19 |
| bta-miR-188 | CD86 | 15 | 21 | | bta-miR-2292 | CD86 | 18 | 20 | bta-miR-2324 | | IL-6 | 22 | 40 |
| bta-miR-193b | CD86 | 19 | 29 | | bta-miR-2295 | CD86 | 14 | 16 | bta-miR-2343 | | IL-6 | 14 | 16 |
| bta-miR-196b | CD86 | 23 | 27 | | bta-miR-2305 | CD86 | 15 | 20 | bta-miR-2348 | | IL-6 | 15 | 19 |
| bta-miR-216b | CD86 | 15 | 16 | | bta-miR-2309 | CD86 | 17 | 19 | bta-miR-2407 | | IL-6 | 17 | 23 |
| bta-miR-224 | CD86 | 14 | 16 | | bta-miR-2284l | CD86 | 13 | 14 | bta-miR-2407 | | IL-6 | 14 | 16 |
| bta-miR-28 | CD86 | 17 | 24 | | bta-miR-2284j | CD86 | 14 | 16 | bta-miR-2467-3p | | IL-6 | 15 | 19 |
| bta-miR-296-3p | CD86 | 16 | 21 | | bta-miR-2314 | CD86 | 15 | 19 | bta-miR-2467-3p | | IL-6 | 15 | 18 |
| bta-miR-328 | CD86 | 17 | 31 | | bta-miR-2284g | CD86 | 13 | 16 | bta-miR-2902 | | IL-6 | 20 | 28 |
| bta-miR-328 | CD86 | 19 | 50 | | bta-miR-2328-3p | CD86 | 20 | 23 | bta-miR-3141 | | IL-6 | 14 | 16 |
| bta-miR-339a | CD86 | 18 | 45 | | bta-miR-2330-5p | CD86 | 17 | 21 | bta-miR-6535 | | IL-6 | 19 | 25 |
| bta-miR-371 | CD86 | 15 | 18 | | bta-miR-2338 | CD86 | 18 | 33 | bta-miR-6535 | | IL-6 | 16 | 20 |
| bta-miR-448 | CD86 | 16 | 21 | | bta-miR-2343 | CD86 | 16 | 17 | bta-miR-7865 | | IL-6 | 15 | 20 |
| bta-miR-449b | CD86 | 15 | 22 | | bta-miR-2284f | CD86 | 11 | 12 | bta-miR-10171-3p | | IL-6 | 13 | 15 |
| bta-miR-449c | CD86 | 15 | 21 | | bta-miR-2368-5p | CD86 | 13 | 16 | bta-miR-2285ah-5p | | IL-6 | 16 | 19 |
| bta-miR-541 | CD86 | 13 | 17 | | bta-miR-2381 | CD86 | 16 | 25 | bta-miR-11971 | | IL-6 | 14 | 18 |
| bta-miR-671 | CD86 | 18 | 22 | | bta-miR-2392 | CD86 | 16 | 22 | bta-miR-140 | | IFNγ | 11 | 13 |
| bta-miR-744 | CD86 | 17 | 20 | | bta-miR-2392 | CD86 | 15 | 18 | bta-miR-196b | | IFNγ | 18 | 21 |
| bta-miR-940 | CD86 | 16 | 40 | | bta-miR-2397-3p | CD86 | 13 | 21 | bta-miR-296-3p | | IFNγ | 15 | 18 |
| bta-miR-1284 | CD86 | 16 | 22 | | bta-miR-2407 | CD86 | 18 | 24 | bta-miR-671 | | IFNγ | 20 | 24 |
| bta-miR-1291 | CD86 | 17 | 20 | | bta-miR-2407 | CD86 | 20 | 55 | bta-miR-2413 | | IFNγ | 16 | 22 |
| bta-miR-1835 | CD86 | 17 | 19 | | bta-miR-1584-5p | CD86 | 12 | 14 | bta-miR-320b | | IFNγ | 18 | 25 |
| bta-miR-1307 | CD86 | 16 | 21 | | bta-miR-2412 | CD86 | 20 | 28 | bta-miR-2887 | | IFNγ | 13 | 18 |
| bta-miR-1307 | CD86 | 18 | 34 | | bta-miR-2419-3p | CD86 | 17 | 19 | bta-miR-2897 | | IFNγ | 16 | 35 |
| bta-miR-2290 | CD86 | 15 | 19 | | bta-miR-2422 | CD86 | 14 | 16 | bta-miR-2902 | | IFNγ | 19 | 38 |
| bta-miR-2294 | CD86 | 18 | 24 | | bta-miR-2428 | CD86 | 18 | 24 | bta-miR-10175-5p | | IFNγ | 12 | 17 |
| bta-miR-2294 | CD86 | 16 | 19 | | bta-miR-2432 | CD86 | 17 | 22 | bta-miR-10179-5p | | IFNγ | 16 | 18 |
| bta-miR-2296 | CD86 | 17 | 21 | | bta-miR-2434 | CD86 | 16 | 18 | bta-miR-11972 | | IFNγ | 17 | 22 |
| bta-miR-2296 | CD86 | 14 | 20 | | bta-miR-2436-3p | CD86 | 13 | 17 | bta-miR-11989 | | IFNγ | 14 | 17 |
| bta-miR-2310 | CD86 | 15 | 18 | | bta-miR-2442 | CD86 | 16 | 17 | bta-miR-12015 | | IFNγ | 15 | 19 |
| bta-miR-2316 | CD86 | 15 | 17 | | bta-miR-2447 | CD86 | 17 | 25 | bta-miR-12026 | | IFNγ | 13 | 18 |
| bta-miR-1343-3p | CD86 | 15 | 19 | | bta-miR-2448-3p | CD86 | 17 | 24 | bta-miR-365-5p | | CD4 | 17 | 19 |
| bta-miR-2347 | CD86 | 11 | 12 | | bta-miR-2449 | CD86 | 18 | 23 | bta-miR-296-3p | | CD4 | 19 | 22 |
| bta-miR-2350 | CD86 | 16 | 22 | | bta-miR-2451 | CD86 | 16 | 19 | bta-miR-615 | | CD4 | 17 | 19 |
| bta-miR-2353 | CD86 | 15 | 19 | | bta-miR-1777a | CD86 | 17 | 24 | bta-miR-769 | | CD4 | 20 | 54 |
| bta-miR-2368-5p | CD86 | 15 | 21 | | bta-miR-2452 | CD86 | 19 | 22 | bta-miR-2296 | | CD4 | 15 | 19 |
| bta-miR-2368-5p | CD86 | 15 | 16 | | bta-miR-2470 | CD86 | 13 | 15 | bta-miR-2324 | | CD4 | 18 | 26 |
| bta-miR-2373-5p | CD86 | 19 | 28 | | bta-miR-2284o | CD86 | 13 | 16 | bta-miR-2328-3p | | CD4 | 16 | 20 |
| bta-miR-2378 | CD86 | 19 | 24 | | bta-miR-2284e | CD86 | 11 | 12 | bta-miR-2330-5p | | CD4 | 21 | 38 |
| bta-miR-2385-5p | CD86 | 16 | 19 | | bta-miR-2881 | CD86 | 16 | 20 | bta-miR-2338 | | CD4 | 20 | 25 |
| bta-miR-2388-5p | CD86 | 18 | 24 | | bta-miR-2885 | CD86 | 17 | 27 | bta-miR-2368-5p | | CD4 | 15 | 21 |
| bta-miR-2395 | CD86 | 16 | 18 | | bta-miR-2887 | CD86 | 16 | 22 | bta-miR-2374 | | CD4 | 18 | 30 |
| bta-miR-2399-5p | CD86 | 16 | 20 | | bta-miR-2888 | CD86 | 17 | 24 | bta-miR-2410 | | CD4 | 18 | 24 |
| bta-miR-2403 | CD86 | 13 | 16 | | bta-miR-2891 | CD86 | 13 | 14 | bta-miR-2433 | | CD4 | 21 | 34 |
| bta-miR-2412 | CD86 | 18 | 22 | | bta-miR-2897 | CD86 | 17 | 21 | bta-miR-2436-5p | | CD4 | 20 | 54 |
| bta-miR-2413 | CD86 | 21 | 29 | | bta-miR-2899 | CD86 | 14 | 16 | bta-miR-2450a | | CD4 | 16 | 19 |
| bta-miR-2418 | CD86 | 18 | 22 | | bta-miR-2900 | CD86 | 15 | 19 | bta-miR-6121-3p | | CD4 | 16 | 17 |
| bta-miR-1814c | CD86 | 15 | 17 | | bta-miR-2284w | CD86 | 14 | 15 | bta-miR-3154 | | CD4 | 16 | 20 |
| bta-miR-2422 | CD86 | 18 | 27 | | bta-miR-2284x | CD86 | 14 | 16 | bta-miR-154b | | CD4 | 17 | 20 |
| bta-miR-2422 | CD86 | 15 | 19 | | bta-miR-1260b | CD86 | 15 | 19 | bta-miR-2285q | | CD4 | 16 | 20 |
| bta-miR-2422 | CD86 | 13 | 17 | | bta-miR-2284y | CD86 | 14 | 16 | bta-miR-10175-5p | | CD4 | 16 | 18 |
| bta-miR-1843 | CD86 | 17 | 19 | | bta-miR-6519 | CD86 | 16 | 18 | bta-miR-2285ah-5p | | CD4 | 20 | 27 |
| bta-miR-2425-5p | CD86 | 11 | 15 | | bta-miR-3956 | CD86 | 16 | 21 | bta-miR-12028 | | CD4 | 16 | 20 |
| bta-miR-2426 | CD86 | 13 | 15 | | bta-miR-6525 | CD86 | 14 | 20 | bta-miR-12061 | | CD4 | 15 | 17 |
| bta-miR-2436-3p | CD86 | 12 | 16 | | bta-miR-6528 | CD86 | 16 | 19 | bta-miR-331-3p | | CD4 | 12 | 13 |
| bta-miR-2438 | CD86 | 18 | 23 | | bta-miR-7865 | CD86 | 17 | 28 | bta-miR-329b | | CD4 | 16 | 18 |
| bta-miR-2439-5p | CD86 | 17 | 22 | | bta-miR-7865 | CD86 | 15 | 17 | bta-miR-154b | | CD4 | 17 | 20 |
| bta-miR-2449 | CD86 | 15 | 18 | | bta-miR-8550 | CD86 | 15 | 19 | bta-miR-27a-5p | | CXCL8 | 18 | 27 |
| bta-miR-2450a | CD86 | 15 | 17 | | bta-miR-1842 | CD86 | 18 | 23 | bta-miR-2360 | | CXCL8 | 19 | 27 |
| bta-miR-1777a | CD86 | 17 | 30 | | bta-miR-4523 | CD86 | 14 | 16 | bta-miR-2364 | | CXCL8 | 18 | 25 |
| bta-miR-2461-5p | CD86 | 17 | 28 | | bta-miR-10164-3p | CD86 | 21 | 29 | bta-miR-2401 | | CXCL8 | 14 | 18 |
| bta-miR-2464-3p | CD86 | 17 | 21 | | bta-miR-10167-3p | CD86 | 19 | 30 | bta-miR-2457 | | CXCL8 | 19 | 26 |
| bta-miR-2466-5p | CD86 | 17 | 23 | | bta-miR-10171-3p | CD86 | 16 | 19 | bta-miR-503-3p | | CXCL8 | 19 | 27 |
| bta-miR-2467-5p | CD86 | 18 | 27 | | bta-miR-10172-3p | CD86 | 14 | 17 | bta-miR-12004 | | CXCL8 | 19 | 39 |
| bta-miR-2467-3p | CD86 | 19 | 25 | | bta-miR-10174-5p | CD86 | 15 | 20 | bta-miR-484 | | ICAM1 | 18 | 40 |
| bta-miR-2467-3p | CD86 | 16 | 21 | | bta-miR-10177-5p | CD86 | 12 | 14 | bta-miR-30b-3p | | ICAM1 | 13 | 16 |
| bta-miR-2467-3p | CD86 | 21 | 51 | | bta-miR-2285ah-5p | CD86 | 17 | 26 | bta-miR-193a-5p | | ICAM1 | 16 | 18 |
| bta-miR-2471-3p | CD86 | 19 | 32 | | bta-miR-2285aj-5p | CD86 | 14 | 16 | bta-miR-200c | | ICAM1 | 19 | 23 |
| bta-miR-2472 | CD86 | 19 | 27 | | bta-miR-2285ak-5p | CD86 | 14 | 15 | bta-miR-423-5p | | ICAM1 | 16 | 19 |
| bta-miR-2481 | CD86 | 15 | 16 | | bta-miR-2285al-5p | CD86 | 16 | 20 | bta-miR-296-5p | | ICAM1 | 14 | 17 |
| bta-miR-2483-5p | CD86 | 11 | 13 | | bta-miR-11971 | CD86 | 14 | 17 | bta-miR-370 | | ICAM1 | 19 | 41 |
| bta-miR-2483-3p | CD86 | 16 | 18 | | bta-miR-11972 | CD86 | 15 | 16 | bta-miR-452 | | ICAM1 | 18 | 23 |
| bta-miR-2486-5p | CD86 | 18 | 20 | | bta-miR-2285au | CD86 | 14 | 16 | bta-miR-483 | | ICAM1 | 18 | 22 |
| bta-miR-1388-5p | CD86 | 13 | 16 | | bta-miR-2285av | CD86 | 14 | 16 | bta-miR-940 | | ICAM1 | 16 | 17 |
| bta-miR-1388-3p | CD86 | 19 | 42 | | bta-miR-11976 | CD86 | 17 | 22 | bta-miR-1224 | | ICAM1 | 17 | 22 |
| bta-miR-1468 | CD86 | 17 | 20 | | bta-miR-11979 | CD86 | 18 | 24 | bta-miR-1249 | | ICAM1 | 17 | 22 |
| bta-miR-2882 | CD86 | 17 | 26 | | bta-miR-2285bh | CD86 | 14 | 16 | bta-miR-2288 | | ICAM1 | 17 | 18 |
| bta-miR-2894 | CD86 | 14 | 19 | | bta-miR-11989 | CD86 | 14 | 17 | bta-miR-2290 | | ICAM1 | 17 | 20 |
| bta-miR-2895 | CD86 | 15 | 19 | | bta-miR-2285bz | CD86 | 14 | 16 | bta-miR-2295 | | ICAM1 | 16 | 19 |
| bta-miR-2902 | CD86 | 18 | 23 | | bta-miR-11996 | CD86 | 18 | 48 | bta-miR-2308 | | ICAM1 | 15 | 21 |
| bta-miR-2904 | CD86 | 18 | 39 | | bta-miR-2285ca | CD86 | 11 | 15 | bta-miR-2324 | | ICAM1 | 20 | 23 |
| bta-miR-3141 | CD86 | 15 | 19 | | bta-miR-2285cp | CD86 | 14 | 16 | bta-miR-2338 | | ICAM1 | 17 | 20 |
| bta-miR-6121-5p | CD86 | 16 | 17 | | bta-miR-2285cr | CD86 | 14 | 15 | bta-miR-2353 | | ICAM1 | 18 | 28 |
| bta-miR-6517 | CD86 | 18 | 21 | | bta-miR-2285db | CD86 | 11 | 12 | bta-miR-2354 | | ICAM1 | 14 | 16 |
| bta-miR-6518 | CD86 | 16 | 22 | | bta-miR-12031 | CD86 | 11 | 14 | bta-miR-2356 | | ICAM1 | 20 | 24 |
| bta-miR-378c | CD86 | 14 | 16 | | bta-miR-2285dj | CD86 | 14 | 16 | bta-miR-2373-3p | | ICAM1 | 15 | 17 |
| bta-miR-378c | CD86 | 15 | 17 | | bta-miR-12048 | CD86 | 16 | 18 | bta-miR-2415-5p | | ICAM1 | 19 | 32 |
| bta-miR-6525 | CD86 | 13 | 14 | | bta-miR-12056 | CD86 | 16 | 20 | bta-miR-2422 | | ICAM1 | 15 | 20 |
| bta-miR-6528 | CD86 | 17 | 27 | | bta-miR-12057 | CD86 | 17 | 22 | bta-miR-339b | | ICAM1 | 17 | 32 |
| bta-miR-6529a | CD86 | 20 | 26 | | bta-miR-103 | CD86 | 15 | 17 | bta-miR-2472 | | ICAM1 | 17 | 21 |
| bta-miR-664b | CD86 | 16 | 24 | | bta-miR-107 | CD86 | 15 | 18 | bta-miR-1468 | | ICAM1 | 15 | 19 |
| bta-miR-7861 | CD86 | 17 | 24 | | bta-miR-15b | CD86 | 17 | 21 | bta-miR-3600 | | ICAM1 | 14 | 17 |
| bta-miR-7862 | CD86 | 17 | 30 | | bta-let-7d | CD86 | 13 | 18 | bta-miR-3602 | | ICAM1 | 17 | 22 |
| bta-miR-7864 | CD86 | 19 | 30 | | bta-miR-124a | CD86 | 11 | 12 | bta-miR-6520 | | ICAM1 | 15 | 17 |
| bta-miR-7864 | CD86 | 15 | 19 | | bta-miR-148b | CD86 | 19 | 25 | bta-miR-10165-5p | | ICAM1 | 16 | 19 |
| bta-miR-7865 | CD86 | 16 | 19 | | bta-miR-93 | CD86 | 20 | 26 | bta-miR-11971 | | ICAM1 | 16 | 20 |
| bta-miR-8550 | CD86 | 13 | 18 | | bta-miR-23b-5p | CD86 | 14 | 19 | bta-miR-12028 | | ICAM1 | 13 | 14 |
| bta-miR-1842 | CD86 | 16 | 21 | | bta-miR-497 | CD86 | 11 | 13 | bta-miR-12054 | | ICAM1 | 13 | 17 |
| bta-miR-10163-3p | CD86 | 18 | 21 | | bta-miR-124b | CD86 | 11 | 12 | bta-miR-31 | | IL-4 | 16 | 19 |
| bta-miR-10164-3p | CD86 | 19 | 22 | | bta-miR-136 | CD86 | 15 | 16 | bta-miR-106b | | IL-4 | 14 | 16 |
| bta-miR-10169-3p | CD86 | 13 | 15 | | bta-miR-144 | CD86 | 13 | 14 | bta-miR-2897 | | IL-4 | 13 | 17 |
| bta-miR-7857-3p | CD86 | 16 | 20 | | bta-miR-182 | CD86 | 16 | 19 | bta-miR-148d | | IL-4 | 17 | 22 |
| bta-miR-10175-3p | CD86 | 18 | 22 | | bta-miR-185 | CD86 | 17 | 25 | bta-miR-12026 | | IL-4 | 14 | 16 |
| bta-miR-10175-3p | CD86 | 18 | 20 | | bta-miR-296-3p | CD86 | 18 | 20 | bta-miR-221 | | CSF2 | 15 | 20 |
| bta-miR-10178-5p | CD86 | 16 | 20 | | bta-miR-299 | CD86 | 17 | 23 | bta-miR-1301 | | CSF2 | 17 | 25 |
| bta-miR-668-3p | CD86 | 8 | 10 | | bta-miR-329b | CD86 | 14 | 17 | bta-miR-2289 | | CSF2 | 17 | 35 |
| bta-miR-10183-5p | CD86 | 17 | 22 | | bta-miR-340 | CD86 | 12 | 15 | bta-miR-2455 | | CSF2 | 17 | 20 |
| bta-miR-11974 | CD86 | 19 | 31 | | bta-miR-362-5p | CD86 | 18 | 23 | bta-miR-2486-5p | | CSF2 | 23 | 39 |
| bta-miR-11983 | CD86 | 17 | 27 | | bta-miR-483 | CD86 | 15 | 20 | bta-miR-132 | | CD14 | 17 | 19 |
| bta-miR-7180 | CD86 | 23 | 34 | | bta-miR-488 | CD86 | 14 | 15 | bta-miR-219-3p | | CD14 | 12 | 14 |
| bta-miR-2285bz | CD86 | 13 | 15 | | bta-miR-500 | CD86 | 21 | 32 | bta-miR-767 | | CD14 | 17 | 27 |
| bta-miR-11995 | CD86 | 18 | 23 | | bta-miR-502a | CD86 | 16 | 19 | bta-miR-1296 | | CD14 | 18 | 23 |
| bta-miR-2285cb | CD86 | 12 | 15 | | bta-miR-658 | CD86 | 20 | 25 | bta-miR-1287 | | CD14 | 20 | 40 |
| bta-miR-11997 | CD86 | 12 | 16 | | bta-miR-760-3p | CD86 | 16 | 17 | bta-miR-2304 | | CD14 | 16 | 20 |
| bta-miR-12001 | CD86 | 20 | 25 | | bta-miR-763 | CD86 | 17 | 22 | bta-miR-2321 | | CD14 | 10 | 11 |
| bta-miR-6775 | CD86 | 23 | 60 | | bta-miR-1307 | CD86 | 17 | 24 | bta-miR-2329-3p | | CD14 | 14 | 18 |
| bta-miR-12013 | CD86 | 17 | 26 | | bta-miR-2286 | CD86 | 15 | 19 | bta-miR-2331-5p | | CD14 | 20 | 32 |
| bta-miR-12014 | CD86 | 19 | 22 | | bta-miR-2287 | CD86 | 20 | 27 | bta-miR-2387 | | CD14 | 17 | 22 |
| bta-miR-12025 | CD86 | 17 | 23 | | bta-miR-2292 | CD86 | 18 | 20 | bta-miR-677 | | CD14 | 16 | 20 |
| bta-miR-12028 | CD86 | 13 | 17 | | bta-miR-2294 | CD86 | 18 | 23 | bta-miR-149-5p | | CD14 | 20 | 34 |
| bta-miR-12031 | CD86 | 14 | 18 | | bta-miR-2295 | CD86 | 14 | 16 | bta-miR-6120-5p | | CD14 | 13 | 15 |
| bta-miR-12033 | CD86 | 13 | 16 | | bta-miR-2305 | CD86 | 15 | 20 | bta-miR-6531 | | CD14 | 15 | 18 |
| bta-miR-12045 | CD86 | 12 | 20 | | bta-miR-2309 | CD86 | 17 | 19 | bta-miR-6529b | | CD14 | 15 | 20 |
| bta-miR-507b | CD86 | 16 | 18 | | bta-miR-2284l | CD86 | 13 | 14 | bta-miR-6529b | | CD14 | 14 | 18 |
| bta-miR-12050 | CD86 | 15 | 17 | | bta-miR-2284j | CD86 | 14 | 16 | bta-miR-7859 | | CD14 | 16 | 27 |
| bta-miR-12053 | CD86 | 17 | 27 | | bta-miR-2314 | CD86 | 15 | 19 | bta-miR-668-3p | | CD14 | 14 | 15 |
| bta-miR-12054 | CD86 | 13 | 18 | | bta-miR-2284g | CD86 | 13 | 16 | bta-miR-7180 | | CD14 | 22 | 29 |
| bta-miR-12057 | CD86 | 15 | 18 | | bta-miR-2328-3p | CD86 | 20 | 23 | bta-miR-12029 | | CD14 | 19 | 22 |
| bta-miR-12061 | CD86 | 15 | 18 | | bta-miR-2328-3p | CD86 | 18 | 19 | bta-miR-12039 | | CD14 | 19 | 23 |
| bta-miR-221 | CD86 | 19 | 20 | | bta-miR-2330-5p | CD86 | 17 | 21 | bta-miR-12050 | | CD14 | 19 | 37 |
| bta-miR-27a-5p | CD86 | 19 | 27 | | bta-miR-2338 | CD86 | 18 | 33 | bta-miR-12059 | | CD14 | 20 | 34 |
| bta-miR-27a-3p | CD86 | 12 | 13 | | bta-miR-2284f | CD86 | 11 | 12 | bta-miR-21-3p | | TLR4 | 12 | 16 |
| bta-miR-484 | CD86 | 18 | 26 | | bta-miR-2368-5p | CD86 | 13 | 16 | bta-miR-345-3p | | TLR4 | 17 | 21 |
| bta-miR-484 | CD86 | 15 | 18 | | bta-miR-2381 | CD86 | 16 | 25 | bta-miR-423-5p | | TLR4 | 12 | 17 |
| bta-miR-128 | CD86 | 16 | 23 | | bta-miR-2388-3p | CD86 | 15 | 20 | bta-miR-34a | | TLR4 | 16 | 19 |
| bta-miR-199a-5p | CD86 | 17 | 22 | | bta-miR-2389 | CD86 | 18 | 19 | bta-miR-134 | | TLR4 | 16 | 21 |
| bta-miR-27b | CD86 | 12 | 13 | | bta-miR-2392 | CD86 | 16 | 22 | bta-miR-187 | | TLR4 | 19 | 22 |
| bta-miR-31 | CD86 | 15 | 25 | | bta-miR-2392 | CD86 | 15 | 18 | bta-miR-217 | | TLR4 | 18 | 20 |
| bta-miR-140 | CD86 | 15 | 18 | | bta-miR-2407 | CD86 | 18 | 24 | bta-miR-500 | | TLR4 | 17 | 22 |
| bta-miR-181b | CD86 | 17 | 22 | | bta-miR-2407 | CD86 | 20 | 55 | bta-miR-504 | | TLR4 | 17 | 34 |
| bta-miR-193a-3p | CD86 | 18 | 26 | | bta-miR-1584-5p | CD86 | 12 | 14 | bta-miR-671 | | TLR4 | 21 | 30 |
| bta-miR-345-5p | CD86 | 19 | 21 | | bta-miR-2412 | CD86 | 20 | 28 | bta-miR-744 | | TLR4 | 19 | 28 |
| bta-miR-210 | CD86 | 17 | 22 | | bta-miR-2415-5p | CD86 | 16 | 23 | bta-miR-744 | | TLR4 | 12 | 14 |
| bta-miR-22-5p | CD86 | 13 | 17 | | bta-miR-2422 | CD86 | 16 | 19 | bta-miR-1224 | | TLR4 | 17 | 23 |
| bta-miR-23a | CD86 | 14 | 18 | | bta-miR-2428 | CD86 | 18 | 24 | bta-miR-2287 | | TLR4 | 18 | 23 |
| bta-miR-425-5p | CD86 | 13 | 16 | | bta-miR-2432 | CD86 | 17 | 22 | bta-miR-2299-5p | | TLR4 | 17 | 21 |
| bta-miR-455-3p | CD86 | 11 | 14 | | bta-miR-2434 | CD86 | 16 | 18 | bta-miR-2328-3p | | TLR4 | 16 | 20 |
| bta-miR-150 | CD86 | 18 | 27 | | bta-miR-2436-3p | CD86 | 13 | 17 | bta-miR-2334 | | TLR4 | 12 | 13 |
| bta-miR-532 | CD86 | 10 | 11 | | bta-miR-2442 | CD86 | 16 | 17 | bta-miR-2338 | | TLR4 | 18 | 22 |
| bta-let-7e | CD86 | 13 | 15 | | bta-miR-2447 | CD86 | 17 | 25 | bta-miR-1814a | | TLR4 | 18 | 20 |
| bta-miR-143 | CD86 | 14 | 15 | | bta-miR-2448-3p | CD86 | 17 | 24 | bta-miR-2356 | | TLR4 | 18 | 21 |
| bta-miR-147 | CD86 | 16 | 21 | | bta-miR-2451 | CD86 | 16 | 19 | bta-miR-2368-5p | | TLR4 | 17 | 22 |
| bta-miR-181d | CD86 | 17 | 19 | | bta-miR-1777a | CD86 | 17 | 24 | bta-miR-2374 | | TLR4 | 17 | 27 |
| bta-miR-182 | CD86 | 12 | 13 | | bta-miR-2452 | CD86 | 19 | 22 | bta-miR-2381 | | TLR4 | 15 | 16 |
| bta-miR-183 | CD86 | 11 | 12 | | bta-miR-2454-5p | CD86 | 15 | 22 | bta-miR-2382-3p | | TLR4 | 21 | 36 |
| bta-miR-193b | CD86 | 19 | 29 | | bta-miR-2470 | CD86 | 13 | 15 | bta-miR-2398 | | TLR4 | 15 | 17 |
| bta-miR-196b | CD86 | 23 | 27 | | bta-miR-2284o | CD86 | 13 | 16 | bta-miR-2403 | | TLR4 | 19 | 22 |
| bta-miR-216b | CD86 | 15 | 16 | | bta-miR-2284e | CD86 | 11 | 12 | bta-miR-2406 | | TLR4 | 17 | 32 |
| bta-miR-224 | CD86 | 14 | 16 | | bta-miR-2881 | CD86 | 16 | 20 | bta-miR-2412 | | TLR4 | 19 | 25 |
| bta-miR-28 | CD86 | 17 | 24 | | bta-miR-2887 | CD86 | 16 | 21 | bta-miR-2413 | | TLR4 | 14 | 19 |
| bta-miR-296-3p | CD86 | 16 | 21 | | bta-miR-2887 | CD86 | 16 | 22 | bta-miR-2428 | | TLR4 | 19 | 22 |
| bta-miR-328 | CD86 | 17 | 31 | | bta-miR-2888 | CD86 | 17 | 24 | bta-miR-2433 | | TLR4 | 18 | 42 |
| bta-miR-328 | CD86 | 19 | 50 | | bta-miR-2891 | CD86 | 13 | 14 | bta-miR-2447 | | TLR4 | 19 | 42 |
| bta-miR-339a | CD86 | 18 | 45 | | bta-miR-2899 | CD86 | 14 | 16 | bta-miR-2450a | | TLR4 | 18 | 23 |
| bta-miR-371 | CD86 | 15 | 18 | | bta-miR-2900 | CD86 | 15 | 19 | bta-miR-2453 | | TLR4 | 17 | 33 |
| bta-miR-448 | CD86 | 16 | 21 | | bta-miR-2284w | CD86 | 14 | 15 | bta-miR-2454-3p | | TLR4 | 14 | 15 |
| bta-miR-449b | CD86 | 15 | 22 | | bta-miR-2284x | CD86 | 14 | 16 | bta-miR-2455 | | TLR4 | 17 | 20 |
| bta-miR-449c | CD86 | 15 | 21 | | bta-miR-1260b | CD86 | 15 | 19 | bta-miR-2456 | | TLR4 | 17 | 23 |
| bta-miR-541 | CD86 | 13 | 17 | | bta-miR-2284y | CD86 | 14 | 16 | bta-miR-2461-5p | | TLR4 | 19 | 26 |
| bta-miR-671 | CD86 | 18 | 22 | | bta-miR-3154 | CD86 | 15 | 19 | bta-miR-2461-3p | | TLR4 | 17 | 23 |
| bta-miR-744 | CD86 | 17 | 20 | | bta-miR-3956 | CD86 | 16 | 21 | bta-miR-2467-3p | | TLR4 | 19 | 35 |
| bta-miR-940 | CD86 | 16 | 40 | | bta-miR-378c | CD86 | 15 | 17 | bta-miR-664a | | TLR4 | 18 | 27 |
| bta-miR-1284 | CD86 | 16 | 22 | | bta-miR-6528 | CD86 | 16 | 19 | bta-miR-2899 | | TLR4 | 16 | 18 |
| bta-miR-1291 | CD86 | 17 | 20 | | bta-miR-6516 | CD86 | 15 | 20 | bta-miR-2902 | | TLR4 | 18 | 22 |
| bta-miR-1307 | CD86 | 16 | 21 | | bta-miR-7865 | CD86 | 17 | 28 | bta-miR-149-3p | | TLR4 | 19 | 38 |
| bta-miR-1307 | CD86 | 18 | 34 | | bta-miR-8550 | CD86 | 16 | 20 | bta-miR-6535 | | TLR4 | 16 | 17 |
| bta-miR-2286 | CD86 | 12 | 14 | | bta-miR-8550 | CD86 | 15 | 19 | bta-miR-6535 | | TLR4 | 17 | 26 |
| bta-miR-2290 | CD86 | 15 | 19 | | bta-miR-1842 | CD86 | 18 | 23 | bta-miR-7862 | | TLR4 | 16 | 23 |
| bta-miR-2294 | CD86 | 18 | 24 | | bta-miR-4523 | CD86 | 14 | 16 | bta-miR-1842 | | TLR4 | 19 | 40 |
| bta-miR-2294 | CD86 | 16 | 19 | | bta-miR-10164-3p | CD86 | 21 | 29 | bta-miR-4449 | | TLR4 | 21 | 30 |
| bta-miR-2296 | CD86 | 17 | 21 | | bta-miR-10167-3p | CD86 | 17 | 18 | bta-miR-10173-5p | | TLR4 | 17 | 21 |
| bta-miR-2296 | CD86 | 14 | 20 | | bta-miR-10172-3p | CD86 | 14 | 17 | bta-miR-668-3p | | TLR4 | 16 | 25 |
| bta-miR-2310 | CD86 | 15 | 18 | | bta-miR-10174-5p | CD86 | 15 | 20 | bta-miR-11972 | | TLR4 | 17 | 22 |
| bta-miR-2316 | CD86 | 15 | 17 | | bta-miR-10176-5p | CD86 | 13 | 16 | bta-miR-11981 | | TLR4 | 18 | 24 |
| bta-miR-1343-5p | CD86 | 18 | 25 | | bta-miR-10177-5p | CD86 | 12 | 14 | bta-miR-11984 | | TLR4 | 14 | 19 |
| bta-miR-1343-3p | CD86 | 15 | 19 | | bta-miR-2285ah-5p | CD86 | 17 | 26 | bta-miR-7180 | | TLR4 | 21 | 37 |
| bta-miR-2347 | CD86 | 11 | 12 | | bta-miR-2285aj-5p | CD86 | 14 | 16 | bta-miR-7180 | | TLR4 | 21 | 26 |
| bta-miR-2348 | CD86 | 16 | 20 | | bta-miR-2285ak-5p | CD86 | 14 | 16 | bta-miR-11992 | | TLR4 | 12 | 14 |
| bta-miR-2350 | CD86 | 16 | 23 | | bta-miR-2285al-5p | CD86 | 16 | 20 | bta-miR-12001 | | TLR4 | 15 | 19 |
| bta-miR-2353 | CD86 | 15 | 19 | | bta-miR-11971 | CD86 | 14 | 18 | bta-miR-12004 | | TLR4 | 21 | 51 |
| bta-miR-2368-5p | CD86 | 15 | 21 | | bta-miR-11972 | CD86 | 17 | 25 | bta-miR-12014 | | TLR4 | 16 | 18 |
| bta-miR-2368-5p | CD86 | 15 | 16 | | bta-miR-2285au | CD86 | 14 | 16 | bta-miR-12026 | | TLR4 | 15 | 16 |
| bta-miR-2373-5p | CD86 | 19 | 28 | | bta-miR-2285av | CD86 | 14 | 16 | bta-miR-12027 | | TLR4 | 19 | 37 |
| bta-miR-2378 | CD86 | 19 | 24 | | bta-miR-11976 | CD86 | 17 | 22 | bta-miR-12029 | | TLR4 | 16 | 22 |
| bta-miR-2385-5p | CD86 | 16 | 19 | | bta-miR-11979 | CD86 | 18 | 24 | bta-miR-12042 | | TLR4 | 21 | 36 |
| bta-miR-2388-5p | CD86 | 18 | 24 | | bta-miR-2285bh | CD86 | 14 | 16 | bta-miR-12049 | | TLR4 | 18 | 38 |
| bta-miR-2395 | CD86 | 16 | 18 | | bta-miR-11989 | CD86 | 14 | 17 | bta-miR-12053 | | TLR4 | 18 | 25 |
| bta-miR-2397-3p | CD86 | 12 | 16 | | bta-miR-2285bz | CD86 | 14 | 16 | bta-miR-12061 | | TLR4 | 18 | 23 |
| bta-miR-2399-5p | CD86 | 16 | 20 | | bta-miR-11996 | CD86 | 18 | 48 | bta-miR-21-3p | | IL-18 | 16 | 22 |
| bta-miR-2403 | CD86 | 13 | 16 | | bta-miR-2285ca | CD86 | 11 | 15 | bta-miR-34a | | IL-18 | 17 | 21 |
| bta-miR-2412 | CD86 | 18 | 22 | | bta-miR-2285cp | CD86 | 14 | 16 | bta-miR-106b | | IL-18 | 16 | 26 |
| bta-miR-2413 | CD86 | 21 | 29 | | bta-miR-2285cr | CD86 | 14 | 15 | bta-miR-133a | | IL-18 | 16 | 21 |
| bta-miR-2418 | CD86 | 18 | 22 | | bta-miR-2285db | CD86 | 11 | 12 | bta-miR-133b | | IL-18 | 16 | 21 |
| bta-miR-1814c | CD86 | 15 | 17 | | bta-miR-12031 | CD86 | 11 | 14 | bta-miR-185 | | IL-18 | 16 | 22 |
| bta-miR-2422 | CD86 | 18 | 27 | | bta-miR-2285dj | CD86 | 14 | 16 | bta-miR-196b | | IL-18 | 19 | 37 |
| bta-miR-2422 | CD86 | 15 | 19 | | bta-miR-12048 | CD86 | 16 | 18 | bta-miR-486 | | IL-18 | 16 | 25 |
| bta-miR-2422 | CD86 | 13 | 17 | | bta-miR-12056 | CD86 | 16 | 20 | bta-miR-615 | | IL-18 | 16 | 39 |
| bta-miR-1843 | CD86 | 17 | 19 | | bta-miR-199a-3p | IL-6 | 10 | 11 | bta-miR-658 | | IL-18 | 21 | 33 |
| bta-miR-2425-5p | CD86 | 11 | 15 | | bta-miR-17-3p | IL-6 | 16 | 19 | bta-miR-744 | | IL-18 | 13 | 18 |
| bta-miR-2426 | CD86 | 13 | 15 | | bta-miR-23b-5p | IL-6 | 20 | 25 | bta-miR-877 | | IL-18 | 14 | 17 |
| bta-miR-2436-3p | CD86 | 12 | 16 | | bta-miR-34a | IL-6 | 17 | 24 | bta-miR-2331-3p | | IL-18 | 12 | 20 |
| bta-miR-2438 | CD86 | 18 | 23 | | bta-miR-134 | IL-6 | 16 | 20 | bta-miR-2366 | | IL-18 | 16 | 33 |
| bta-miR-2439-5p | CD86 | 17 | 22 | | bta-miR-28 | IL-6 | 15 | 17 | bta-miR-2392 | | IL-18 | 13 | 16 |
| bta-miR-2449 | CD86 | 15 | 18 | | bta-miR-378 | IL-6 | 16 | 21 | bta-miR-2399-5p | | IL-18 | 13 | 15 |
| bta-miR-2450a | CD86 | 15 | 17 | | bta-miR-449a | IL-6 | 17 | 20 | bta-miR-2422 | | IL-18 | 14 | 19 |
| bta-miR-1777a | CD86 | 17 | 30 | | bta-miR-760-3p | IL-6 | 15 | 17 | bta-miR-2436-5p | | IL-18 | 19 | 42 |
| bta-miR-2461-5p | CD86 | 17 | 28 | | bta-miR-760-3p | IL-6 | 13 | 16 | bta-miR-2450a | | IL-18 | 18 | 23 |
| bta-miR-2464-3p | CD86 | 17 | 21 | | bta-miR-1301 | IL-6 | 16 | 23 | bta-miR-2467-3p | | IL-18 | 17 | 22 |
| bta-miR-2466-5p | CD86 | 17 | 23 | | bta-miR-1287 | IL-6 | 16 | 19 | bta-miR-2899 | | IL-18 | 17 | 22 |
| bta-miR-2466-3p | CD86 | 13 | 17 | | bta-miR-2295 | IL-6 | 16 | 18 | bta-miR-2900 | | IL-18 | 14 | 18 |
| bta-miR-2467-5p | CD86 | 18 | 27 | | bta-miR-2299-3p | IL-6 | 15 | 16 | bta-miR-6519 | | IL-18 | 14 | 17 |
| bta-miR-2467-3p | CD86 | 19 | 25 | | bta-miR-2302 | IL-6 | 12 | 16 | bta-miR-503-5p | | IL-18 | 14 | 17 |
| bta-miR-2467-3p | CD86 | 16 | 21 | | bta-miR-2305 | IL-6 | 19 | 24 | bta-miR-6534 | | IL-18 | 14 | 17 |
| bta-miR-2467-3p | CD86 | 21 | 51 | | bta-miR-2316 | IL-6 | 18 | 22 | bta-miR-2450d | | IL-18 | 20 | 35 |
| bta-miR-2471-3p | CD86 | 19 | 32 | | bta-miR-2330-3p | IL-6 | 13 | 18 | bta-miR-10167-3p | | IL-18 | 15 | 18 |
| bta-miR-2472 | CD86 | 19 | 27 | | bta-miR-199c | IL-6 | 10 | 11 | bta-miR-323b-3p | | IL-18 | 16 | 20 |
| bta-miR-2481 | CD86 | 15 | 16 | | bta-miR-2338 | IL-6 | 14 | 18 | bta-miR-10185-5p | | IL-18 | 19 | 22 |
| bta-miR-2483-5p | CD86 | 11 | 13 | | bta-miR-2355-3p | IL-6 | 13 | 16 | bta-miR-2285ah-5p | | IL-18 | 19 | 22 |
| bta-miR-2483-3p | CD86 | 16 | 18 | | bta-miR-2364 | IL-6 | 15 | 18 | bta-miR-11981 | | IL-18 | 21 | 27 |
| bta-miR-2486-5p | CD86 | 18 | 20 | | bta-miR-2374 | IL-6 | 19 | 21 | bta-miR-12051 | | IL-18 | 11 | 13 |
| bta-miR-1388-5p | CD86 | 13 | 16 | | bta-miR-2381 | IL-6 | 11 | 15 | bta-miR-12057 | | IL-18 | 15 | 18 |
| bta-miR-1388-3p | CD86 | 19 | 42 | | bta-miR-2382-5p | IL-6 | 19 | 21 | bta-miR-375 | | TLR2 | 18 | 23 |
| bta-miR-1468 | CD86 | 17 | 20 | | bta-miR-2389 | IL-6 | 18 | 22 | bta-miR-1256 | | TLR2 | 17 | 32 |
| bta-miR-2894 | CD86 | 14 | 19 | | bta-miR-2392 | IL-6 | 15 | 18 | bta-miR-2301 | | TLR2 | 20 | 34 |
| bta-miR-2895 | CD86 | 15 | 19 | | bta-miR-2396 | IL-6 | 12 | 14 | bta-miR-2309 | | TLR2 | 17 | 18 |
| bta-miR-2902 | CD86 | 18 | 23 | | bta-miR-1584-5p | IL-6 | 16 | 19 | bta-miR-2357 | | TLR2 | 20 | 34 |
| bta-miR-2904 | CD86 | 18 | 39 | | bta-miR-2411-5p | IL-6 | 16 | 19 | bta-miR-2392 | | TLR2 | 17 | 22 |
| bta-miR-3141 | CD86 | 15 | 19 | | bta-miR-2412 | IL-6 | 19 | 24 | bta-miR-2428 | | TLR2 | 16 | 22 |
| bta-miR-6121-5p | CD86 | 16 | 17 | | bta-miR-2413 | IL-6 | 18 | 22 | bta-miR-2438 | | TLR2 | 22 | 50 |
| bta-miR-6517 | CD86 | 18 | 21 | | bta-miR-2415-3p | IL-6 | 12 | 18 | bta-miR-2486-5p | | TLR2 | 20 | 21 |
| bta-miR-6518 | CD86 | 16 | 22 | | bta-miR-2426 | IL-6 | 15 | 20 | bta-miR-3604 | | TLR2 | 14 | 16 |
| bta-miR-378c | CD86 | 14 | 16 | | bta-miR-2428 | IL-6 | 16 | 18 | bta-miR-7862 | | TLR2 | 17 | 26 |
| bta-miR-6525 | CD86 | 13 | 14 | | bta-miR-2431-5p | IL-6 | 12 | 18 | bta-miR-219 | | TLR2 | 14 | 18 |
| bta-miR-6528 | CD86 | 17 | 27 | | bta-miR-2448-3p | IL-6 | 18 | 20 | bta-miR-8549 | | TLR2 | 13 | 14 |
| bta-miR-6529a | CD86 | 20 | 26 | | bta-miR-1777a | IL-6 | 19 | 26 | bta-miR-375 | | TLR2 | 15 | 19 |
| bta-miR-664b | CD86 | 16 | 24 | | bta-miR-2453 | IL-6 | 19 | 34 | bta-miR-2348 | | CCL2 | 16 | 28 |
| bta-miR-7861 | CD86 | 17 | 24 | | bta-miR-2455 | IL-6 | 17 | 19 | bta-miR-2385-5p | | CCL2 | 16 | 19 |
| bta-miR-7862 | CD86 | 17 | 30 | | bta-miR-2459 | IL-6 | 13 | 14 | bta-miR-2440 | | CCL2 | 11 | 13 |
| bta-miR-7864 | CD86 | 19 | 30 | | bta-miR-1777b | IL-6 | 20 | 27 | bta-miR-2442 | | CCL2 | 15 | 22 |
| bta-miR-7864 | CD86 | 15 | 19 | | bta-miR-2462 | IL-6 | 13 | 14 | bta-miR-2881 | | CCL2 | 16 | 19 |
| bta-miR-7865 | CD86 | 16 | 19 | | bta-miR-664a | IL-6 | 17 | 21 | bta-miR-7865 | | CCL2 | 15 | 18 |
| bta-miR-8550 | CD86 | 13 | 18 | | bta-miR-2881 | IL-6 | 15 | 18 | bta-miR-10170-5p | | CCL2 | 14 | 17 |
| bta-miR-1842 | CD86 | 16 | 21 | | bta-miR-2888 | IL-6 | 15 | 17 | bta-miR-11972 | | CCL2 | 18 | 24 |
| bta-miR-10163-3p | CD86 | 18 | 21 | | bta-miR-2894 | IL-6 | 17 | 25 |  | |  |  |  |
| bta-miR-10164-3p | CD86 | 19 | 22 | | bta-miR-2902 | IL-6 | 17 | 19 |  | |  |  |  |
| bta-miR-10169-3p | CD86 | 13 | 15 | | bta-miR-2904 | IL-6 | 14 | 15 |  | |  |  |  |
| bta-miR-7857-3p | CD86 | 16 | 20 | | bta-miR-378c | IL-6 | 15 | 18 |  | |  |  |  |
| bta-miR-10175-3p | CD86 | 18 | 22 | | bta-miR-6523a | IL-6 | 18 | 40 |  | |  |  |  |
| bta-miR-10175-3p | CD86 | 18 | 20 | | bta-miR-7860 | IL-6 | 16 | 22 |  | |  |  |  |
| bta-miR-10178-5p | CD86 | 16 | 20 | | bta-miR-10167-3p | IL-6 | 15 | 19 |  | |  |  |  |
| bta-miR-668-3p | CD86 | 8 | 10 | | bta-miR-10171-3p | IL-6 | 18 | 24 |  | |  |  |  |
| bta-miR-10179-5p | CD86 | 17 | 27 | | bta-miR-10174-5p | IL-6 | 20 | 25 |  | |  |  |  |
| bta-miR-10183-5p | CD86 | 17 | 22 | | bta-miR-2285ah-5p | IL-6 | 19 | 23 |  | |  |  |  |
| bta-miR-10185-5p | CD86 | 13 | 19 | | bta-miR-11975 | IL-6 | 14 | 15 |  | |  |  |  |
| bta-miR-11974 | CD86 | 19 | 31 | | bta-miR-9851 | IL-6 | 17 | 22 |  | |  |  |  |
| bta-miR-11983 | CD86 | 17 | 27 | | bta-miR-11984 | IL-6 | 15 | 17 |  | |  |  |  |
| bta-miR-7180 | CD86 | 23 | 34 | | bta-miR-11989 | IL-6 | 18 | 34 |  | |  |  |  |
| bta-miR-2285bz | CD86 | 13 | 15 | | bta-miR-12001 | IL-6 | 17 | 25 |  | |  |  |  |
| bta-miR-11995 | CD86 | 18 | 23 | | bta-miR-12019 | IL-6 | 16 | 18 |  | |  |  |  |
| bta-miR-2285cb | CD86 | 12 | 15 | | bta-miR-12029 | IL-6 | 18 | 31 |  | |  |  |  |
| bta-miR-11997 | CD86 | 12 | 16 | | bta-miR-12039 | IL-6 | 21 | 44 |  | |  |  |  |
| bta-miR-12001 | CD86 | 20 | 25 | | bta-miR-12043 | IL-6 | 17 | 21 |  | |  |  |  |
| bta-miR-6775 | CD86 | 23 | 60 | | bta-miR-12045 | IL-6 | 14 | 17 |  | |  |  |  |
| bta-miR-12013 | CD86 | 17 | 26 | | bta-miR-12048 | IL-6 | 17 | 20 |  | |  |  |  |
| bta-miR-12014 | CD86 | 19 | 22 | | bta-miR-12050 | IL-6 | 13 | 14 |  | |  |  |  |
| bta-miR-12025 | CD86 | 17 | 23 | | bta-miR-12057 | IL-6 | 16 | 18 |  | |  |  |  |
| bta-miR-12028 | CD86 | 13 | 17 | | bta-miR-12060 | IL-6 | 14 | 16 |  | |  |  |  |
| bta-miR-12031 | CD86 | 14 | 18 | | bta-miR-151-5p | IFNγ | 18 | 23 |  | |  |  |  |
| bta-miR-12033 | CD86 | 13 | 16 | | bta-miR-205 | IFNγ | 16 | 19 |  | |  |  |  |
| bta-miR-12045 | CD86 | 12 | 20 | | bta-miR-20b | IFNγ | 18 | 23 |  | |  |  |  |
| bta-miR-507b | CD86 | 16 | 18 | | bta-miR-17-5p | IFNγ | 20 | 26 |  | |  |  |  |
| bta-miR-12050 | CD86 | 15 | 17 | | bta-miR-191 | IFNγ | 17 | 27 |  | |  |  |  |
| bta-miR-12053 | CD86 | 17 | 27 | | bta-miR-22-3p | IFNγ | 17 | 19 |  | |  |  |  |
| bta-miR-12054 | CD86 | 13 | 18 | | bta-let-7e | IFNγ | 12 | 13 |  | |  |  |  |
| bta-miR-12057 | CD86 | 15 | 18 | | bta-miR-24 | IFNγ | 10 | 13 |  | |  |  |  |
| bta-miR-12061 | CD86 | 15 | 18 | | bta-miR-28 | IFNγ | 12 | 14 |  | |  |  |  |
| bta-miR-30b-3p | IL-6 | 15 | 17 | | bta-miR-329b | IFNγ | 20 | 37 |  | |  |  |  |
| bta-miR-23b-3p | IL-6 | 16 | 18 | | bta-miR-330 | IFNγ | 18 | 22 |  | |  |  |  |
| bta-miR-134 | IL-6 | 19 | 28 | | bta-miR-339a | IFNγ | 20 | 38 |  | |  |  |  |
| bta-miR-2375 | IL-6 | 19 | 23 | | bta-miR-339a | IFNγ | 13 | 17 |  | |  |  |  |
| bta-miR-2420 | IL-6 | 14 | 16 | | bta-miR-453 | IFNγ | 20 | 29 |  | |  |  |  |
| bta-miR-2455 | IL-6 | 19 | 37 | | bta-miR-500 | IFNγ | 17 | 22 |  | |  |  |  |
| bta-miR-2893 | IL-6 | 18 | 20 | | bta-miR-504 | IFNγ | 16 | 18 |  | |  |  |  |
| bta-miR-149-3p | IL-6 | 17 | 23 | | bta-miR-615 | IFNγ | 18 | 23 |  | |  |  |  |
| bta-miR-7865 | IL-6 | 18 | 30 | | bta-miR-615 | IFNγ | 18 | 36 |  | |  |  |  |
| bta-miR-10180-3p | IL-6 | 12 | 16 | | bta-miR-671 | IFNγ | 18 | 23 |  | |  |  |  |
| bta-miR-12010 | IL-6 | 18 | 25 | | bta-miR-758 | IFNγ | 10 | 11 |  | |  |  |  |
| bta-miR-27a-5p | IFNγ | 14 | 15 | | bta-miR-760-3p | IFNγ | 17 | 24 |  | |  |  |  |
| bta-miR-484 | IFNγ | 10 | 13 | | bta-miR-763 | IFNγ | 16 | 21 |  | |  |  |  |
| bta-miR-22-3p | IFNγ | 12 | 14 | | bta-miR-769 | IFNγ | 14 | 20 |  | |  |  |  |
| bta-miR-93 | IFNγ | 15 | 20 | | bta-miR-874 | IFNγ | 17 | 21 |  | |  |  |  |
| bta-miR-24-3p | IFNγ | 12 | 17 | | bta-miR-1291 | IFNγ | 16 | 21 |  | |  |  |  |
| bta-miR-532 | IFNγ | 15 | 16 | | bta-miR-1835 | IFNγ | 13 | 16 |  | |  |  |  |
| bta-miR-346 | IFNγ | 13 | 14 | | bta-miR-1251 | IFNγ | 11 | 14 |  | |  |  |  |
| bta-miR-370 | IFNγ | 18 | 20 | | bta-miR-1296 | IFNγ | 10 | 11 |  | |  |  |  |
| bta-miR-1251 | IFNγ | 13 | 20 | | bta-miR-2295 | IFNγ | 16 | 30 |  | |  |  |  |
| bta-miR-2302 | IFNγ | 19 | 24 | | bta-miR-2313-5p | IFNγ | 14 | 19 |  | |  |  |  |
| bta-miR-2324 | IFNγ | 17 | 24 | | bta-miR-1343-5p | IFNγ | 13 | 16 |  | |  |  |  |
| bta-miR-2330-5p | IFNγ | 15 | 16 | | bta-miR-1343-3p | IFNγ | 11 | 13 |  | |  |  |  |
| bta-miR-2349 | IFNγ | 20 | 52 | | bta-miR-2328-5p | IFNγ | 14 | 18 |  | |  |  |  |
| bta-miR-2392 | IFNγ | 16 | 24 | | bta-miR-2328-3p | IFNγ | 15 | 17 |  | |  |  |  |
| bta-miR-2418 | IFNγ | 19 | 25 | | bta-miR-2331-5p | IFNγ | 21 | 30 |  | |  |  |  |
| bta-miR-2443 | IFNγ | 19 | 22 | | bta-miR-2368-5p | IFNγ | 16 | 18 |  | |  |  |  |
| bta-miR-2452 | IFNγ | 10 | 11 | | bta-miR-2373-3p | IFNγ | 20 | 30 |  | |  |  |  |
| bta-miR-2471-5p | IFNγ | 19 | 24 | | bta-miR-2413 | IFNγ | 17 | 19 |  | |  |  |  |
| bta-miR-2904 | IFNγ | 16 | 19 | | bta-miR-2415-3p | IFNγ | 15 | 22 |  | |  |  |  |
| bta-miR-3596 | IFNγ | 10 | 11 | | bta-miR-320b | IFNγ | 13 | 19 |  | |  |  |  |
| bta-miR-6521 | IFNγ | 16 | 23 | | bta-miR-2426 | IFNγ | 13 | 14 |  | |  |  |  |
| bta-miR-3956 | IFNγ | 17 | 22 | | bta-miR-2430 | IFNγ | 20 | 25 |  | |  |  |  |
| bta-miR-6535 | IFNγ | 13 | 16 | | bta-miR-2284r | IFNγ | 18 | 20 |  | |  |  |  |
| bta-miR-10165-5p | IFNγ | 14 | 16 | | bta-miR-2438 | IFNγ | 16 | 21 |  | |  |  |  |
| bta-miR-7180 | IFNγ | 14 | 21 | | bta-miR-2443 | IFNγ | 22 | 31 |  | |  |  |  |
| bta-miR-12000 | IFNγ | 11 | 12 | | bta-miR-2449 | IFNγ | 16 | 20 |  | |  |  |  |
| bta-miR-12014 | IFNγ | 17 | 22 | | bta-miR-2450a | IFNγ | 13 | 15 |  | |  |  |  |
| bta-miR-12054 | IFNγ | 13 | 18 | | bta-miR-1777a | IFNγ | 15 | 19 |  | |  |  |  |
| bta-miR-12056 | IFNγ | 19 | 23 | | bta-miR-2454-5p | IFNγ | 18 | 23 |  | |  |  |  |
| bta-miR-22-3p | CD4 | 14 | 18 | | bta-miR-2455 | IFNγ | 17 | 25 |  | |  |  |  |
| bta-miR-23a | CD4 | 14 | 17 | | bta-miR-2455 | IFNγ | 14 | 18 |  | |  |  |  |
| bta-miR-23b-5p | CD4 | 15 | 21 | | bta-miR-2460 | IFNγ | 15 | 20 |  | |  |  |  |
| bta-miR-133a | CD4 | 14 | 20 | | bta-miR-1777b | IFNγ | 14 | 18 |  | |  |  |  |
| bta-miR-133b | CD4 | 14 | 19 | | bta-miR-2464-3p | IFNγ | 15 | 21 |  | |  |  |  |
| bta-miR-197 | CD4 | 18 | 20 | | bta-miR-2467-3p | IFNγ | 18 | 20 |  | |  |  |  |
| bta-miR-328 | CD4 | 21 | 35 | | bta-miR-2881 | IFNγ | 13 | 16 |  | |  |  |  |
| bta-miR-339a | CD4 | 19 | 24 | | bta-miR-409b | IFNγ | 18 | 23 |  | |  |  |  |
| bta-miR-378 | CD4 | 17 | 20 | | bta-miR-26c | IFNγ | 15 | 17 |  | |  |  |  |
| bta-miR-382 | CD4 | 15 | 16 | | bta-miR-6526 | IFNγ | 16 | 20 |  | |  |  |  |
| bta-miR-433 | CD4 | 17 | 19 | | bta-miR-6529a | IFNγ | 12 | 16 |  | |  |  |  |
| bta-miR-449b | CD4 | 18 | 24 | | bta-miR-6532 | IFNγ | 16 | 21 |  | |  |  |  |
| bta-miR-483 | CD4 | 19 | 22 | | bta-miR-7691 | IFNγ | 10 | 12 |  | |  |  |  |
| bta-miR-500 | CD4 | 17 | 18 | | bta-miR-2285u | IFNγ | 14 | 18 |  | |  |  |  |
| bta-miR-502a | CD4 | 18 | 19 | | bta-miR-8550 | IFNγ | 16 | 20 |  | |  |  |  |
| bta-miR-504 | CD4 | 17 | 22 | | bta-miR-10175-3p | IFNγ | 11 | 14 |  | |  |  |  |
| bta-miR-658 | CD4 | 23 | 39 | | bta-miR-10182-3p | IFNγ | 16 | 22 |  | |  |  |  |
| bta-miR-671 | CD4 | 21 | 30 | | bta-miR-11972 | IFNγ | 14 | 16 |  | |  |  |  |
| bta-miR-873 | CD4 | 14 | 15 | | bta-miR-2285at | IFNγ | 13 | 16 |  | |  |  |  |
| bta-miR-877 | CD4 | 18 | 28 | | bta-miR-11981 | IFNγ | 18 | 25 |  | |  |  |  |
| bta-miR-935 | CD4 | 19 | 24 | | bta-miR-11995 | IFNγ | 17 | 25 |  | |  |  |  |
| bta-miR-376c | CD4 | 16 | 19 | | bta-miR-12001 | IFNγ | 16 | 23 |  | |  |  |  |
| bta-miR-1835 | CD4 | 17 | 23 | | bta-miR-12003 | IFNγ | 17 | 23 |  | |  |  |  |
| bta-miR-2290 | CD4 | 16 | 18 | | bta-miR-12004 | IFNγ | 14 | 22 |  | |  |  |  |
| bta-miR-2300a-5p | CD4 | 18 | 24 | | bta-miR-12011 | IFNγ | 13 | 16 |  | |  |  |  |
| bta-miR-2302 | CD4 | 19 | 22 | | bta-miR-12012 | IFNγ | 17 | 21 |  | |  |  |  |
| bta-miR-2304 | CD4 | 16 | 19 | | bta-miR-12015 | IFNγ | 14 | 17 |  | |  |  |  |
| bta-miR-2489 | CD4 | 13 | 15 | | bta-miR-12025 | IFNγ | 13 | 17 |  | |  |  |  |
| bta-miR-2313-5p | CD4 | 16 | 20 | | bta-miR-12029 | IFNγ | 18 | 23 |  | |  |  |  |
| bta-miR-2324 | CD4 | 18 | 24 | | bta-miR-12030 | IFNγ | 17 | 22 |  | |  |  |  |
| bta-miR-2328-3p | CD4 | 17 | 20 | | bta-miR-12034 | IFNγ | 16 | 22 |  | |  |  |  |
| bta-miR-2338 | CD4 | 15 | 19 | | bta-miR-12038 | IFNγ | 14 | 15 |  | |  |  |  |
| bta-miR-2341 | CD4 | 18 | 22 | | bta-miR-12039 | IFNγ | 10 | 12 |  | |  |  |  |
| bta-miR-2357 | CD4 | 18 | 23 | | bta-miR-12057 | IFNγ | 14 | 16 |  | |  |  |  |
| bta-miR-2360 | CD4 | 21 | 25 | | bta-miR-16b | CD4 | 11 | 13 |  | |  |  |  |
| bta-miR-2366 | CD4 | 18 | 23 | | bta-miR-21-3p | CD4 | 18 | 20 |  | |  |  |  |
| bta-miR-2368-3p | CD4 | 18 | 22 | | bta-miR-27a-5p | CD4 | 15 | 19 |  | |  |  |  |
| bta-miR-2369 | CD4 | 12 | 13 | | bta-miR-125a | CD4 | 14 | 15 |  | |  |  |  |
| bta-miR-2374 | CD4 | 16 | 18 | | bta-miR-199a-3p | CD4 | 15 | 16 |  | |  |  |  |
| bta-miR-2379 | CD4 | 13 | 16 | | bta-miR-24-3p | CD4 | 17 | 21 |  | |  |  |  |
| bta-miR-2388-3p | CD4 | 15 | 20 | | bta-let-7i | CD4 | 16 | 17 |  | |  |  |  |
| bta-miR-2389 | CD4 | 16 | 19 | | bta-miR-331-3p | CD4 | 14 | 17 |  | |  |  |  |
| bta-miR-2392 | CD4 | 16 | 20 | | bta-miR-34a | CD4 | 16 | 18 |  | |  |  |  |
| bta-miR-2396 | CD4 | 16 | 18 | | bta-miR-497 | CD4 | 16 | 18 |  | |  |  |  |
| bta-miR-2401 | CD4 | 17 | 21 | | bta-miR-133a | CD4 | 17 | 20 |  | |  |  |  |
| bta-miR-2404 | CD4 | 15 | 17 | | bta-miR-133b | CD4 | 16 | 19 |  | |  |  |  |
| bta-miR-2406 | CD4 | 14 | 17 | | bta-miR-16a | CD4 | 11 | 12 |  | |  |  |  |
| bta-miR-2408 | CD4 | 16 | 18 | | bta-miR-183 | CD4 | 11 | 12 |  | |  |  |  |
| bta-miR-2409 | CD4 | 15 | 19 | | bta-miR-188 | CD4 | 19 | 34 |  | |  |  |  |
| bta-miR-2412 | CD4 | 17 | 21 | | bta-miR-223 | CD4 | 15 | 16 |  | |  |  |  |
| bta-miR-2422 | CD4 | 17 | 21 | | bta-miR-29d-5p | CD4 | 16 | 20 |  | |  |  |  |
| bta-miR-2422 | CD4 | 16 | 18 | | bta-miR-29d-5p | CD4 | 15 | 17 |  | |  |  |  |
| bta-miR-1843 | CD4 | 21 | 31 | | bta-miR-326 | CD4 | 15 | 17 |  | |  |  |  |
| bta-miR-320b | CD4 | 18 | 43 | | bta-miR-33b | CD4 | 18 | 41 |  | |  |  |  |
| bta-miR-320b | CD4 | 19 | 58 | | bta-miR-346 | CD4 | 15 | 18 |  | |  |  |  |
| bta-miR-2423 | CD4 | 16 | 18 | | bta-miR-362-5p | CD4 | 15 | 20 |  | |  |  |  |
| bta-miR-2428 | CD4 | 17 | 23 | | bta-miR-375 | CD4 | 20 | 34 |  | |  |  |  |
| bta-miR-2430 | CD4 | 20 | 24 | | bta-miR-409a | CD4 | 18 | 36 |  | |  |  |  |
| bta-miR-2432 | CD4 | 16 | 20 | | bta-miR-412 | CD4 | 16 | 18 |  | |  |  |  |
| bta-miR-2432 | CD4 | 19 | 23 | | bta-miR-490 | CD4 | 17 | 20 |  | |  |  |  |
| bta-miR-2436-3p | CD4 | 15 | 16 | | bta-miR-493 | CD4 | 14 | 18 |  | |  |  |  |
| bta-miR-2439-3p | CD4 | 16 | 20 | | bta-miR-502b | CD4 | 16 | 20 |  | |  |  |  |
| bta-miR-2450a | CD4 | 16 | 21 | | bta-miR-631 | CD4 | 15 | 18 |  | |  |  |  |
| bta-miR-2451 | CD4 | 16 | 18 | | bta-miR-744 | CD4 | 17 | 19 |  | |  |  |  |
| bta-miR-1777a | CD4 | 18 | 24 | | bta-miR-744 | CD4 | 19 | 28 |  | |  |  |  |
| bta-miR-2454-5p | CD4 | 21 | 35 | | bta-miR-761 | CD4 | 15 | 20 |  | |  |  |  |
| bta-miR-339b | CD4 | 16 | 21 | | bta-miR-767 | CD4 | 15 | 18 |  | |  |  |  |
| bta-miR-2464-5p | CD4 | 17 | 22 | | bta-miR-873 | CD4 | 19 | 40 |  | |  |  |  |
| bta-miR-2465 | CD4 | 15 | 16 | | bta-miR-874 | CD4 | 16 | 23 |  | |  |  |  |
| bta-miR-2467-3p | CD4 | 18 | 31 | | bta-miR-92b | CD4 | 18 | 21 |  | |  |  |  |
| bta-miR-2470 | CD4 | 14 | 15 | | bta-miR-940 | CD4 | 16 | 21 |  | |  |  |  |
| bta-miR-2474 | CD4 | 20 | 34 | | bta-miR-1835 | CD4 | 19 | 38 |  | |  |  |  |
| bta-miR-2478 | CD4 | 15 | 20 | | bta-miR-1296 | CD4 | 15 | 18 |  | |  |  |  |
| bta-miR-1388-3p | CD4 | 17 | 22 | | bta-miR-1307 | CD4 | 18 | 24 |  | |  |  |  |
| bta-miR-2887 | CD4 | 17 | 21 | | bta-miR-1307 | CD4 | 16 | 23 |  | |  |  |  |
| bta-miR-2889 | CD4 | 17 | 21 | | bta-miR-2294 | CD4 | 18 | 39 |  | |  |  |  |
| bta-miR-2893 | CD4 | 17 | 23 | | bta-miR-2301 | CD4 | 14 | 15 |  | |  |  |  |
| bta-miR-1246 | CD4 | 14 | 15 | | bta-miR-2331-3p | CD4 | 14 | 22 |  | |  |  |  |
| bta-miR-6121-5p | CD4 | 15 | 19 | | bta-miR-2334 | CD4 | 20 | 40 |  | |  |  |  |
| bta-miR-6517 | CD4 | 18 | 23 | | bta-miR-2334 | CD4 | 19 | 27 |  | |  |  |  |
| bta-miR-3154 | CD4 | 18 | 27 | | bta-miR-2342 | CD4 | 15 | 17 |  | |  |  |  |
| bta-miR-378b | CD4 | 17 | 23 | | bta-miR-2344 | CD4 | 17 | 20 |  | |  |  |  |
| bta-miR-3957 | CD4 | 15 | 20 | | bta-miR-2360 | CD4 | 18 | 42 |  | |  |  |  |
| bta-miR-6526 | CD4 | 15 | 18 | | bta-miR-2373-5p | CD4 | 19 | 23 |  | |  |  |  |
| bta-miR-6534 | CD4 | 14 | 16 | | bta-miR-2376 | CD4 | 19 | 23 |  | |  |  |  |
| bta-miR-6535 | CD4 | 15 | 18 | | bta-miR-2378 | CD4 | 17 | 19 |  | |  |  |  |
| bta-miR-6535 | CD4 | 18 | 21 | | bta-miR-2379 | CD4 | 14 | 15 |  | |  |  |  |
| bta-miR-7857-5p | CD4 | 13 | 15 | | bta-miR-2394 | CD4 | 19 | 27 |  | |  |  |  |
| bta-miR-7857-5p | CD4 | 18 | 23 | | bta-miR-2403 | CD4 | 14 | 18 |  | |  |  |  |
| bta-miR-7864 | CD4 | 17 | 24 | | bta-miR-1584-5p | CD4 | 18 | 19 |  | |  |  |  |
| bta-miR-8549 | CD4 | 12 | 14 | | bta-miR-2415-5p | CD4 | 20 | 52 |  | |  |  |  |
| bta-miR-378d | CD4 | 17 | 21 | | bta-miR-2417 | CD4 | 11 | 14 |  | |  |  |  |
| bta-miR-10165-5p | CD4 | 18 | 26 | | bta-miR-2418 | CD4 | 17 | 20 |  | |  |  |  |
| bta-miR-10167-3p | CD4 | 20 | 38 | | bta-miR-2425-5p | CD4 | 19 | 23 |  | |  |  |  |
| bta-miR-10169-3p | CD4 | 10 | 11 | | bta-miR-2426 | CD4 | 14 | 17 |  | |  |  |  |
| bta-miR-10172-3p | CD4 | 17 | 21 | | bta-miR-2432 | CD4 | 18 | 22 |  | |  |  |  |
| bta-miR-668-3p | CD4 | 16 | 21 | | bta-miR-2433 | CD4 | 14 | 17 |  | |  |  |  |
| bta-miR-10180-3p | CD4 | 15 | 19 | | bta-miR-2443 | CD4 | 19 | 24 |  | |  |  |  |
| bta-miR-10181-5p | CD4 | 18 | 20 | | bta-miR-2447 | CD4 | 19 | 26 |  | |  |  |  |
| bta-miR-10182-3p | CD4 | 19 | 23 | | bta-miR-2449 | CD4 | 16 | 23 |  | |  |  |  |
| bta-miR-10185-5p | CD4 | 18 | 23 | | bta-miR-2450c | CD4 | 17 | 28 |  | |  |  |  |
| bta-miR-2285ah-5p | CD4 | 19 | 28 | | bta-miR-2460 | CD4 | 17 | 21 |  | |  |  |  |
| bta-miR-2285ak-5p | CD4 | 16 | 17 | | bta-miR-2461-3p | CD4 | 20 | 22 |  | |  |  |  |
| bta-miR-11971 | CD4 | 11 | 12 | | bta-miR-2467-3p | CD4 | 18 | 25 |  | |  |  |  |
| bta-miR-2285an | CD4 | 14 | 16 | | bta-miR-2472 | CD4 | 17 | 20 |  | |  |  |  |
| bta-miR-2285aq | CD4 | 14 | 16 | | bta-miR-2883 | CD4 | 13 | 15 |  | |  |  |  |
| bta-miR-9851 | CD4 | 18 | 26 | | bta-miR-2897 | CD4 | 16 | 21 |  | |  |  |  |
| bta-miR-11987 | CD4 | 14 | 21 | | bta-miR-1260b | CD4 | 11 | 15 |  | |  |  |  |
| bta-miR-2285bi | CD4 | 14 | 16 | | bta-miR-6519 | CD4 | 14 | 17 |  | |  |  |  |
| bta-miR-11991 | CD4 | 13 | 15 | | bta-miR-6521 | CD4 | 18 | 22 |  | |  |  |  |
| bta-miR-11995 | CD4 | 17 | 20 | | bta-miR-3956 | CD4 | 17 | 20 |  | |  |  |  |
| bta-miR-2285ce | CD4 | 17 | 21 | | bta-miR-6523a | CD4 | 20 | 26 |  | |  |  |  |
| bta-miR-12004 | CD4 | 19 | 26 | | bta-miR-503-5p | CD4 | 14 | 17 |  | |  |  |  |
| bta-miR-12011 | CD4 | 13 | 16 | | bta-miR-503-3p | CD4 | 19 | 24 |  | |  |  |  |
| bta-miR-12015 | CD4 | 19 | 40 | | bta-miR-7691 | CD4 | 12 | 17 |  | |  |  |  |
| bta-miR-12029 | CD4 | 15 | 21 | | bta-miR-7857-5p | CD4 | 11 | 12 |  | |  |  |  |
| bta-miR-12030 | CD4 | 16 | 18 | | bta-miR-7859 | CD4 | 12 | 15 |  | |  |  |  |
| bta-miR-12036 | CD4 | 20 | 42 | | bta-miR-8550 | CD4 | 16 | 21 |  | |  |  |  |
| bta-miR-12039 | CD4 | 21 | 26 | | bta-miR-10167-3p | CD4 | 17 | 23 |  | |  |  |  |
| bta-miR-12043 | CD4 | 17 | 22 | | bta-miR-10168-5p | CD4 | 18 | 25 |  | |  |  |  |
| bta-miR-12043 | CD4 | 18 | 23 | | bta-miR-10174-5p | CD4 | 18 | 35 |  | |  |  |  |
| bta-miR-12045 | CD4 | 16 | 21 | | bta-miR-668-3p | CD4 | 18 | 29 |  | |  |  |  |
| bta-miR-12046 | CD4 | 14 | 17 | | bta-miR-10180-3p | CD4 | 15 | 18 |  | |  |  |  |
| bta-miR-12054 | CD4 | 14 | 18 | | bta-miR-10181-5p | CD4 | 17 | 22 |  | |  |  |  |
| bta-miR-12060 | CD4 | 13 | 21 | | bta-miR-11972 | CD4 | 18 | 24 |  | |  |  |  |
| bta-miR-22-3p | CD4 | 14 | 18 | | bta-miR-11973 | CD4 | 19 | 22 |  | |  |  |  |
| bta-miR-23a | CD4 | 14 | 17 | | bta-miR-1911 | CD4 | 21 | 43 |  | |  |  |  |
| bta-miR-23b-5p | CD4 | 15 | 21 | | bta-miR-12004 | CD4 | 19 | 23 |  | |  |  |  |
| bta-miR-133a | CD4 | 14 | 20 | | bta-miR-148d | CD4 | 14 | 20 |  | |  |  |  |
| bta-miR-133b | CD4 | 14 | 19 | | bta-miR-12027 | CD4 | 14 | 16 |  | |  |  |  |
| bta-miR-183 | CD4 | 11 | 12 | | bta-miR-12033 | CD4 | 14 | 17 |  | |  |  |  |
| bta-miR-197 | CD4 | 18 | 20 | | bta-miR-12041 | CD4 | 16 | 18 |  | |  |  |  |
| bta-miR-29d-5p | CD4 | 16 | 20 | | bta-miR-12045 | CD4 | 16 | 28 |  | |  |  |  |
| bta-miR-328 | CD4 | 21 | 35 | | bta-miR-2285dj | CD4 | 20 | 25 |  | |  |  |  |
| bta-miR-329b | CD4 | 16 | 19 | | bta-miR-12049 | CD4 | 19 | 25 |  | |  |  |  |
| bta-miR-339a | CD4 | 19 | 24 | | bta-miR-12051 | CD4 | 17 | 23 |  | |  |  |  |
| bta-miR-378 | CD4 | 17 | 20 | | bta-miR-16b | CD4 | 11 | 13 |  | |  |  |  |
| bta-miR-382 | CD4 | 15 | 16 | | bta-miR-21-3p | CD4 | 18 | 20 |  | |  |  |  |
| bta-miR-433 | CD4 | 17 | 19 | | bta-miR-27a-5p | CD4 | 15 | 19 |  | |  |  |  |
| bta-miR-449b | CD4 | 18 | 24 | | bta-miR-125a | CD4 | 14 | 15 |  | |  |  |  |
| bta-miR-483 | CD4 | 19 | 22 | | bta-miR-199a-3p | CD4 | 15 | 16 |  | |  |  |  |
| bta-miR-500 | CD4 | 17 | 18 | | bta-miR-24-3p | CD4 | 17 | 21 |  | |  |  |  |
| bta-miR-502a | CD4 | 18 | 19 | | bta-let-7i | CD4 | 16 | 17 |  | |  |  |  |
| bta-miR-504 | CD4 | 17 | 22 | | bta-miR-331-3p | CD4 | 14 | 17 |  | |  |  |  |
| bta-miR-631 | CD4 | 15 | 18 | | bta-miR-34a | CD4 | 16 | 18 |  | |  |  |  |
| bta-miR-671 | CD4 | 21 | 30 | | bta-miR-497 | CD4 | 16 | 18 |  | |  |  |  |
| bta-miR-873 | CD4 | 14 | 15 | | bta-miR-133a | CD4 | 17 | 20 |  | |  |  |  |
| bta-miR-877 | CD4 | 18 | 28 | | bta-miR-133b | CD4 | 16 | 19 |  | |  |  |  |
| bta-miR-935 | CD4 | 19 | 24 | | bta-miR-16a | CD4 | 11 | 12 |  | |  |  |  |
| bta-miR-376c | CD4 | 16 | 19 | | bta-miR-188 | CD4 | 19 | 34 |  | |  |  |  |
| bta-miR-1835 | CD4 | 17 | 23 | | bta-miR-223 | CD4 | 15 | 16 |  | |  |  |  |
| bta-miR-2290 | CD4 | 16 | 18 | | bta-miR-29d-5p | CD4 | 15 | 17 |  | |  |  |  |
| bta-miR-2294 | CD4 | 18 | 39 | | bta-miR-326 | CD4 | 15 | 17 |  | |  |  |  |
| bta-miR-2294 | CD4 | 15 | 18 | | bta-miR-33b | CD4 | 18 | 41 |  | |  |  |  |
| bta-miR-2300a-5p | CD4 | 18 | 24 | | bta-miR-362-5p | CD4 | 15 | 20 |  | |  |  |  |
| bta-miR-2302 | CD4 | 19 | 22 | | bta-miR-375 | CD4 | 20 | 35 |  | |  |  |  |
| bta-miR-2304 | CD4 | 16 | 19 | | bta-miR-409a | CD4 | 18 | 36 |  | |  |  |  |
| bta-miR-2308 | CD4 | 14 | 17 | | bta-miR-412 | CD4 | 16 | 18 |  | |  |  |  |
| bta-miR-2489 | CD4 | 13 | 15 | | bta-miR-432 | CD4 | 18 | 21 |  | |  |  |  |
| bta-miR-2313-5p | CD4 | 16 | 20 | | bta-miR-490 | CD4 | 17 | 20 |  | |  |  |  |
| bta-miR-2323 | CD4 | 18 | 25 | | bta-miR-502b | CD4 | 16 | 20 |  | |  |  |  |
| bta-miR-2324 | CD4 | 18 | 24 | | bta-miR-615 | CD4 | 17 | 18 |  | |  |  |  |
| bta-miR-2328-3p | CD4 | 17 | 20 | | bta-miR-744 | CD4 | 17 | 19 |  | |  |  |  |
| bta-miR-2338 | CD4 | 15 | 19 | | bta-miR-744 | CD4 | 19 | 28 |  | |  |  |  |
| bta-miR-2341 | CD4 | 18 | 22 | | bta-miR-763 | CD4 | 19 | 26 |  | |  |  |  |
| bta-miR-2357 | CD4 | 18 | 23 | | bta-miR-92b | CD4 | 18 | 21 |  | |  |  |  |
| bta-miR-2360 | CD4 | 21 | 25 | | bta-miR-940 | CD4 | 16 | 21 |  | |  |  |  |
| bta-miR-2366 | CD4 | 18 | 23 | | bta-miR-1296 | CD4 | 15 | 18 |  | |  |  |  |
| bta-miR-2368-3p | CD4 | 18 | 22 | | bta-miR-2284i | CD4 | 15 | 18 |  | |  |  |  |
| bta-miR-2369 | CD4 | 12 | 13 | | bta-miR-2299-3p | CD4 | 14 | 20 |  | |  |  |  |
| bta-miR-2373-5p | CD4 | 19 | 23 | | bta-miR-2301 | CD4 | 14 | 15 |  | |  |  |  |
| bta-miR-2373-5p | CD4 | 17 | 22 | | bta-miR-2331-3p | CD4 | 14 | 22 |  | |  |  |  |
| bta-miR-2374 | CD4 | 16 | 18 | | bta-miR-2338 | CD4 | 19 | 24 |  | |  |  |  |
| bta-miR-2379 | CD4 | 13 | 16 | | bta-miR-2342 | CD4 | 15 | 17 |  | |  |  |  |
| bta-miR-2388-3p | CD4 | 15 | 20 | | bta-miR-2344 | CD4 | 17 | 20 |  | |  |  |  |
| bta-miR-2389 | CD4 | 16 | 19 | | bta-miR-2376 | CD4 | 19 | 23 |  | |  |  |  |
| bta-miR-2392 | CD4 | 16 | 20 | | bta-miR-2378 | CD4 | 17 | 19 |  | |  |  |  |
| bta-miR-2394 | CD4 | 15 | 19 | | bta-miR-2379 | CD4 | 14 | 15 |  | |  |  |  |
| bta-miR-2401 | CD4 | 17 | 21 | | bta-miR-2403 | CD4 | 14 | 18 |  | |  |  |  |
| bta-miR-2404 | CD4 | 15 | 17 | | bta-miR-2415-5p | CD4 | 20 | 52 |  | |  |  |  |
| bta-miR-2406 | CD4 | 14 | 17 | | bta-miR-2418 | CD4 | 17 | 20 |  | |  |  |  |
| bta-miR-2408 | CD4 | 16 | 18 | | bta-miR-2425-5p | CD4 | 19 | 23 |  | |  |  |  |
| bta-miR-2409 | CD4 | 15 | 19 | | bta-miR-2426 | CD4 | 14 | 17 |  | |  |  |  |
| bta-miR-1584-5p | CD4 | 18 | 19 | | bta-miR-2432 | CD4 | 18 | 22 |  | |  |  |  |
| bta-miR-2412 | CD4 | 17 | 21 | | bta-miR-2433 | CD4 | 14 | 17 |  | |  |  |  |
| bta-miR-2417 | CD4 | 11 | 14 | | bta-miR-2442 | CD4 | 18 | 27 |  | |  |  |  |
| bta-miR-2422 | CD4 | 17 | 21 | | bta-miR-2443 | CD4 | 19 | 24 |  | |  |  |  |
| bta-miR-2422 | CD4 | 16 | 18 | | bta-miR-2447 | CD4 | 19 | 26 |  | |  |  |  |
| bta-miR-1843 | CD4 | 21 | 31 | | bta-miR-2461-3p | CD4 | 20 | 22 |  | |  |  |  |
| bta-miR-320b | CD4 | 18 | 43 | | bta-miR-2467-5p | CD4 | 15 | 17 |  | |  |  |  |
| bta-miR-320b | CD4 | 19 | 58 | | bta-miR-2467-3p | CD4 | 17 | 23 |  | |  |  |  |
| bta-miR-2423 | CD4 | 16 | 18 | | bta-miR-2472 | CD4 | 17 | 20 |  | |  |  |  |
| bta-miR-2428 | CD4 | 17 | 23 | | bta-miR-2883 | CD4 | 13 | 15 |  | |  |  |  |
| bta-miR-2430 | CD4 | 20 | 24 | | bta-miR-2897 | CD4 | 16 | 21 |  | |  |  |  |
| bta-miR-2432 | CD4 | 16 | 20 | | bta-miR-1260b | CD4 | 11 | 15 |  | |  |  |  |
| bta-miR-2432 | CD4 | 19 | 23 | | bta-miR-6519 | CD4 | 14 | 17 |  | |  |  |  |
| bta-miR-2433 | CD4 | 15 | 21 | | bta-miR-6521 | CD4 | 18 | 22 |  | |  |  |  |
| bta-miR-2436-5p | CD4 | 15 | 19 | | bta-miR-503-5p | CD4 | 14 | 17 |  | |  |  |  |
| bta-miR-2436-3p | CD4 | 15 | 16 | | bta-miR-503-3p | CD4 | 19 | 24 |  | |  |  |  |
| bta-miR-2439-3p | CD4 | 16 | 20 | | bta-miR-7691 | CD4 | 12 | 17 |  | |  |  |  |
| bta-miR-2450a | CD4 | 16 | 21 | | bta-miR-7857-5p | CD4 | 11 | 12 |  | |  |  |  |
| bta-miR-2450a | CD4 | 15 | 16 | | bta-miR-8550 | CD4 | 16 | 21 |  | |  |  |  |
| bta-miR-2451 | CD4 | 16 | 18 | | bta-miR-10167-3p | CD4 | 17 | 23 |  | |  |  |  |
| bta-miR-1777a | CD4 | 18 | 24 | | bta-miR-10168-5p | CD4 | 18 | 25 |  | |  |  |  |
| bta-miR-2454-5p | CD4 | 21 | 35 | | bta-miR-10174-5p | CD4 | 18 | 35 |  | |  |  |  |
| bta-miR-2454-5p | CD4 | 18 | 23 | | bta-miR-10174-3p | CD4 | 16 | 21 |  | |  |  |  |
| bta-miR-339b | CD4 | 16 | 21 | | bta-miR-668-3p | CD4 | 18 | 29 |  | |  |  |  |
| bta-miR-2464-5p | CD4 | 17 | 22 | | bta-miR-10180-3p | CD4 | 15 | 18 |  | |  |  |  |
| bta-miR-2465 | CD4 | 15 | 16 | | bta-miR-10181-5p | CD4 | 17 | 22 |  | |  |  |  |
| bta-miR-2467-3p | CD4 | 18 | 31 | | bta-miR-11973 | CD4 | 19 | 22 |  | |  |  |  |
| bta-miR-2470 | CD4 | 14 | 15 | | bta-miR-1911 | CD4 | 21 | 43 |  | |  |  |  |
| bta-miR-2474 | CD4 | 20 | 34 | | bta-miR-12001 | CD4 | 17 | 18 |  | |  |  |  |
| bta-miR-2478 | CD4 | 15 | 20 | | bta-miR-12004 | CD4 | 19 | 23 |  | |  |  |  |
| bta-miR-1388-3p | CD4 | 17 | 22 | | bta-miR-148d | CD4 | 14 | 20 |  | |  |  |  |
| bta-miR-2887 | CD4 | 17 | 21 | | bta-miR-12027 | CD4 | 14 | 16 |  | |  |  |  |
| bta-miR-2889 | CD4 | 17 | 21 | | bta-miR-12033 | CD4 | 14 | 17 |  | |  |  |  |
| bta-miR-2893 | CD4 | 17 | 23 | | bta-miR-12041 | CD4 | 16 | 18 |  | |  |  |  |
| bta-miR-1246 | CD4 | 14 | 15 | | bta-miR-12045 | CD4 | 16 | 28 |  | |  |  |  |
| bta-miR-6121-5p | CD4 | 15 | 19 | | bta-miR-2285dj | CD4 | 20 | 25 |  | |  |  |  |
| bta-miR-6517 | CD4 | 18 | 23 | | bta-miR-12049 | CD4 | 19 | 25 |  | |  |  |  |
| bta-miR-3154 | CD4 | 18 | 27 | | bta-miR-12051 | CD4 | 17 | 23 |  | |  |  |  |
| bta-miR-378b | CD4 | 17 | 23 | | bta-miR-1949 | CD4 | 11 | 14 |  | |  |  |  |
| bta-miR-3957 | CD4 | 15 | 20 | | bta-miR-103 | CXCL8 | 17 | 20 |  | |  |  |  |
| bta-miR-6534 | CD4 | 14 | 16 | | bta-miR-27a-5p | CXCL8 | 18 | 27 |  | |  |  |  |
| bta-miR-6535 | CD4 | 15 | 18 | | bta-miR-128 | CXCL8 | 15 | 21 |  | |  |  |  |
| bta-miR-6535 | CD4 | 18 | 21 | | bta-miR-107 | CXCL8 | 17 | 19 |  | |  |  |  |
| bta-miR-7857-5p | CD4 | 13 | 15 | | bta-miR-127 | CXCL8 | 18 | 25 |  | |  |  |  |
| bta-miR-7857-5p | CD4 | 18 | 23 | | bta-miR-214 | CXCL8 | 15 | 22 |  | |  |  |  |
| bta-miR-7859 | CD4 | 12 | 15 | | bta-miR-423-5p | CXCL8 | 15 | 20 |  | |  |  |  |
| bta-miR-7864 | CD4 | 17 | 24 | | bta-miR-197 | CXCL8 | 16 | 20 |  | |  |  |  |
| bta-miR-8549 | CD4 | 12 | 14 | | bta-miR-658 | CXCL8 | 17 | 23 |  | |  |  |  |
| bta-miR-378d | CD4 | 17 | 21 | | bta-miR-1251 | CXCL8 | 13 | 17 |  | |  |  |  |
| bta-miR-10165-5p | CD4 | 18 | 26 | | bta-miR-2294 | CXCL8 | 14 | 15 |  | |  |  |  |
| bta-miR-10167-3p | CD4 | 20 | 38 | | bta-miR-2302 | CXCL8 | 14 | 21 |  | |  |  |  |
| bta-miR-10169-3p | CD4 | 10 | 11 | | bta-miR-2305 | CXCL8 | 17 | 20 |  | |  |  |  |
| bta-miR-10172-3p | CD4 | 17 | 21 | | bta-miR-1343-5p | CXCL8 | 16 | 22 |  | |  |  |  |
| bta-miR-668-3p | CD4 | 16 | 21 | | bta-miR-2320-5p | CXCL8 | 19 | 36 |  | |  |  |  |
| bta-miR-10180-3p | CD4 | 15 | 19 | | bta-miR-2382-5p | CXCL8 | 17 | 21 |  | |  |  |  |
| bta-miR-10181-5p | CD4 | 18 | 20 | | bta-miR-2392 | CXCL8 | 19 | 23 |  | |  |  |  |
| bta-miR-10182-3p | CD4 | 19 | 23 | | bta-miR-2395 | CXCL8 | 16 | 22 |  | |  |  |  |
| bta-miR-10185-5p | CD4 | 18 | 23 | | bta-miR-2408 | CXCL8 | 14 | 15 |  | |  |  |  |
| bta-miR-2285ah-5p | CD4 | 17 | 23 | | bta-miR-1584-3p | CXCL8 | 20 | 28 |  | |  |  |  |
| bta-miR-2285ak-5p | CD4 | 16 | 17 | | bta-miR-2411-5p | CXCL8 | 13 | 15 |  | |  |  |  |
| bta-miR-11971 | CD4 | 11 | 12 | | bta-miR-2433 | CXCL8 | 13 | 21 |  | |  |  |  |
| bta-miR-2285an | CD4 | 14 | 16 | | bta-miR-2436-5p | CXCL8 | 21 | 35 |  | |  |  |  |
| bta-miR-2285aq | CD4 | 14 | 16 | | bta-miR-2438 | CXCL8 | 15 | 21 |  | |  |  |  |
| bta-miR-9851 | CD4 | 18 | 26 | | bta-miR-2442 | CXCL8 | 21 | 30 |  | |  |  |  |
| bta-miR-11987 | CD4 | 14 | 21 | | bta-miR-1777a | CXCL8 | 17 | 19 |  | |  |  |  |
| bta-miR-2285bi | CD4 | 14 | 16 | | bta-miR-2454-5p | CXCL8 | 19 | 24 |  | |  |  |  |
| bta-miR-11991 | CD4 | 13 | 15 | | bta-miR-1777b | CXCL8 | 18 | 20 |  | |  |  |  |
| bta-miR-11995 | CD4 | 17 | 20 | | bta-miR-2467-3p | CXCL8 | 20 | 27 |  | |  |  |  |
| bta-miR-2285ce | CD4 | 17 | 21 | | bta-miR-2881 | CXCL8 | 15 | 19 |  | |  |  |  |
| bta-miR-12004 | CD4 | 19 | 26 | | bta-miR-2899 | CXCL8 | 14 | 23 |  | |  |  |  |
| bta-miR-12011 | CD4 | 13 | 16 | | bta-miR-149-3p | CXCL8 | 16 | 20 |  | |  |  |  |
| bta-miR-12015 | CD4 | 19 | 40 | | bta-miR-574 | CXCL8 | 16 | 22 |  | |  |  |  |
| bta-miR-12029 | CD4 | 15 | 21 | | bta-miR-6123 | CXCL8 | 11 | 13 |  | |  |  |  |
| bta-miR-12030 | CD4 | 16 | 18 | | bta-miR-6521 | CXCL8 | 14 | 16 |  | |  |  |  |
| bta-miR-12030 | CD4 | 15 | 19 | | bta-miR-7860 | CXCL8 | 17 | 22 |  | |  |  |  |
| bta-miR-12036 | CD4 | 20 | 42 | | bta-miR-7865 | CXCL8 | 14 | 16 |  | |  |  |  |
| bta-miR-12039 | CD4 | 21 | 26 | | bta-miR-10175-3p | CXCL8 | 13 | 17 |  | |  |  |  |
| bta-miR-12043 | CD4 | 17 | 22 | | bta-miR-194b-3p | CXCL8 | 12 | 14 |  | |  |  |  |
| bta-miR-12043 | CD4 | 18 | 23 | | bta-miR-2285ah-5p | CXCL8 | 16 | 20 |  | |  |  |  |
| bta-miR-12045 | CD4 | 16 | 21 | | bta-miR-11974 | CXCL8 | 12 | 15 |  | |  |  |  |
| bta-miR-12046 | CD4 | 14 | 17 | | bta-miR-6715 | CXCL8 | 15 | 17 |  | |  |  |  |
| bta-miR-12054 | CD4 | 14 | 18 | | bta-miR-11995 | CXCL8 | 16 | 21 |  | |  |  |  |
| bta-miR-12060 | CD4 | 13 | 21 | | bta-miR-12007 | CXCL8 | 19 | 23 |  | |  |  |  |
| bta-miR-125b | CXCL8 | 13 | 20 | | bta-miR-12025 | CXCL8 | 16 | 18 |  | |  |  |  |
| bta-miR-31 | CXCL8 | 17 | 25 | | bta-miR-12025 | CXCL8 | 12 | 15 |  | |  |  |  |
| bta-miR-15b | CXCL8 | 14 | 17 | | bta-miR-126-3p | ICAM1 | 12 | 16 |  | |  |  |  |
| bta-miR-124a | CXCL8 | 17 | 24 | | bta-miR-199a-5p | ICAM1 | 18 | 31 |  | |  |  |  |
| bta-miR-199b | CXCL8 | 14 | 16 | | bta-miR-199a-5p | ICAM1 | 14 | 16 |  | |  |  |  |
| bta-miR-210 | CXCL8 | 17 | 18 | | bta-miR-193a-3p | ICAM1 | 13 | 19 |  | |  |  |  |
| bta-miR-455-3p | CXCL8 | 14 | 17 | | bta-miR-22-5p | ICAM1 | 16 | 18 |  | |  |  |  |
| bta-miR-24-3p | CXCL8 | 17 | 21 | | bta-miR-24-3p | ICAM1 | 17 | 23 |  | |  |  |  |
| bta-let-7e | CXCL8 | 15 | 20 | | bta-miR-129 | ICAM1 | 13 | 16 |  | |  |  |  |
| bta-miR-365-5p | CXCL8 | 18 | 22 | | bta-miR-129-5p | ICAM1 | 13 | 16 |  | |  |  |  |
| bta-miR-365-5p | CXCL8 | 14 | 17 | | bta-miR-133a | ICAM1 | 16 | 21 |  | |  |  |  |
| bta-miR-124b | CXCL8 | 17 | 24 | | bta-miR-153 | ICAM1 | 15 | 20 |  | |  |  |  |
| bta-miR-183 | CXCL8 | 12 | 16 | | bta-miR-154a | ICAM1 | 15 | 17 |  | |  |  |  |
| bta-miR-188 | CXCL8 | 18 | 23 | | bta-miR-183 | ICAM1 | 10 | 11 |  | |  |  |  |
| bta-miR-188 | CXCL8 | 19 | 21 | | bta-miR-24 | ICAM1 | 17 | 21 |  | |  |  |  |
| bta-miR-302d | CXCL8 | 17 | 21 | | bta-miR-196b | ICAM1 | 20 | 28 |  | |  |  |  |
| bta-miR-302b | CXCL8 | 17 | 21 | | bta-miR-196b | ICAM1 | 16 | 19 |  | |  |  |  |
| bta-miR-302c | CXCL8 | 18 | 22 | | bta-miR-28 | ICAM1 | 13 | 16 |  | |  |  |  |
| bta-miR-504 | CXCL8 | 10 | 12 | | bta-miR-326 | ICAM1 | 19 | 33 |  | |  |  |  |
| bta-miR-615 | CXCL8 | 13 | 17 | | bta-miR-326 | ICAM1 | 19 | 46 |  | |  |  |  |
| bta-miR-744 | CXCL8 | 16 | 30 | | bta-miR-328 | ICAM1 | 22 | 57 |  | |  |  |  |
| bta-miR-760-5p | CXCL8 | 15 | 17 | | bta-miR-330 | ICAM1 | 17 | 23 |  | |  |  |  |
| bta-miR-763 | CXCL8 | 18 | 20 | | bta-miR-338 | ICAM1 | 18 | 23 |  | |  |  |  |
| bta-miR-874 | CXCL8 | 12 | 21 | | bta-miR-339a | ICAM1 | 19 | 38 |  | |  |  |  |
| bta-miR-95 | CXCL8 | 14 | 16 | | bta-miR-375 | ICAM1 | 15 | 18 |  | |  |  |  |
| bta-miR-1284 | CXCL8 | 15 | 20 | | bta-miR-409a | ICAM1 | 17 | 21 |  | |  |  |  |
| bta-miR-2309 | CXCL8 | 18 | 23 | | bta-miR-760-3p | ICAM1 | 17 | 22 |  | |  |  |  |
| bta-miR-2316 | CXCL8 | 14 | 17 | | bta-miR-874 | ICAM1 | 19 | 41 |  | |  |  |  |
| bta-miR-2329-3p | CXCL8 | 16 | 26 | | bta-miR-1224 | ICAM1 | 16 | 22 |  | |  |  |  |
| bta-miR-2331-5p | CXCL8 | 19 | 25 | | bta-miR-764 | ICAM1 | 17 | 23 |  | |  |  |  |
| bta-miR-2377 | CXCL8 | 15 | 19 | | bta-miR-1282 | ICAM1 | 17 | 19 |  | |  |  |  |
| bta-miR-2382-5p | CXCL8 | 16 | 22 | | bta-miR-1291 | ICAM1 | 19 | 25 |  | |  |  |  |
| bta-miR-2389 | CXCL8 | 16 | 21 | | bta-miR-1301 | ICAM1 | 18 | 24 |  | |  |  |  |
| bta-miR-1584-5p | CXCL8 | 17 | 22 | | bta-miR-1256 | ICAM1 | 17 | 18 |  | |  |  |  |
| bta-miR-2412 | CXCL8 | 13 | 15 | | bta-miR-1296 | ICAM1 | 19 | 26 |  | |  |  |  |
| bta-miR-2415-3p | CXCL8 | 18 | 23 | | bta-miR-2286 | ICAM1 | 17 | 21 |  | |  |  |  |
| bta-miR-2422 | CXCL8 | 19 | 21 | | bta-miR-2287 | ICAM1 | 18 | 22 |  | |  |  |  |
| bta-miR-2448-3p | CXCL8 | 11 | 12 | | bta-miR-2294 | ICAM1 | 15 | 18 |  | |  |  |  |
| bta-miR-2450b | CXCL8 | 17 | 23 | | bta-miR-2296 | ICAM1 | 15 | 19 |  | |  |  |  |
| bta-miR-2451 | CXCL8 | 15 | 21 | | bta-miR-2302 | ICAM1 | 19 | 27 |  | |  |  |  |
| bta-miR-2454-5p | CXCL8 | 20 | 28 | | bta-miR-2306 | ICAM1 | 20 | 47 |  | |  |  |  |
| bta-miR-2457 | CXCL8 | 15 | 21 | | bta-miR-2313-5p | ICAM1 | 15 | 18 |  | |  |  |  |
| bta-miR-1777b | CXCL8 | 17 | 21 | | bta-miR-2313-5p | ICAM1 | 18 | 34 |  | |  |  |  |
| bta-miR-2462 | CXCL8 | 13 | 14 | | bta-miR-2313-3p | ICAM1 | 17 | 25 |  | |  |  |  |
| bta-miR-2467-3p | CXCL8 | 17 | 22 | | bta-miR-2316 | ICAM1 | 16 | 19 |  | |  |  |  |
| bta-miR-2471-3p | CXCL8 | 11 | 12 | | bta-miR-1343-3p | ICAM1 | 16 | 18 |  | |  |  |  |
| bta-miR-2486-5p | CXCL8 | 15 | 18 | | bta-miR-2322-5p | ICAM1 | 15 | 18 |  | |  |  |  |
| bta-miR-424-3p | CXCL8 | 16 | 18 | | bta-miR-2340 | ICAM1 | 16 | 20 |  | |  |  |  |
| bta-miR-542-5p | CXCL8 | 9 | 10 | | bta-miR-2348 | ICAM1 | 21 | 43 |  | |  |  |  |
| bta-miR-2881 | CXCL8 | 16 | 21 | | bta-miR-2349 | ICAM1 | 21 | 30 |  | |  |  |  |
| bta-miR-2885 | CXCL8 | 15 | 20 | | bta-miR-2373-3p | ICAM1 | 18 | 22 |  | |  |  |  |
| bta-miR-2887 | CXCL8 | 17 | 20 | | bta-miR-2376 | ICAM1 | 21 | 29 |  | |  |  |  |
| bta-miR-2892 | CXCL8 | 13 | 20 | | bta-miR-2381 | ICAM1 | 17 | 27 |  | |  |  |  |
| bta-miR-149-5p | CXCL8 | 17 | 22 | | bta-miR-2385-5p | ICAM1 | 19 | 24 |  | |  |  |  |
| bta-miR-149-3p | CXCL8 | 18 | 22 | | bta-miR-2388-5p | ICAM1 | 20 | 23 |  | |  |  |  |
| bta-miR-3141 | CXCL8 | 15 | 21 | | bta-miR-2394 | ICAM1 | 16 | 22 |  | |  |  |  |
| bta-miR-6121-5p | CXCL8 | 17 | 22 | | bta-miR-2399-5p | ICAM1 | 20 | 29 |  | |  |  |  |
| bta-miR-1247-3p | CXCL8 | 17 | 20 | | bta-miR-2406 | ICAM1 | 17 | 23 |  | |  |  |  |
| bta-miR-6529a | CXCL8 | 17 | 19 | | bta-miR-1584-5p | ICAM1 | 17 | 19 |  | |  |  |  |
| bta-miR-7865 | CXCL8 | 13 | 16 | | bta-miR-2412 | ICAM1 | 20 | 24 |  | |  |  |  |
| bta-miR-1842 | CXCL8 | 11 | 12 | | bta-miR-2412 | ICAM1 | 18 | 24 |  | |  |  |  |
| bta-miR-10167-3p | CXCL8 | 18 | 27 | | bta-miR-2413 | ICAM1 | 18 | 23 |  | |  |  |  |
| bta-miR-10172-3p | CXCL8 | 12 | 14 | | bta-miR-2418 | ICAM1 | 16 | 21 |  | |  |  |  |
| bta-miR-10177-5p | CXCL8 | 14 | 16 | | bta-miR-2425-5p | ICAM1 | 12 | 14 |  | |  |  |  |
| bta-miR-10178-5p | CXCL8 | 17 | 21 | | bta-miR-2432 | ICAM1 | 17 | 19 |  | |  |  |  |
| bta-miR-2285ah-5p | CXCL8 | 17 | 22 | | bta-miR-2442 | ICAM1 | 22 | 35 |  | |  |  |  |
| bta-miR-12014 | CXCL8 | 20 | 31 | | bta-miR-2448-3p | ICAM1 | 19 | 24 |  | |  |  |  |
| bta-miR-12026 | CXCL8 | 15 | 17 | | bta-miR-2449 | ICAM1 | 15 | 17 |  | |  |  |  |
| bta-miR-12026 | CXCL8 | 14 | 19 | | bta-miR-2450a | ICAM1 | 19 | 32 |  | |  |  |  |
| bta-miR-12049 | CXCL8 | 15 | 22 | | bta-miR-2454-3p | ICAM1 | 16 | 21 |  | |  |  |  |
| bta-miR-12060 | CXCL8 | 14 | 19 | | bta-miR-2460 | ICAM1 | 18 | 19 |  | |  |  |  |
| bta-miR-18b | ICAM1 | 16 | 18 | | bta-miR-2464-3p | ICAM1 | 16 | 22 |  | |  |  |  |
| bta-miR-18a | ICAM1 | 15 | 18 | | bta-miR-2465 | ICAM1 | 15 | 18 |  | |  |  |  |
| bta-miR-125a | ICAM1 | 16 | 23 | | bta-miR-2467-5p | ICAM1 | 19 | 37 |  | |  |  |  |
| bta-miR-107 | ICAM1 | 17 | 22 | | bta-miR-2467-3p | ICAM1 | 16 | 17 |  | |  |  |  |
| bta-miR-345-5p | ICAM1 | 20 | 43 | | bta-miR-2471-3p | ICAM1 | 16 | 21 |  | |  |  |  |
| bta-miR-92a | ICAM1 | 19 | 25 | | bta-miR-2472 | ICAM1 | 18 | 41 |  | |  |  |  |
| bta-miR-148b | ICAM1 | 13 | 15 | | bta-miR-664a | ICAM1 | 18 | 20 |  | |  |  |  |
| bta-miR-214 | ICAM1 | 17 | 22 | | bta-miR-664a | ICAM1 | 16 | 18 |  | |  |  |  |
| bta-miR-423-3p | ICAM1 | 16 | 21 | | bta-miR-193a | ICAM1 | 15 | 17 |  | |  |  |  |
| bta-miR-455-3p | ICAM1 | 16 | 19 | | bta-miR-2881 | ICAM1 | 16 | 18 |  | |  |  |  |
| bta-miR-24-3p | ICAM1 | 16 | 23 | | bta-miR-2886 | ICAM1 | 11 | 14 |  | |  |  |  |
| bta-miR-7 | ICAM1 | 13 | 14 | | bta-miR-2886 | ICAM1 | 13 | 17 |  | |  |  |  |
| bta-miR-532 | ICAM1 | 18 | 23 | | bta-miR-2887 | ICAM1 | 17 | 28 |  | |  |  |  |
| bta-let-7i | ICAM1 | 16 | 18 | | bta-miR-3602 | ICAM1 | 20 | 41 |  | |  |  |  |
| bta-let-7e | ICAM1 | 16 | 18 | | bta-miR-3141 | ICAM1 | 12 | 14 |  | |  |  |  |
| bta-miR-15a | ICAM1 | 16 | 21 | | bta-miR-2285k | ICAM1 | 10 | 12 |  | |  |  |  |
| bta-miR-497 | ICAM1 | 16 | 21 | | bta-miR-6517 | ICAM1 | 21 | 31 |  | |  |  |  |
| bta-miR-134 | ICAM1 | 18 | 20 | | bta-miR-3957 | ICAM1 | 17 | 20 |  | |  |  |  |
| bta-miR-340 | ICAM1 | 18 | 22 | | bta-miR-6523a | ICAM1 | 18 | 21 |  | |  |  |  |
| bta-miR-370 | ICAM1 | 18 | 22 | | bta-miR-6533 | ICAM1 | 16 | 18 |  | |  |  |  |
| bta-miR-431 | ICAM1 | 21 | 28 | | bta-miR-7860 | ICAM1 | 16 | 21 |  | |  |  |  |
| bta-miR-448 | ICAM1 | 17 | 23 | | bta-miR-7865 | ICAM1 | 16 | 17 |  | |  |  |  |
| bta-miR-490 | ICAM1 | 16 | 22 | | bta-miR-378d | ICAM1 | 13 | 15 |  | |  |  |  |
| bta-miR-760-3p | ICAM1 | 18 | 23 | | bta-miR-3432b | ICAM1 | 19 | 23 |  | |  |  |  |
| bta-miR-767 | ICAM1 | 17 | 20 | | bta-miR-10020 | ICAM1 | 21 | 37 |  | |  |  |  |
| bta-miR-885 | ICAM1 | 17 | 23 | | bta-miR-10163-3p | ICAM1 | 15 | 17 |  | |  |  |  |
| bta-miR-92b | ICAM1 | 17 | 21 | | bta-miR-10168-5p | ICAM1 | 16 | 19 |  | |  |  |  |
| bta-miR-1284 | ICAM1 | 17 | 22 | | bta-miR-10169-3p | ICAM1 | 16 | 18 |  | |  |  |  |
| bta-miR-1835 | ICAM1 | 21 | 43 | | bta-miR-10171-3p | ICAM1 | 20 | 29 |  | |  |  |  |
| bta-miR-2319a | ICAM1 | 17 | 20 | | bta-miR-10175-3p | ICAM1 | 17 | 22 |  | |  |  |  |
| bta-miR-2323 | ICAM1 | 19 | 31 | | bta-miR-194b-3p | ICAM1 | 14 | 15 |  | |  |  |  |
| bta-miR-2331-5p | ICAM1 | 16 | 22 | | bta-miR-668-3p | ICAM1 | 17 | 21 |  | |  |  |  |
| bta-miR-2338 | ICAM1 | 17 | 20 | | bta-miR-10182-5p | ICAM1 | 19 | 30 |  | |  |  |  |
| bta-miR-2343 | ICAM1 | 16 | 19 | | bta-miR-10185-5p | ICAM1 | 20 | 36 |  | |  |  |  |
| bta-miR-2355-3p | ICAM1 | 18 | 23 | | bta-miR-2285ah-5p | ICAM1 | 18 | 24 |  | |  |  |  |
| bta-miR-2357 | ICAM1 | 20 | 29 | | bta-miR-10225a | ICAM1 | 9 | 12 |  | |  |  |  |
| bta-miR-2364 | ICAM1 | 19 | 23 | | bta-miR-11972 | ICAM1 | 19 | 22 |  | |  |  |  |
| bta-miR-2373-3p | ICAM1 | 21 | 44 | | bta-miR-11993 | ICAM1 | 16 | 19 |  | |  |  |  |
| bta-miR-2382-5p | ICAM1 | 19 | 24 | | bta-miR-148d | ICAM1 | 16 | 19 |  | |  |  |  |
| bta-miR-2396 | ICAM1 | 16 | 19 | | bta-miR-12010 | ICAM1 | 19 | 20 |  | |  |  |  |
| bta-miR-2401 | ICAM1 | 16 | 22 | | bta-miR-12019 | ICAM1 | 17 | 18 |  | |  |  |  |
| bta-miR-2403 | ICAM1 | 17 | 23 | | bta-miR-12025 | ICAM1 | 17 | 19 |  | |  |  |  |
| bta-miR-2410 | ICAM1 | 17 | 20 | | bta-miR-12027 | ICAM1 | 19 | 24 |  | |  |  |  |
| bta-miR-1584-3p | ICAM1 | 19 | 24 | | bta-miR-12035 | ICAM1 | 15 | 20 |  | |  |  |  |
| bta-miR-2418 | ICAM1 | 19 | 22 | | bta-miR-194b | ICAM1 | 15 | 17 |  | |  |  |  |
| bta-miR-2422 | ICAM1 | 19 | 25 | | bta-miR-12038 | ICAM1 | 14 | 18 |  | |  |  |  |
| bta-miR-2426 | ICAM1 | 19 | 31 | | bta-miR-12041 | ICAM1 | 15 | 17 |  | |  |  |  |
| bta-miR-2426 | ICAM1 | 14 | 15 | | bta-miR-12057 | ICAM1 | 15 | 21 |  | |  |  |  |
| bta-miR-2439-5p | ICAM1 | 17 | 19 | | bta-miR-145 | IL-4 | 17 | 21 |  | |  |  |  |
| bta-miR-2447 | ICAM1 | 17 | 24 | | bta-miR-139 | IL-4 | 17 | 22 |  | |  |  |  |
| bta-miR-2449 | ICAM1 | 14 | 19 | | bta-miR-138 | IL-4 | 16 | 18 |  | |  |  |  |
| bta-miR-2450b | ICAM1 | 22 | 38 | | bta-miR-210 | IL-4 | 14 | 18 |  | |  |  |  |
| bta-miR-2450a | ICAM1 | 21 | 26 | | bta-miR-423-5p | IL-4 | 13 | 17 |  | |  |  |  |
| bta-miR-2461-3p | ICAM1 | 19 | 56 | | bta-miR-423-3p | IL-4 | 15 | 19 |  | |  |  |  |
| bta-miR-2463 | ICAM1 | 13 | 15 | | bta-miR-455-3p | IL-4 | 10 | 12 |  | |  |  |  |
| bta-miR-2466-5p | ICAM1 | 22 | 63 | | bta-miR-10b | IL-4 | 11 | 12 |  | |  |  |  |
| bta-miR-2469 | ICAM1 | 16 | 18 | | bta-miR-24-3p | IL-4 | 18 | 21 |  | |  |  |  |
| bta-miR-669 | ICAM1 | 21 | 31 | | bta-miR-23b-5p | IL-4 | 11 | 14 |  | |  |  |  |
| bta-miR-2893 | ICAM1 | 16 | 20 | | bta-miR-154a | IL-4 | 13 | 17 |  | |  |  |  |
| bta-miR-3141 | ICAM1 | 17 | 23 | | bta-miR-196a | IL-4 | 15 | 17 |  | |  |  |  |
| bta-miR-3957 | ICAM1 | 17 | 21 | | bta-miR-381 | IL-4 | 16 | 18 |  | |  |  |  |
| bta-miR-6525 | ICAM1 | 13 | 15 | | bta-miR-490 | IL-4 | 16 | 23 |  | |  |  |  |
| bta-miR-503-5p | ICAM1 | 12 | 13 | | bta-miR-493 | IL-4 | 16 | 18 |  | |  |  |  |
| bta-miR-6534 | ICAM1 | 14 | 17 | | bta-miR-584 | IL-4 | 13 | 15 |  | |  |  |  |
| bta-miR-7691 | ICAM1 | 12 | 15 | | bta-miR-1301 | IL-4 | 18 | 22 |  | |  |  |  |
| bta-miR-7691 | ICAM1 | 11 | 14 | | bta-miR-2284s | IL-4 | 17 | 22 |  | |  |  |  |
| bta-miR-7862 | ICAM1 | 19 | 38 | | bta-miR-2305 | IL-4 | 14 | 17 |  | |  |  |  |
| bta-miR-2284ac | ICAM1 | 19 | 24 | | bta-miR-2308 | IL-4 | 17 | 20 |  | |  |  |  |
| bta-miR-7865 | ICAM1 | 18 | 20 | | bta-miR-2309 | IL-4 | 14 | 17 |  | |  |  |  |
| bta-miR-2450d | ICAM1 | 20 | 26 | | bta-miR-2331-5p | IL-4 | 20 | 52 |  | |  |  |  |
| bta-miR-10173-5p | ICAM1 | 16 | 19 | | bta-miR-2284a | IL-4 | 13 | 16 |  | |  |  |  |
| bta-miR-2285as | ICAM1 | 11 | 12 | | bta-miR-2378 | IL-4 | 16 | 18 |  | |  |  |  |
| bta-miR-2285ay | ICAM1 | 11 | 12 | | bta-miR-2381 | IL-4 | 17 | 21 |  | |  |  |  |
| bta-miR-9851 | ICAM1 | 16 | 21 | | bta-miR-2385-5p | IL-4 | 18 | 19 |  | |  |  |  |
| bta-miR-2285bc | ICAM1 | 13 | 15 | | bta-miR-2284v | IL-4 | 14 | 15 |  | |  |  |  |
| bta-miR-11987 | ICAM1 | 16 | 22 | | bta-miR-2392 | IL-4 | 19 | 22 |  | |  |  |  |
| bta-miR-2285bj | ICAM1 | 11 | 12 | | bta-miR-2395 | IL-4 | 14 | 19 |  | |  |  |  |
| bta-miR-7180 | ICAM1 | 21 | 37 | | bta-miR-2396 | IL-4 | 14 | 15 |  | |  |  |  |
| bta-miR-11990 | ICAM1 | 12 | 14 | | bta-miR-2400 | IL-4 | 14 | 18 |  | |  |  |  |
| bta-miR-11986c | ICAM1 | 14 | 16 | | bta-miR-2401 | IL-4 | 15 | 17 |  | |  |  |  |
| bta-miR-11994 | ICAM1 | 18 | 24 | | bta-miR-2410 | IL-4 | 18 | 25 |  | |  |  |  |
| bta-miR-12011 | ICAM1 | 12 | 15 | | bta-miR-1584-5p | IL-4 | 16 | 20 |  | |  |  |  |
| bta-miR-2285cs | ICAM1 | 11 | 12 | | bta-miR-1584-5p | IL-4 | 16 | 17 |  | |  |  |  |
| bta-miR-2285ct | ICAM1 | 13 | 14 | | bta-miR-2411-5p | IL-4 | 17 | 22 |  | |  |  |  |
| bta-miR-12019 | ICAM1 | 17 | 19 | | bta-miR-1843 | IL-4 | 20 | 32 |  | |  |  |  |
| bta-miR-12022 | ICAM1 | 16 | 22 | | bta-miR-2450a | IL-4 | 17 | 21 |  | |  |  |  |
| bta-miR-12025 | ICAM1 | 15 | 20 | | bta-miR-1777a | IL-4 | 18 | 27 |  | |  |  |  |
| bta-miR-12039 | ICAM1 | 22 | 39 | | bta-miR-2455 | IL-4 | 11 | 16 |  | |  |  |  |
| bta-miR-12047 | ICAM1 | 18 | 40 | | bta-miR-2456 | IL-4 | 20 | 27 |  | |  |  |  |
| bta-miR-12048 | ICAM1 | 17 | 19 | | bta-miR-2457 | IL-4 | 16 | 23 |  | |  |  |  |
| bta-miR-12052 | ICAM1 | 20 | 25 | | bta-miR-1777b | IL-4 | 15 | 16 |  | |  |  |  |
| bta-miR-12054 | ICAM1 | 12 | 13 | | bta-miR-2474 | IL-4 | 12 | 17 |  | |  |  |  |
| bta-miR-150 | CSF2 | 17 | 24 | | bta-miR-2881 | IL-4 | 16 | 21 |  | |  |  |  |
| bta-miR-133a | CSF2 | 14 | 17 | | bta-miR-2899 | IL-4 | 16 | 19 |  | |  |  |  |
| bta-miR-133b | CSF2 | 14 | 17 | | bta-miR-2900 | IL-4 | 14 | 16 |  | |  |  |  |
| bta-miR-326 | CSF2 | 16 | 24 | | bta-miR-3602 | IL-4 | 18 | 20 |  | |  |  |  |
| bta-miR-328 | CSF2 | 21 | 28 | | bta-miR-154b | IL-4 | 15 | 20 |  | |  |  |  |
| bta-miR-412 | CSF2 | 19 | 25 | | bta-miR-6527 | IL-4 | 16 | 20 |  | |  |  |  |
| bta-miR-760-3p | CSF2 | 16 | 22 | | bta-miR-7691 | IL-4 | 11 | 13 |  | |  |  |  |
| bta-miR-769 | CSF2 | 15 | 23 | | bta-miR-4523 | IL-4 | 15 | 21 |  | |  |  |  |
| bta-miR-1291 | CSF2 | 18 | 23 | | bta-miR-10171-3p | IL-4 | 18 | 26 |  | |  |  |  |
| bta-miR-2286 | CSF2 | 16 | 19 | | bta-miR-10179-5p | IL-4 | 20 | 28 |  | |  |  |  |
| bta-miR-2295 | CSF2 | 16 | 22 | | bta-miR-2285ak-5p | IL-4 | 15 | 18 |  | |  |  |  |
| bta-miR-2302 | CSF2 | 16 | 19 | | bta-miR-2285al-5p | IL-4 | 15 | 18 |  | |  |  |  |
| bta-miR-2319a | CSF2 | 15 | 18 | | bta-miR-2285au | IL-4 | 15 | 19 |  | |  |  |  |
| bta-miR-2320-3p | CSF2 | 12 | 15 | | bta-miR-11981 | IL-4 | 18 | 27 |  | |  |  |  |
| bta-miR-2340 | CSF2 | 17 | 19 | | bta-miR-11989 | IL-4 | 20 | 37 |  | |  |  |  |
| bta-miR-2364 | CSF2 | 17 | 22 | | bta-miR-12003 | IL-4 | 14 | 16 |  | |  |  |  |
| bta-miR-2368-3p | CSF2 | 17 | 35 | | bta-miR-2285cr | IL-4 | 13 | 15 |  | |  |  |  |
| bta-miR-2373-5p | CSF2 | 20 | 55 | | bta-miR-2285cr | IL-4 | 13 | 16 |  | |  |  |  |
| bta-miR-2375 | CSF2 | 17 | 19 | | bta-miR-12026 | IL-4 | 14 | 19 |  | |  |  |  |
| bta-miR-2425-5p | CSF2 | 13 | 18 | | bta-miR-12033 | IL-4 | 11 | 13 |  | |  |  |  |
| bta-miR-2425-3p | CSF2 | 16 | 22 | | bta-miR-12049 | IL-4 | 14 | 20 |  | |  |  |  |
| bta-miR-2436-3p | CSF2 | 17 | 43 | | bta-miR-12053 | IL-4 | 16 | 20 |  | |  |  |  |
| bta-miR-2443 | CSF2 | 18 | 24 | | bta-miR-12056 | IL-4 | 16 | 19 |  | |  |  |  |
| bta-miR-2473 | CSF2 | 15 | 31 | | bta-miR-12063 | IL-4 | 10 | 12 |  | |  |  |  |
| bta-miR-2488 | CSF2 | 10 | 11 | | bta-miR-21-3p | CSF2 | 13 | 16 |  | |  |  |  |
| bta-miR-2882 | CSF2 | 15 | 23 | | bta-miR-10a | CSF2 | 11 | 13 |  | |  |  |  |
| bta-miR-2894 | CSF2 | 13 | 16 | | bta-miR-93 | CSF2 | 16 | 17 |  | |  |  |  |
| bta-miR-2897 | CSF2 | 11 | 12 | | bta-miR-10b | CSF2 | 11 | 13 |  | |  |  |  |
| bta-miR-6521 | CSF2 | 16 | 23 | | bta-miR-23b-5p | CSF2 | 11 | 12 |  | |  |  |  |
| bta-miR-3956 | CSF2 | 20 | 25 | | bta-miR-23b-3p | CSF2 | 15 | 18 |  | |  |  |  |
| bta-miR-7862 | CSF2 | 14 | 19 | | bta-miR-105b | CSF2 | 20 | 27 |  | |  |  |  |
| bta-miR-1842 | CSF2 | 17 | 22 | | bta-miR-105a | CSF2 | 21 | 30 |  | |  |  |  |
| bta-miR-10020 | CSF2 | 17 | 22 | | bta-miR-146b | CSF2 | 20 | 26 |  | |  |  |  |
| bta-miR-10164-3p | CSF2 | 18 | 29 | | bta-miR-146a | CSF2 | 20 | 27 |  | |  |  |  |
| bta-miR-10170-5p | CSF2 | 17 | 21 | | bta-miR-216b | CSF2 | 10 | 12 |  | |  |  |  |
| bta-miR-10180-3p | CSF2 | 15 | 21 | | bta-miR-28 | CSF2 | 19 | 24 |  | |  |  |  |
| bta-miR-10182-5p | CSF2 | 18 | 23 | | bta-miR-382 | CSF2 | 15 | 17 |  | |  |  |  |
| bta-miR-11973 | CSF2 | 20 | 36 | | bta-miR-541 | CSF2 | 16 | 31 |  | |  |  |  |
| bta-miR-11979 | CSF2 | 17 | 21 | | bta-miR-671 | CSF2 | 14 | 17 |  | |  |  |  |
| bta-miR-11982 | CSF2 | 17 | 20 | | bta-miR-1296 | CSF2 | 18 | 26 |  | |  |  |  |
| bta-miR-11998 | CSF2 | 12 | 13 | | bta-miR-2287 | CSF2 | 15 | 20 |  | |  |  |  |
| bta-miR-12004 | CSF2 | 17 | 24 | | bta-miR-2294 | CSF2 | 19 | 23 |  | |  |  |  |
| bta-miR-12007 | CSF2 | 18 | 23 | | bta-miR-2294 | CSF2 | 18 | 22 |  | |  |  |  |
| bta-miR-6775 | CSF2 | 20 | 45 | | bta-miR-2305 | CSF2 | 15 | 21 |  | |  |  |  |
| bta-miR-12015 | CSF2 | 15 | 19 | | bta-miR-2314 | CSF2 | 16 | 18 |  | |  |  |  |
| bta-miR-12049 | CSF2 | 17 | 19 | | bta-miR-2324 | CSF2 | 21 | 40 |  | |  |  |  |
| bta-miR-12051 | CSF2 | 17 | 20 | | bta-miR-2324 | CSF2 | 20 | 25 |  | |  |  |  |
| bta-miR-12054 | CSF2 | 15 | 20 | | bta-miR-2333 | CSF2 | 17 | 21 |  | |  |  |  |
| bta-miR-12059 | CSF2 | 16 | 22 | | bta-miR-2348 | CSF2 | 17 | 20 |  | |  |  |  |
| bta-miR-125a | CD14 | 15 | 23 | | bta-miR-2368-5p | CSF2 | 19 | 31 |  | |  |  |  |
| bta-miR-127 | CD14 | 16 | 19 | | bta-miR-2368-5p | CSF2 | 12 | 17 |  | |  |  |  |
| bta-miR-193a-5p | CD14 | 15 | 18 | | bta-miR-2369 | CSF2 | 17 | 21 |  | |  |  |  |
| bta-miR-345-3p | CD14 | 17 | 21 | | bta-miR-2377 | CSF2 | 16 | 19 |  | |  |  |  |
| bta-miR-138 | CD14 | 14 | 16 | | bta-miR-2388-3p | CSF2 | 15 | 19 |  | |  |  |  |
| bta-miR-455-5p | CD14 | 10 | 11 | | bta-miR-2389 | CSF2 | 15 | 22 |  | |  |  |  |
| bta-miR-93 | CD14 | 13 | 14 | | bta-miR-2407 | CSF2 | 17 | 26 |  | |  |  |  |
| bta-miR-24-3p | CD14 | 15 | 19 | | bta-miR-2411-5p | CSF2 | 17 | 22 |  | |  |  |  |
| bta-let-7a-5p | CD14 | 17 | 23 | | bta-miR-2412 | CSF2 | 16 | 19 |  | |  |  |  |
| bta-let-7e | CD14 | 14 | 16 | | bta-miR-2415-3p | CSF2 | 10 | 14 |  | |  |  |  |
| bta-let-7e | CD14 | 18 | 22 | | bta-miR-2416 | CSF2 | 16 | 19 |  | |  |  |  |
| bta-miR-106b | CD14 | 14 | 15 | | bta-miR-2419-3p | CSF2 | 11 | 12 |  | |  |  |  |
| bta-miR-134 | CD14 | 17 | 21 | | bta-miR-2426 | CSF2 | 13 | 16 |  | |  |  |  |
| bta-miR-328 | CD14 | 18 | 22 | | bta-miR-2430 | CSF2 | 21 | 25 |  | |  |  |  |
| bta-miR-329b | CD14 | 17 | 22 | | bta-miR-2432 | CSF2 | 16 | 20 |  | |  |  |  |
| bta-miR-339a | CD14 | 16 | 24 | | bta-miR-2433 | CSF2 | 20 | 24 |  | |  |  |  |
| bta-miR-370 | CD14 | 20 | 61 | | bta-miR-2449 | CSF2 | 13 | 15 |  | |  |  |  |
| bta-miR-429 | CD14 | 14 | 18 | | bta-miR-2450a | CSF2 | 14 | 17 |  | |  |  |  |
| bta-miR-431 | CD14 | 17 | 22 | | bta-miR-2451 | CSF2 | 16 | 19 |  | |  |  |  |
| bta-miR-483 | CD14 | 17 | 23 | | bta-miR-1777a | CSF2 | 20 | 26 |  | |  |  |  |
| bta-miR-543 | CD14 | 16 | 19 | | bta-miR-1777b | CSF2 | 18 | 22 |  | |  |  |  |
| bta-miR-584 | CD14 | 18 | 25 | | bta-miR-2464-5p | CSF2 | 13 | 15 |  | |  |  |  |
| bta-miR-654 | CD14 | 15 | 18 | | bta-miR-2467-3p | CSF2 | 18 | 21 |  | |  |  |  |
| bta-miR-671 | CD14 | 15 | 18 | | bta-miR-2472 | CSF2 | 20 | 31 |  | |  |  |  |
| bta-miR-744 | CD14 | 16 | 24 | | bta-miR-2486-5p | CSF2 | 16 | 23 |  | |  |  |  |
| bta-miR-1298 | CD14 | 19 | 24 | | bta-miR-669 | CSF2 | 15 | 18 |  | |  |  |  |
| bta-miR-2288 | CD14 | 16 | 20 | | bta-miR-669 | CSF2 | 14 | 16 |  | |  |  |  |
| bta-miR-2294 | CD14 | 17 | 19 | | bta-miR-2893 | CSF2 | 18 | 32 |  | |  |  |  |
| bta-miR-2295 | CD14 | 15 | 19 | | bta-miR-2898 | CSF2 | 13 | 17 |  | |  |  |  |
| bta-miR-2302 | CD14 | 16 | 20 | | bta-miR-2902 | CSF2 | 16 | 21 |  | |  |  |  |
| bta-miR-2305 | CD14 | 17 | 21 | | bta-miR-6523a | CSF2 | 14 | 19 |  | |  |  |  |
| bta-miR-2315 | CD14 | 13 | 14 | | bta-miR-6525 | CSF2 | 12 | 18 |  | |  |  |  |
| bta-miR-2315 | CD14 | 13 | 14 | | bta-miR-6535 | CSF2 | 18 | 24 |  | |  |  |  |
| bta-miR-2317 | CD14 | 17 | 22 | | bta-miR-7860 | CSF2 | 15 | 18 |  | |  |  |  |
| bta-miR-1343-3p | CD14 | 17 | 23 | | bta-miR-8549 | CSF2 | 11 | 13 |  | |  |  |  |
| bta-miR-2324 | CD14 | 18 | 25 | | bta-miR-4449 | CSF2 | 16 | 21 |  | |  |  |  |
| bta-miR-2324 | CD14 | 18 | 23 | | bta-miR-10020 | CSF2 | 16 | 18 |  | |  |  |  |
| bta-miR-2326 | CD14 | 14 | 17 | | bta-miR-10161-5p | CSF2 | 11 | 12 |  | |  |  |  |
| bta-miR-2339 | CD14 | 13 | 19 | | bta-miR-10174-5p | CSF2 | 11 | 12 |  | |  |  |  |
| bta-miR-2340 | CD14 | 18 | 21 | | bta-miR-10174-3p | CSF2 | 15 | 18 |  | |  |  |  |
| bta-miR-2343 | CD14 | 15 | 20 | | bta-miR-10178-5p | CSF2 | 17 | 23 |  | |  |  |  |
| bta-miR-2343 | CD14 | 14 | 16 | | bta-miR-10185-5p | CSF2 | 17 | 21 |  | |  |  |  |
| bta-miR-2344 | CD14 | 18 | 22 | | bta-miR-2285ah-5p | CSF2 | 17 | 20 |  | |  |  |  |
| bta-miR-2356 | CD14 | 20 | 23 | | bta-miR-2285ak-5p | CSF2 | 19 | 23 |  | |  |  |  |
| bta-miR-2369 | CD14 | 15 | 18 | | bta-miR-12011 | CSF2 | 14 | 16 |  | |  |  |  |
| bta-miR-2371 | CD14 | 13 | 14 | | bta-miR-12019 | CSF2 | 18 | 33 |  | |  |  |  |
| bta-miR-2377 | CD14 | 17 | 21 | | bta-miR-12030 | CSF2 | 14 | 17 |  | |  |  |  |
| bta-miR-2378 | CD14 | 18 | 27 | | bta-miR-12033 | CSF2 | 14 | 16 |  | |  |  |  |
| bta-miR-2381 | CD14 | 19 | 25 | | bta-miR-12038 | CSF2 | 17 | 20 |  | |  |  |  |
| bta-miR-2392 | CD14 | 17 | 24 | | bta-miR-12039 | CSF2 | 12 | 16 |  | |  |  |  |
| bta-miR-2392 | CD14 | 17 | 18 | | bta-miR-12046 | CSF2 | 15 | 31 |  | |  |  |  |
| bta-miR-2403 | CD14 | 17 | 19 | | bta-miR-12058 | CSF2 | 14 | 15 |  | |  |  |  |
| bta-miR-2406 | CD14 | 17 | 27 | | bta-miR-12062 | CSF2 | 16 | 20 |  | |  |  |  |
| bta-miR-1584-3p | CD14 | 14 | 17 | | bta-miR-12062 | CSF2 | 15 | 19 |  | |  |  |  |
| bta-miR-2417 | CD14 | 13 | 16 | | bta-miR-151-5p | CD14 | 16 | 19 |  | |  |  |  |
| bta-miR-320b | CD14 | 17 | 30 | | bta-miR-320a | CD14 | 15 | 18 |  | |  |  |  |
| bta-miR-2439-3p | CD14 | 18 | 20 | | bta-miR-320a | CD14 | 15 | 18 |  | |  |  |  |
| bta-miR-2284h-5p | CD14 | 16 | 20 | | bta-miR-125b | CD14 | 15 | 21 |  | |  |  |  |
| bta-miR-2450c | CD14 | 16 | 17 | | bta-miR-145 | CD14 | 15 | 19 |  | |  |  |  |
| bta-miR-2451 | CD14 | 19 | 24 | | bta-miR-138 | CD14 | 18 | 22 |  | |  |  |  |
| bta-miR-1777a | CD14 | 17 | 19 | | bta-miR-24-3p | CD14 | 16 | 18 |  | |  |  |  |
| bta-miR-339b | CD14 | 15 | 22 | | bta-miR-532 | CD14 | 16 | 20 |  | |  |  |  |
| bta-miR-339b | CD14 | 17 | 24 | | bta-miR-23b-5p | CD14 | 17 | 21 |  | |  |  |  |
| bta-miR-2460 | CD14 | 16 | 22 | | bta-miR-331-3p | CD14 | 17 | 23 |  | |  |  |  |
| bta-miR-2461-5p | CD14 | 17 | 24 | | bta-miR-365-5p | CD14 | 14 | 16 |  | |  |  |  |
| bta-miR-2461-3p | CD14 | 20 | 23 | | bta-miR-184 | CD14 | 15 | 16 |  | |  |  |  |
| bta-miR-2465 | CD14 | 19 | 20 | | bta-miR-188 | CD14 | 16 | 18 |  | |  |  |  |
| bta-miR-2469 | CD14 | 18 | 23 | | bta-miR-206 | CD14 | 17 | 23 |  | |  |  |  |
| bta-miR-2483-3p | CD14 | 14 | 16 | | bta-miR-375 | CD14 | 18 | 22 |  | |  |  |  |
| bta-miR-669 | CD14 | 20 | 24 | | bta-miR-409a | CD14 | 12 | 14 |  | |  |  |  |
| bta-miR-2881 | CD14 | 12 | 13 | | bta-miR-431 | CD14 | 18 | 20 |  | |  |  |  |
| bta-miR-2882 | CD14 | 17 | 42 | | bta-miR-432 | CD14 | 19 | 21 |  | |  |  |  |
| bta-miR-2899 | CD14 | 14 | 18 | | bta-miR-453 | CD14 | 22 | 58 |  | |  |  |  |
| bta-miR-2904 | CD14 | 16 | 22 | | bta-miR-502a | CD14 | 16 | 19 |  | |  |  |  |
| bta-miR-148c | CD14 | 13 | 16 | | bta-miR-541 | CD14 | 17 | 21 |  | |  |  |  |
| bta-miR-149-3p | CD14 | 18 | 23 | | bta-miR-760-3p | CD14 | 18 | 24 |  | |  |  |  |
| bta-miR-149-3p | CD14 | 20 | 29 | | bta-miR-760-3p | CD14 | 17 | 19 |  | |  |  |  |
| bta-miR-4286 | CD14 | 10 | 11 | | bta-miR-769 | CD14 | 17 | 19 |  | |  |  |  |
| bta-miR-6120-5p | CD14 | 13 | 17 | | bta-miR-873 | CD14 | 19 | 23 |  | |  |  |  |
| bta-miR-378b | CD14 | 17 | 24 | | bta-miR-1301 | CD14 | 16 | 21 |  | |  |  |  |
| bta-miR-6523a | CD14 | 16 | 17 | | bta-miR-1835 | CD14 | 17 | 20 |  | |  |  |  |
| bta-miR-6525 | CD14 | 18 | 22 | | bta-miR-1307 | CD14 | 19 | 24 |  | |  |  |  |
| bta-miR-3660 | CD14 | 14 | 16 | | bta-miR-1287 | CD14 | 16 | 19 |  | |  |  |  |
| bta-miR-6530 | CD14 | 14 | 16 | | bta-miR-2287 | CD14 | 15 | 17 |  | |  |  |  |
| bta-miR-6531 | CD14 | 18 | 23 | | bta-miR-2295 | CD14 | 16 | 21 |  | |  |  |  |
| bta-miR-6535 | CD14 | 17 | 19 | | bta-miR-2299-3p | CD14 | 16 | 24 |  | |  |  |  |
| bta-miR-7865 | CD14 | 17 | 19 | | bta-miR-2302 | CD14 | 17 | 21 |  | |  |  |  |
| bta-miR-8549 | CD14 | 14 | 19 | | bta-miR-2309 | CD14 | 18 | 20 |  | |  |  |  |
| bta-miR-4657 | CD14 | 16 | 18 | | bta-miR-2309 | CD14 | 19 | 22 |  | |  |  |  |
| bta-miR-4523 | CD14 | 16 | 22 | | bta-miR-2313-5p | CD14 | 16 | 19 |  | |  |  |  |
| bta-miR-4523 | CD14 | 15 | 18 | | bta-miR-2284d | CD14 | 15 | 17 |  | |  |  |  |
| bta-miR-10165-5p | CD14 | 15 | 20 | | bta-miR-2316 | CD14 | 19 | 24 |  | |  |  |  |
| bta-miR-10166-5p | CD14 | 19 | 23 | | bta-miR-2316 | CD14 | 20 | 48 |  | |  |  |  |
| bta-miR-10168-5p | CD14 | 17 | 22 | | bta-miR-2320-5p | CD14 | 19 | 34 |  | |  |  |  |
| bta-miR-10172-3p | CD14 | 12 | 17 | | bta-miR-2330-3p | CD14 | 15 | 18 |  | |  |  |  |
| bta-miR-10173-5p | CD14 | 18 | 23 | | bta-miR-2341 | CD14 | 13 | 14 |  | |  |  |  |
| bta-miR-10179-5p | CD14 | 19 | 30 | | bta-miR-2355-5p | CD14 | 13 | 15 |  | |  |  |  |
| bta-miR-2285ah-5p | CD14 | 17 | 18 | | bta-miR-2356 | CD14 | 19 | 23 |  | |  |  |  |
| bta-miR-10225b | CD14 | 10 | 11 | | bta-miR-2360 | CD14 | 18 | 24 |  | |  |  |  |
| bta-miR-11972 | CD14 | 17 | 20 | | bta-miR-2374 | CD14 | 19 | 25 |  | |  |  |  |
| bta-miR-11982 | CD14 | 20 | 37 | | bta-miR-2375 | CD14 | 16 | 19 |  | |  |  |  |
| bta-miR-11984 | CD14 | 19 | 34 | | bta-miR-2382-5p | CD14 | 17 | 20 |  | |  |  |  |
| bta-miR-12004 | CD14 | 20 | 41 | | bta-miR-2397-5p | CD14 | 15 | 20 |  | |  |  |  |
| bta-miR-12011 | CD14 | 13 | 17 | | bta-miR-2398 | CD14 | 14 | 15 |  | |  |  |  |
| bta-miR-2285cs | CD14 | 13 | 15 | | bta-miR-449d | CD14 | 14 | 16 |  | |  |  |  |
| bta-miR-12025 | CD14 | 17 | 21 | | bta-miR-2407 | CD14 | 20 | 29 |  | |  |  |  |
| bta-miR-12034 | CD14 | 17 | 28 | | bta-miR-2416 | CD14 | 16 | 18 |  | |  |  |  |
| bta-miR-194b | CD14 | 15 | 17 | | bta-miR-2418 | CD14 | 18 | 26 |  | |  |  |  |
| bta-miR-12045 | CD14 | 19 | 34 | | bta-miR-2422 | CD14 | 19 | 25 |  | |  |  |  |
| bta-miR-2285di | CD14 | 15 | 18 | | bta-miR-320b | CD14 | 17 | 20 |  | |  |  |  |
| bta-miR-12049 | CD14 | 18 | 31 | | bta-miR-2430 | CD14 | 21 | 30 |  | |  |  |  |
| bta-miR-12051 | CD14 | 17 | 19 | | bta-miR-2438 | CD14 | 19 | 45 |  | |  |  |  |
| bta-miR-12056 | CD14 | 16 | 18 | | bta-miR-2439-5p | CD14 | 17 | 19 |  | |  |  |  |
| bta-miR-12061 | CD14 | 17 | 29 | | bta-miR-2443 | CD14 | 20 | 24 |  | |  |  |  |
| bta-miR-30d | TLR4 | 17 | 21 | | bta-miR-2448-5p | CD14 | 13 | 16 |  | |  |  |  |
| bta-miR-128 | TLR4 | 17 | 21 | | bta-miR-2448-3p | CD14 | 17 | 19 |  | |  |  |  |
| bta-miR-15b | TLR4 | 13 | 17 | | bta-miR-2450c | CD14 | 19 | 38 |  | |  |  |  |
| bta-miR-455-3p | TLR4 | 13 | 16 | | bta-miR-2450b | CD14 | 20 | 25 |  | |  |  |  |
| bta-miR-30a-5p | TLR4 | 17 | 21 | | bta-miR-2450a | CD14 | 18 | 23 |  | |  |  |  |
| bta-let-7e | TLR4 | 17 | 21 | | bta-miR-2450a | CD14 | 14 | 16 |  | |  |  |  |
| bta-miR-105b | TLR4 | 18 | 23 | | bta-miR-2451 | CD14 | 18 | 26 |  | |  |  |  |
| bta-miR-339a | TLR4 | 16 | 17 | | bta-miR-2454-3p | CD14 | 17 | 28 |  | |  |  |  |
| bta-miR-449a | TLR4 | 15 | 21 | | bta-miR-2455 | CD14 | 21 | 25 |  | |  |  |  |
| bta-miR-485 | TLR4 | 11 | 14 | | bta-miR-2458 | CD14 | 18 | 21 |  | |  |  |  |
| bta-miR-490 | TLR4 | 15 | 23 | | bta-miR-2460 | CD14 | 18 | 30 |  | |  |  |  |
| bta-miR-502b | TLR4 | 15 | 18 | | bta-miR-1777b | CD14 | 16 | 21 |  | |  |  |  |
| bta-miR-502b | TLR4 | 13 | 14 | | bta-miR-2465 | CD14 | 17 | 23 |  | |  |  |  |
| bta-miR-1224 | TLR4 | 18 | 21 | | bta-miR-2466-5p | CD14 | 21 | 28 |  | |  |  |  |
| bta-miR-2294 | TLR4 | 19 | 27 | | bta-miR-2466-5p | CD14 | 18 | 23 |  | |  |  |  |
| bta-miR-2306 | TLR4 | 17 | 24 | | bta-miR-542-5p | CD14 | 16 | 20 |  | |  |  |  |
| bta-miR-2308 | TLR4 | 15 | 22 | | bta-miR-2885 | CD14 | 17 | 25 |  | |  |  |  |
| bta-miR-2284t-5p | TLR4 | 15 | 17 | | bta-miR-2893 | CD14 | 18 | 24 |  | |  |  |  |
| bta-miR-2328-3p | TLR4 | 21 | 27 | | bta-miR-2894 | CD14 | 14 | 24 |  | |  |  |  |
| bta-miR-2344 | TLR4 | 15 | 24 | | bta-miR-2899 | CD14 | 16 | 24 |  | |  |  |  |
| bta-miR-2356 | TLR4 | 20 | 35 | | bta-miR-3602 | CD14 | 14 | 18 |  | |  |  |  |
| bta-miR-2374 | TLR4 | 16 | 19 | | bta-miR-149-5p | CD14 | 18 | 21 |  | |  |  |  |
| bta-miR-2422 | TLR4 | 16 | 22 | | bta-miR-574 | CD14 | 21 | 26 |  | |  |  |  |
| bta-miR-2425-3p | TLR4 | 12 | 19 | | bta-miR-574 | CD14 | 16 | 20 |  | |  |  |  |
| bta-miR-2448-3p | TLR4 | 14 | 21 | | bta-miR-1247-3p | CD14 | 18 | 25 |  | |  |  |  |
| bta-miR-2460 | TLR4 | 18 | 20 | | bta-miR-503-5p | CD14 | 14 | 19 |  | |  |  |  |
| bta-miR-2461-5p | TLR4 | 18 | 22 | | bta-miR-6529a | CD14 | 19 | 26 |  | |  |  |  |
| bta-miR-2464-3p | TLR4 | 18 | 24 | | bta-miR-6530 | CD14 | 17 | 20 |  | |  |  |  |
| bta-miR-2466-5p | TLR4 | 20 | 26 | | bta-miR-2285z | CD14 | 19 | 33 |  | |  |  |  |
| bta-miR-669 | TLR4 | 18 | 20 | | bta-miR-7865 | CD14 | 14 | 16 |  | |  |  |  |
| bta-miR-26c | TLR4 | 18 | 24 | | bta-miR-4449 | CD14 | 17 | 28 |  | |  |  |  |
| bta-miR-3154 | TLR4 | 16 | 20 | | bta-miR-2450d | CD14 | 20 | 25 |  | |  |  |  |
| bta-miR-6525 | TLR4 | 15 | 21 | | bta-miR-10174-5p | CD14 | 17 | 21 |  | |  |  |  |
| bta-miR-6528 | TLR4 | 16 | 21 | | bta-miR-10178-5p | CD14 | 18 | 41 |  | |  |  |  |
| bta-miR-7857-5p | TLR4 | 16 | 18 | | bta-miR-10180-3p | CD14 | 17 | 21 |  | |  |  |  |
| bta-miR-10179-5p | TLR4 | 20 | 25 | | bta-miR-10180-3p | CD14 | 16 | 20 |  | |  |  |  |
| bta-miR-2285as | TLR4 | 16 | 17 | | bta-miR-10183-5p | CD14 | 17 | 23 |  | |  |  |  |
| bta-miR-2285bb | TLR4 | 12 | 14 | | bta-miR-10185-5p | CD14 | 17 | 19 |  | |  |  |  |
| bta-miR-2285by | TLR4 | 18 | 20 | | bta-miR-11975 | CD14 | 18 | 22 |  | |  |  |  |
| bta-miR-11994 | TLR4 | 21 | 34 | | bta-miR-11976 | CD14 | 19 | 23 |  | |  |  |  |
| bta-miR-11997 | TLR4 | 16 | 20 | | bta-miR-2285aq | CD14 | 12 | 14 |  | |  |  |  |
| bta-miR-12010 | TLR4 | 18 | 24 | | bta-miR-2285bi | CD14 | 12 | 14 |  | |  |  |  |
| bta-miR-12014 | TLR4 | 18 | 26 | | bta-miR-11994 | CD14 | 16 | 19 |  | |  |  |  |
| bta-miR-20a | IL-10 | 19 | 33 | | bta-miR-12003 | CD14 | 19 | 25 |  | |  |  |  |
| bta-miR-106a | IL-10 | 19 | 32 | | bta-miR-12003 | CD14 | 16 | 22 |  | |  |  |  |
| bta-miR-139 | IL-10 | 18 | 19 | | bta-miR-12004 | CD14 | 19 | 21 |  | |  |  |  |
| bta-miR-193a-5p | IL-10 | 16 | 18 | | bta-miR-12004 | CD14 | 16 | 19 |  | |  |  |  |
| bta-miR-17-5p | IL-10 | 19 | 33 | | bta-miR-12010 | CD14 | 19 | 22 |  | |  |  |  |
| bta-miR-214 | IL-10 | 18 | 24 | | bta-miR-12010 | CD14 | 18 | 20 |  | |  |  |  |
| bta-miR-365-5p | IL-10 | 19 | 45 | | bta-miR-12011 | CD14 | 13 | 16 |  | |  |  |  |
| bta-miR-183 | IL-10 | 18 | 23 | | bta-miR-12012 | CD14 | 17 | 19 |  | |  |  |  |
| bta-miR-323 | IL-10 | 15 | 19 | | bta-miR-12013 | CD14 | 16 | 19 |  | |  |  |  |
| bta-miR-449a | IL-10 | 18 | 30 | | bta-miR-12030 | CD14 | 17 | 22 |  | |  |  |  |
| bta-miR-449c | IL-10 | 21 | 30 | | bta-miR-12032 | CD14 | 17 | 25 |  | |  |  |  |
| bta-miR-490 | IL-10 | 12 | 15 | | bta-miR-12034 | CD14 | 16 | 36 |  | |  |  |  |
| bta-miR-760-3p | IL-10 | 17 | 24 | | bta-miR-12034 | CD14 | 12 | 14 |  | |  |  |  |
| bta-miR-2294 | IL-10 | 19 | 38 | | bta-miR-12036 | CD14 | 18 | 22 |  | |  |  |  |
| bta-miR-1343-3p | IL-10 | 12 | 13 | | bta-miR-12038 | CD14 | 17 | 22 |  | |  |  |  |
| bta-miR-2328-3p | IL-10 | 18 | 22 | | bta-miR-12054 | CD14 | 14 | 15 |  | |  |  |  |
| bta-miR-2333 | IL-10 | 16 | 20 | | bta-miR-12062 | CD14 | 15 | 17 |  | |  |  |  |
| bta-miR-2343 | IL-10 | 13 | 16 | | bta-miR-12063 | CD14 | 11 | 13 |  | |  |  |  |
| bta-miR-2356 | IL-10 | 14 | 16 | | bta-miR-26a | TLR4 | 16 | 23 |  | |  |  |  |
| bta-miR-2387 | IL-10 | 17 | 40 | | bta-miR-151-5p | TLR4 | 15 | 17 |  | |  |  |  |
| bta-miR-2398 | IL-10 | 13 | 15 | | bta-miR-27a-5p | TLR4 | 15 | 19 |  | |  |  |  |
| bta-miR-2413 | IL-10 | 16 | 20 | | bta-miR-320a | TLR4 | 16 | 20 |  | |  |  |  |
| bta-miR-2422 | IL-10 | 17 | 20 | | bta-miR-145 | TLR4 | 21 | 40 |  | |  |  |  |
| bta-miR-2430 | IL-10 | 21 | 28 | | bta-miR-181a | TLR4 | 11 | 13 |  | |  |  |  |
| bta-miR-2455 | IL-10 | 17 | 24 | | bta-miR-205 | TLR4 | 17 | 23 |  | |  |  |  |
| bta-miR-2459 | IL-10 | 18 | 32 | | bta-miR-30b-3p | TLR4 | 12 | 15 |  | |  |  |  |
| bta-miR-2464-3p | IL-10 | 13 | 17 | | bta-miR-106a | TLR4 | 17 | 23 |  | |  |  |  |
| bta-miR-2465 | IL-10 | 17 | 32 | | bta-miR-106a | TLR4 | 14 | 16 |  | |  |  |  |
| bta-miR-2483-5p | IL-10 | 10 | 12 | | bta-miR-345-3p | TLR4 | 17 | 22 |  | |  |  |  |
| bta-miR-2486-5p | IL-10 | 17 | 19 | | bta-miR-380-5p | TLR4 | 16 | 18 |  | |  |  |  |
| bta-miR-2902 | IL-10 | 16 | 18 | | bta-let-7d | TLR4 | 17 | 18 |  | |  |  |  |
| bta-miR-26c | IL-10 | 10 | 11 | | bta-miR-17-5p | TLR4 | 17 | 23 |  | |  |  |  |
| bta-miR-149-3p | IL-10 | 18 | 34 | | bta-miR-17-5p | TLR4 | 14 | 16 |  | |  |  |  |
| bta-miR-6518 | IL-10 | 11 | 13 | | bta-miR-200c | TLR4 | 10 | 11 |  | |  |  |  |
| bta-miR-3956 | IL-10 | 18 | 23 | | bta-miR-425-3p | TLR4 | 17 | 21 |  | |  |  |  |
| bta-miR-154b | IL-10 | 11 | 13 | | bta-miR-24-3p | TLR4 | 14 | 17 |  | |  |  |  |
| bta-miR-7862 | IL-10 | 17 | 22 | | bta-miR-24-3p | TLR4 | 14 | 19 |  | |  |  |  |
| bta-miR-378d | IL-10 | 15 | 19 | | bta-let-7a-5p | TLR4 | 17 | 18 |  | |  |  |  |
| bta-miR-10163-3p | IL-10 | 16 | 18 | | bta-miR-487b | TLR4 | 15 | 17 |  | |  |  |  |
| bta-miR-12017 | IL-10 | 12 | 13 | | bta-miR-532 | TLR4 | 16 | 18 |  | |  |  |  |
| bta-miR-12026 | IL-10 | 13 | 15 | | bta-miR-532 | TLR4 | 18 | 22 |  | |  |  |  |
| bta-miR-12030 | IL-10 | 15 | 19 | | bta-miR-23b-5p | TLR4 | 13 | 15 |  | |  |  |  |
| bta-miR-12054 | IL-10 | 15 | 26 | | bta-let-7c | TLR4 | 17 | 18 |  | |  |  |  |
| bta-miR-103 | TLR2 | 14 | 17 | | bta-let-7e | TLR4 | 14 | 16 |  | |  |  |  |
| bta-miR-320a | TLR2 | 15 | 20 | | bta-let-7e | TLR4 | 15 | 17 |  | |  |  |  |
| bta-miR-15b | TLR2 | 16 | 18 | | bta-miR-365-5p | TLR4 | 20 | 44 |  | |  |  |  |
| bta-miR-423-5p | TLR2 | 19 | 23 | | bta-miR-660 | TLR4 | 16 | 19 |  | |  |  |  |
| bta-miR-204 | TLR2 | 13 | 14 | | bta-miR-129 | TLR4 | 19 | 46 |  | |  |  |  |
| bta-miR-497 | TLR2 | 16 | 18 | | bta-miR-129 | TLR4 | 15 | 17 |  | |  |  |  |
| bta-miR-188 | TLR2 | 20 | 28 | | bta-miR-129-5p | TLR4 | 19 | 46 |  | |  |  |  |
| bta-miR-197 | TLR2 | 15 | 18 | | bta-miR-129-5p | TLR4 | 15 | 17 |  | |  |  |  |
| bta-miR-211 | TLR2 | 17 | 22 | | bta-miR-196a | TLR4 | 22 | 55 |  | |  |  |  |
| bta-miR-219-3p | TLR2 | 13 | 15 | | bta-miR-196b | TLR4 | 22 | 55 |  | |  |  |  |
| bta-miR-296-5p | TLR2 | 14 | 20 | | bta-miR-217 | TLR4 | 16 | 21 |  | |  |  |  |
| bta-miR-338 | TLR2 | 11 | 12 | | bta-miR-28 | TLR4 | 18 | 21 |  | |  |  |  |
| bta-miR-541 | TLR2 | 19 | 21 | | bta-miR-324 | TLR4 | 15 | 19 |  | |  |  |  |
| bta-miR-760-5p | TLR2 | 13 | 18 | | bta-miR-329b | TLR4 | 20 | 26 |  | |  |  |  |
| bta-miR-2290 | TLR2 | 14 | 17 | | bta-miR-362-5p | TLR4 | 18 | 20 |  | |  |  |  |
| bta-miR-2316 | TLR2 | 22 | 31 | | bta-miR-378 | TLR4 | 21 | 32 |  | |  |  |  |
| bta-miR-2355-3p | TLR2 | 19 | 24 | | bta-miR-409a | TLR4 | 10 | 13 |  | |  |  |  |
| bta-miR-2360 | TLR2 | 15 | 17 | | bta-miR-432 | TLR4 | 18 | 21 |  | |  |  |  |
| bta-miR-2375 | TLR2 | 20 | 24 | | bta-miR-433 | TLR4 | 18 | 21 |  | |  |  |  |
| bta-miR-2377 | TLR2 | 16 | 18 | | bta-miR-433 | TLR4 | 15 | 20 |  | |  |  |  |
| bta-miR-2381 | TLR2 | 17 | 22 | | bta-miR-491 | TLR4 | 17 | 22 |  | |  |  |  |
| bta-miR-2388-3p | TLR2 | 15 | 23 | | bta-miR-493 | TLR4 | 18 | 21 |  | |  |  |  |
| bta-miR-2423 | TLR2 | 16 | 18 | | bta-miR-551a | TLR4 | 20 | 28 |  | |  |  |  |
| bta-miR-2433 | TLR2 | 21 | 36 | | bta-miR-584 | TLR4 | 16 | 20 |  | |  |  |  |
| bta-miR-2439-5p | TLR2 | 14 | 20 | | bta-miR-615 | TLR4 | 18 | 27 |  | |  |  |  |
| bta-miR-2447 | TLR2 | 18 | 24 | | bta-miR-671 | TLR4 | 20 | 34 |  | |  |  |  |
| bta-miR-2454-5p | TLR2 | 21 | 55 | | bta-miR-760-5p | TLR4 | 18 | 25 |  | |  |  |  |
| bta-miR-2454-5p | TLR2 | 20 | 25 | | bta-miR-760-3p | TLR4 | 17 | 18 |  | |  |  |  |
| bta-miR-339b | TLR2 | 17 | 32 | | bta-miR-760-3p | TLR4 | 18 | 34 |  | |  |  |  |
| bta-miR-1777b | TLR2 | 20 | 23 | | bta-miR-763 | TLR4 | 18 | 29 |  | |  |  |  |
| bta-miR-2461-3p | TLR2 | 11 | 12 | | bta-miR-873 | TLR4 | 18 | 24 |  | |  |  |  |
| bta-miR-2467-3p | TLR2 | 20 | 31 | | bta-miR-877 | TLR4 | 13 | 16 |  | |  |  |  |
| bta-miR-1388-3p | TLR2 | 16 | 21 | | bta-miR-940 | TLR4 | 16 | 20 |  | |  |  |  |
| bta-miR-2897 | TLR2 | 18 | 20 | | bta-miR-1284 | TLR4 | 19 | 51 |  | |  |  |  |
| bta-miR-6121-5p | TLR2 | 17 | 19 | | bta-miR-1197 | TLR4 | 16 | 21 |  | |  |  |  |
| bta-miR-1247-3p | TLR2 | 16 | 21 | | bta-miR-1249 | TLR4 | 16 | 21 |  | |  |  |  |
| bta-miR-6535 | TLR2 | 19 | 22 | | bta-miR-2295 | TLR4 | 17 | 20 |  | |  |  |  |
| bta-miR-10163-3p | TLR2 | 14 | 17 | | bta-miR-2295 | TLR4 | 18 | 22 |  | |  |  |  |
| bta-miR-10170-5p | TLR2 | 16 | 21 | | bta-miR-2296 | TLR4 | 18 | 24 |  | |  |  |  |
| bta-miR-10171-3p | TLR2 | 18 | 28 | | bta-miR-2305 | TLR4 | 16 | 21 |  | |  |  |  |
| bta-miR-10175-3p | TLR2 | 21 | 28 | | bta-miR-2309 | TLR4 | 16 | 20 |  | |  |  |  |
| bta-miR-2285bb | TLR2 | 13 | 15 | | bta-miR-2309 | TLR4 | 18 | 23 |  | |  |  |  |
| bta-miR-11988 | TLR2 | 19 | 36 | | bta-miR-2321 | TLR4 | 15 | 17 |  | |  |  |  |
| bta-miR-11986b | TLR2 | 20 | 24 | | bta-miR-2321 | TLR4 | 16 | 18 |  | |  |  |  |
| bta-miR-11996 | TLR2 | 13 | 15 | | bta-miR-2328-5p | TLR4 | 15 | 20 |  | |  |  |  |
| bta-miR-12037 | TLR2 | 16 | 20 | | bta-miR-2328-3p | TLR4 | 17 | 22 |  | |  |  |  |
| bta-miR-2285dj | TLR2 | 19 | 23 | | bta-miR-2332 | TLR4 | 16 | 17 |  | |  |  |  |
| bta-miR-12049 | TLR2 | 17 | 28 | | bta-miR-2334 | TLR4 | 16 | 21 |  | |  |  |  |
| bta-miR-12055 | TLR2 | 15 | 20 | | bta-miR-2343 | TLR4 | 14 | 17 |  | |  |  |  |
| bta-miR-12057 | TLR2 | 17 | 22 | | bta-miR-2348 | TLR4 | 17 | 20 |  | |  |  |  |
| bta-miR-23a | CCL2 | 12 | 17 | | bta-miR-2356 | TLR4 | 19 | 32 |  | |  |  |  |
| bta-miR-23b-3p | CCL2 | 12 | 17 | | bta-miR-2359 | TLR4 | 14 | 17 |  | |  |  |  |
| bta-miR-183 | CCL2 | 11 | 12 | | bta-miR-2365 | TLR4 | 15 | 18 |  | |  |  |  |
| bta-miR-299 | CCL2 | 12 | 14 | | bta-miR-2366 | TLR4 | 16 | 18 |  | |  |  |  |
| bta-miR-449a | CCL2 | 13 | 20 | | bta-miR-2379 | TLR4 | 13 | 15 |  | |  |  |  |
| bta-miR-449b | CCL2 | 13 | 20 | | bta-miR-2382-5p | TLR4 | 15 | 18 |  | |  |  |  |
| bta-miR-452 | CCL2 | 14 | 16 | | bta-miR-2383 | TLR4 | 17 | 21 |  | |  |  |  |
| bta-miR-376d | CCL2 | 12 | 14 | | bta-miR-2385-5p | TLR4 | 18 | 22 |  | |  |  |  |
| bta-miR-376a | CCL2 | 12 | 14 | | bta-miR-2388-3p | TLR4 | 16 | 22 |  | |  |  |  |
| bta-miR-2295 | CCL2 | 15 | 19 | | bta-miR-2392 | TLR4 | 19 | 39 |  | |  |  |  |
| bta-miR-2309 | CCL2 | 18 | 24 | | bta-miR-2392 | TLR4 | 17 | 22 |  | |  |  |  |
| bta-miR-2349 | CCL2 | 16 | 21 | | bta-miR-2396 | TLR4 | 17 | 22 |  | |  |  |  |
| bta-miR-2356 | CCL2 | 20 | 43 | | bta-miR-2397-3p | TLR4 | 16 | 19 |  | |  |  |  |
| bta-miR-2360 | CCL2 | 17 | 24 | | bta-miR-2398 | TLR4 | 13 | 16 |  | |  |  |  |
| bta-miR-2374 | CCL2 | 18 | 23 | | bta-miR-2413 | TLR4 | 17 | 22 |  | |  |  |  |
| bta-miR-2389 | CCL2 | 17 | 25 | | bta-miR-2415-5p | TLR4 | 20 | 25 |  | |  |  |  |
| bta-miR-2407 | CCL2 | 18 | 21 | | bta-miR-2418 | TLR4 | 18 | 25 |  | |  |  |  |
| bta-miR-2412 | CCL2 | 17 | 22 | | bta-miR-320b | TLR4 | 17 | 20 |  | |  |  |  |
| bta-miR-1777a | CCL2 | 18 | 24 | | bta-miR-2425-5p | TLR4 | 17 | 24 |  | |  |  |  |
| bta-miR-2456 | CCL2 | 13 | 16 | | bta-miR-2425-5p | TLR4 | 13 | 15 |  | |  |  |  |
| bta-miR-1777b | CCL2 | 19 | 25 | | bta-miR-677 | TLR4 | 19 | 22 |  | |  |  |  |
| bta-miR-2467-3p | CCL2 | 19 | 22 | | bta-miR-2436-5p | TLR4 | 21 | 31 |  | |  |  |  |
| bta-miR-2471-5p | CCL2 | 19 | 26 | | bta-miR-2438 | TLR4 | 18 | 23 |  | |  |  |  |
| bta-miR-2474 | CCL2 | 15 | 20 | | bta-miR-2442 | TLR4 | 21 | 40 |  | |  |  |  |
| bta-miR-2888 | CCL2 | 16 | 20 | | bta-miR-2447 | TLR4 | 20 | 38 |  | |  |  |  |
| bta-miR-2892 | CCL2 | 16 | 23 | | bta-miR-2448-3p | TLR4 | 17 | 20 |  | |  |  |  |
| bta-miR-2893 | CCL2 | 17 | 22 | | bta-miR-2450b | TLR4 | 18 | 21 |  | |  |  |  |
| bta-miR-376e | CCL2 | 11 | 12 | | bta-miR-1777a | TLR4 | 19 | 49 |  | |  |  |  |
| bta-miR-1247-3p | CCL2 | 12 | 14 | | bta-miR-2456 | TLR4 | 18 | 25 |  | |  |  |  |
| bta-miR-6525 | CCL2 | 17 | 20 | | bta-miR-2457 | TLR4 | 18 | 29 |  | |  |  |  |
| bta-miR-6528 | CCL2 | 18 | 25 | | bta-miR-1777b | TLR4 | 17 | 27 |  | |  |  |  |
| bta-miR-6528 | CCL2 | 16 | 33 | | bta-miR-2461-3p | TLR4 | 18 | 22 |  | |  |  |  |
| bta-miR-6530 | CCL2 | 12 | 14 | | bta-miR-2473 | TLR4 | 15 | 33 |  | |  |  |  |
| bta-miR-6535 | CCL2 | 18 | 20 | | bta-miR-2474 | TLR4 | 15 | 19 |  | |  |  |  |
| bta-miR-7857-5p | CCL2 | 12 | 13 | | bta-miR-2474 | TLR4 | 14 | 22 |  | |  |  |  |
| bta-miR-4449 | CCL2 | 17 | 23 | | bta-miR-2485 | TLR4 | 15 | 19 |  | |  |  |  |
| bta-miR-10167-3p | CCL2 | 16 | 19 | | bta-miR-2486-5p | TLR4 | 22 | 45 |  | |  |  |  |
| bta-miR-10174-3p | CCL2 | 12 | 16 | | bta-miR-1388-5p | TLR4 | 14 | 15 |  | |  |  |  |
| bta-miR-10176-5p | CCL2 | 14 | 16 | | bta-miR-1388-3p | TLR4 | 18 | 23 |  | |  |  |  |
| bta-miR-10185-5p | CCL2 | 18 | 25 | | bta-miR-2887 | TLR4 | 18 | 24 |  | |  |  |  |
| bta-miR-12001 | CCL2 | 15 | 20 | | bta-miR-2891 | TLR4 | 16 | 20 |  | |  |  |  |
| bta-miR-12025 | CCL2 | 18 | 33 | | bta-miR-2901 | TLR4 | 11 | 12 |  | |  |  |  |
| bta-miR-12026 | CCL2 | 13 | 18 | | bta-miR-2903 | TLR4 | 10 | 11 |  | |  |  |  |
|  |  |  |  | | bta-miR-2284w | TLR4 | 15 | 17 |  | |  |  |  |
|  |  |  |  | | bta-miR-3600 | TLR4 | 14 | 17 |  | |  |  |  |
|  |  |  |  | | bta-miR-3602 | TLR4 | 19 | 25 |  | |  |  |  |
|  |  |  |  | | bta-miR-149-3p | TLR4 | 18 | 22 |  | |  |  |  |
|  |  |  |  | | bta-miR-3141 | TLR4 | 15 | 17 |  | |  |  |  |
|  |  |  |  | | bta-miR-652 | TLR4 | 13 | 15 |  | |  |  |  |
|  |  |  |  | | bta-miR-3956 | TLR4 | 14 | 16 |  | |  |  |  |
|  |  |  |  | | bta-miR-3957 | TLR4 | 18 | 26 |  | |  |  |  |
|  |  |  |  | | bta-miR-378c | TLR4 | 19 | 31 |  | |  |  |  |
|  |  |  |  | | bta-miR-6523a | TLR4 | 19 | 25 |  | |  |  |  |
|  |  |  |  | | bta-miR-6535 | TLR4 | 19 | 23 |  | |  |  |  |
|  |  |  |  | | bta-miR-6535 | TLR4 | 17 | 22 |  | |  |  |  |
|  |  |  |  | | bta-miR-2285z | TLR4 | 18 | 22 |  | |  |  |  |
|  |  |  |  | | bta-miR-7691 | TLR4 | 9 | 11 |  | |  |  |  |
|  |  |  |  | | bta-miR-7862 | TLR4 | 18 | 19 |  | |  |  |  |
|  |  |  |  | | bta-miR-7862 | TLR4 | 19 | 21 |  | |  |  |  |
|  |  |  |  | | bta-miR-7864 | TLR4 | 16 | 18 |  | |  |  |  |
|  |  |  |  | | bta-miR-8549 | TLR4 | 13 | 15 |  | |  |  |  |
|  |  |  |  | | bta-miR-8549 | TLR4 | 13 | 15 |  | |  |  |  |
|  |  |  |  | | bta-miR-4449 | TLR4 | 19 | 32 |  | |  |  |  |
|  |  |  |  | | bta-miR-2450d | TLR4 | 19 | 23 |  | |  |  |  |
|  |  |  |  | | bta-miR-10161-5p | TLR4 | 20 | 32 |  | |  |  |  |
|  |  |  |  | | bta-miR-10161-5p | TLR4 | 18 | 21 |  | |  |  |  |
|  |  |  |  | | bta-miR-10166-5p | TLR4 | 16 | 19 |  | |  |  |  |
|  |  |  |  | | bta-miR-507-3p | TLR4 | 13 | 15 |  | |  |  |  |
|  |  |  |  | | bta-miR-10170-5p | TLR4 | 18 | 24 |  | |  |  |  |
|  |  |  |  | | bta-miR-10173-5p | TLR4 | 18 | 23 |  | |  |  |  |
|  |  |  |  | | bta-miR-10174-5p | TLR4 | 15 | 22 |  | |  |  |  |
|  |  |  |  | | bta-miR-10177-5p | TLR4 | 15 | 19 |  | |  |  |  |
|  |  |  |  | | bta-miR-10179-5p | TLR4 | 18 | 25 |  | |  |  |  |
|  |  |  |  | | bta-miR-10180-3p | TLR4 | 17 | 21 |  | |  |  |  |
|  |  |  |  | | bta-miR-10183-5p | TLR4 | 17 | 23 |  | |  |  |  |
|  |  |  |  | | bta-miR-2285ak-5p | TLR4 | 16 | 17 |  | |  |  |  |
|  |  |  |  | | bta-miR-2285as | TLR4 | 19 | 27 |  | |  |  |  |
|  |  |  |  | | bta-miR-11988 | TLR4 | 15 | 17 |  | |  |  |  |
|  |  |  |  | | bta-miR-1911 | TLR4 | 18 | 22 |  | |  |  |  |
|  |  |  |  | | bta-miR-11993 | TLR4 | 11 | 12 |  | |  |  |  |
|  |  |  |  | | bta-miR-2285bs | TLR4 | 9 | 10 |  | |  |  |  |
|  |  |  |  | | bta-miR-11998 | TLR4 | 18 | 41 |  | |  |  |  |
|  |  |  |  | | bta-miR-12006 | TLR4 | 12 | 14 |  | |  |  |  |
|  |  |  |  | | bta-miR-299-2 | TLR4 | 14 | 18 |  | |  |  |  |
|  |  |  |  | | bta-miR-12010 | TLR4 | 17 | 23 |  | |  |  |  |
|  |  |  |  | | bta-miR-12011 | TLR4 | 18 | 27 |  | |  |  |  |
|  |  |  |  | | bta-miR-12011 | TLR4 | 11 | 12 |  | |  |  |  |
|  |  |  |  | | bta-miR-12015 | TLR4 | 17 | 24 |  | |  |  |  |
|  |  |  |  | | bta-miR-12019 | TLR4 | 22 | 42 |  | |  |  |  |
|  |  |  |  | | bta-miR-2285de | TLR4 | 18 | 20 |  | |  |  |  |
|  |  |  |  | | bta-miR-12029 | TLR4 | 19 | 44 |  | |  |  |  |
|  |  |  |  | | bta-miR-12032 | TLR4 | 19 | 23 |  | |  |  |  |
|  |  |  |  | | bta-miR-12033 | TLR4 | 13 | 15 |  | |  |  |  |
|  |  |  |  | | bta-miR-12039 | TLR4 | 16 | 20 |  | |  |  |  |
|  |  |  |  | | bta-miR-12047 | TLR4 | 18 | 33 |  | |  |  |  |
|  |  |  |  | | bta-miR-12047 | TLR4 | 16 | 21 |  | |  |  |  |
|  |  |  |  | | bta-miR-12048 | TLR4 | 16 | 21 |  | |  |  |  |
|  |  |  |  | | bta-miR-12050 | TLR4 | 21 | 33 |  | |  |  |  |
|  |  |  |  | | bta-miR-12050 | TLR4 | 21 | 33 |  | |  |  |  |
|  |  |  |  | | bta-miR-12051 | TLR4 | 18 | 23 |  | |  |  |  |
|  |  |  |  | | bta-miR-1949 | TLR4 | 20 | 25 |  | |  |  |  |
|  |  |  |  | | bta-miR-12053 | TLR4 | 18 | 21 |  | |  |  |  |
|  |  |  |  | | bta-miR-12059 | TLR4 | 17 | 26 |  | |  |  |  |
|  |  |  |  | | bta-miR-103 | IL-18 | 13 | 14 |  | |  |  |  |
|  |  |  |  | | bta-miR-485 | IL-18 | 18 | 33 |  | |  |  |  |
|  |  |  |  | | bta-miR-584 | IL-18 | 11 | 13 |  | |  |  |  |
|  |  |  |  | | bta-miR-1284 | IL-18 | 12 | 15 |  | |  |  |  |
|  |  |  |  | | bta-miR-1185 | IL-18 | 17 | 20 |  | |  |  |  |
|  |  |  |  | | bta-miR-2301 | IL-18 | 13 | 14 |  | |  |  |  |
|  |  |  |  | | bta-miR-2308 | IL-18 | 18 | 22 |  | |  |  |  |
|  |  |  |  | | bta-miR-2377 | IL-18 | 12 | 16 |  | |  |  |  |
|  |  |  |  | | bta-miR-2392 | IL-18 | 18 | 22 |  | |  |  |  |
|  |  |  |  | | bta-miR-2404 | IL-18 | 13 | 15 |  | |  |  |  |
|  |  |  |  | | bta-miR-1584-5p | IL-18 | 19 | 49 |  | |  |  |  |
|  |  |  |  | | bta-miR-2412 | IL-18 | 15 | 44 |  | |  |  |  |
|  |  |  |  | | bta-miR-2438 | IL-18 | 15 | 20 |  | |  |  |  |
|  |  |  |  | | bta-miR-2448-3p | IL-18 | 14 | 16 |  | |  |  |  |
|  |  |  |  | | bta-miR-664a | IL-18 | 13 | 15 |  | |  |  |  |
|  |  |  |  | | bta-miR-3432a | IL-18 | 17 | 21 |  | |  |  |  |
|  |  |  |  | | bta-miR-149-3p | IL-18 | 19 | 30 |  | |  |  |  |
|  |  |  |  | | bta-miR-6534 | IL-18 | 15 | 18 |  | |  |  |  |
|  |  |  |  | | bta-miR-4657 | IL-18 | 17 | 23 |  | |  |  |  |
|  |  |  |  | | bta-miR-507-3p | IL-18 | 16 | 20 |  | |  |  |  |
|  |  |  |  | | bta-miR-10174-3p | IL-18 | 14 | 16 |  | |  |  |  |
|  |  |  |  | | bta-miR-7180 | IL-18 | 16 | 17 |  | |  |  |  |
|  |  |  |  | | bta-miR-12005 | IL-18 | 14 | 16 |  | |  |  |  |
|  |  |  |  | | bta-miR-103 | IL-10 | 14 | 20 |  | |  |  |  |
|  |  |  |  | | bta-miR-151-5p | IL-10 | 11 | 15 |  | |  |  |  |
|  |  |  |  | | bta-miR-30b-3p | IL-10 | 12 | 14 |  | |  |  |  |
|  |  |  |  | | bta-miR-107 | IL-10 | 14 | 20 |  | |  |  |  |
|  |  |  |  | | bta-miR-423-5p | IL-10 | 19 | 22 |  | |  |  |  |
|  |  |  |  | | bta-miR-34c | IL-10 | 15 | 18 |  | |  |  |  |
|  |  |  |  | | bta-miR-34a | IL-10 | 21 | 26 |  | |  |  |  |
|  |  |  |  | | bta-miR-497 | IL-10 | 15 | 18 |  | |  |  |  |
|  |  |  |  | | bta-miR-106b | IL-10 | 15 | 17 |  | |  |  |  |
|  |  |  |  | | bta-miR-129 | IL-10 | 16 | 19 |  | |  |  |  |
|  |  |  |  | | bta-miR-129-5p | IL-10 | 16 | 19 |  | |  |  |  |
|  |  |  |  | | bta-miR-181d | IL-10 | 19 | 22 |  | |  |  |  |
|  |  |  |  | | bta-miR-187 | IL-10 | 19 | 22 |  | |  |  |  |
|  |  |  |  | | bta-miR-188 | IL-10 | 20 | 36 |  | |  |  |  |
|  |  |  |  | | bta-miR-28 | IL-10 | 9 | 11 |  | |  |  |  |
|  |  |  |  | | bta-miR-370 | IL-10 | 18 | 26 |  | |  |  |  |
|  |  |  |  | | bta-miR-449c | IL-10 | 19 | 27 |  | |  |  |  |
|  |  |  |  | | bta-miR-485 | IL-10 | 11 | 15 |  | |  |  |  |
|  |  |  |  | | bta-miR-376c | IL-10 | 15 | 17 |  | |  |  |  |
|  |  |  |  | | bta-miR-1185 | IL-10 | 11 | 12 |  | |  |  |  |
|  |  |  |  | | bta-miR-1271 | IL-10 | 18 | 22 |  | |  |  |  |
|  |  |  |  | | bta-miR-2296 | IL-10 | 15 | 19 |  | |  |  |  |
|  |  |  |  | | bta-miR-2313-3p | IL-10 | 14 | 17 |  | |  |  |  |
|  |  |  |  | | bta-miR-2338 | IL-10 | 18 | 25 |  | |  |  |  |
|  |  |  |  | | bta-miR-2354 | IL-10 | 16 | 19 |  | |  |  |  |
|  |  |  |  | | bta-miR-2360 | IL-10 | 20 | 25 |  | |  |  |  |
|  |  |  |  | | bta-miR-2373-5p | IL-10 | 20 | 39 |  | |  |  |  |
|  |  |  |  | | bta-miR-2373-3p | IL-10 | 18 | 21 |  | |  |  |  |
|  |  |  |  | | bta-miR-2379 | IL-10 | 13 | 16 |  | |  |  |  |
|  |  |  |  | | bta-miR-2383 | IL-10 | 17 | 24 |  | |  |  |  |
|  |  |  |  | | bta-miR-2385-5p | IL-10 | 20 | 25 |  | |  |  |  |
|  |  |  |  | | bta-miR-2387 | IL-10 | 15 | 19 |  | |  |  |  |
|  |  |  |  | | bta-miR-2392 | IL-10 | 18 | 23 |  | |  |  |  |
|  |  |  |  | | bta-miR-2400 | IL-10 | 18 | 26 |  | |  |  |  |
|  |  |  |  | | bta-miR-2401 | IL-10 | 15 | 17 |  | |  |  |  |
|  |  |  |  | | bta-miR-2402 | IL-10 | 15 | 18 |  | |  |  |  |
|  |  |  |  | | bta-miR-2410 | IL-10 | 19 | 24 |  | |  |  |  |
|  |  |  |  | | bta-miR-1584-5p | IL-10 | 17 | 20 |  | |  |  |  |
|  |  |  |  | | bta-miR-2411-5p | IL-10 | 21 | 55 |  | |  |  |  |
|  |  |  |  | | bta-miR-2413 | IL-10 | 18 | 41 |  | |  |  |  |
|  |  |  |  | | bta-miR-2415-3p | IL-10 | 11 | 12 |  | |  |  |  |
|  |  |  |  | | bta-miR-2416 | IL-10 | 17 | 23 |  | |  |  |  |
|  |  |  |  | | bta-miR-1843 | IL-10 | 20 | 31 |  | |  |  |  |
|  |  |  |  | | bta-miR-2428 | IL-10 | 18 | 42 |  | |  |  |  |
|  |  |  |  | | bta-miR-2433 | IL-10 | 17 | 21 |  | |  |  |  |
|  |  |  |  | | bta-miR-2436-3p | IL-10 | 14 | 21 |  | |  |  |  |
|  |  |  |  | | bta-miR-2438 | IL-10 | 18 | 21 |  | |  |  |  |
|  |  |  |  | | bta-miR-2439-5p | IL-10 | 19 | 22 |  | |  |  |  |
|  |  |  |  | | bta-miR-2442 | IL-10 | 18 | 37 |  | |  |  |  |
|  |  |  |  | | bta-miR-2450b | IL-10 | 16 | 19 |  | |  |  |  |
|  |  |  |  | | bta-miR-2453 | IL-10 | 19 | 24 |  | |  |  |  |
|  |  |  |  | | bta-miR-2460 | IL-10 | 20 | 38 |  | |  |  |  |
|  |  |  |  | | bta-miR-2460 | IL-10 | 19 | 45 |  | |  |  |  |
|  |  |  |  | | bta-miR-664a | IL-10 | 17 | 23 |  | |  |  |  |
|  |  |  |  | | bta-miR-2885 | IL-10 | 16 | 19 |  | |  |  |  |
|  |  |  |  | | bta-miR-2888 | IL-10 | 16 | 19 |  | |  |  |  |
|  |  |  |  | | bta-miR-2892 | IL-10 | 19 | 29 |  | |  |  |  |
|  |  |  |  | | bta-miR-2899 | IL-10 | 13 | 16 |  | |  |  |  |
|  |  |  |  | | bta-miR-2900 | IL-10 | 17 | 24 |  | |  |  |  |
|  |  |  |  | | bta-miR-2901 | IL-10 | 15 | 18 |  | |  |  |  |
|  |  |  |  | | bta-miR-2902 | IL-10 | 17 | 22 |  | |  |  |  |
|  |  |  |  | | bta-miR-409b | IL-10 | 14 | 17 |  | |  |  |  |
|  |  |  |  | | bta-miR-6121-5p | IL-10 | 14 | 19 |  | |  |  |  |
|  |  |  |  | | bta-miR-6529a | IL-10 | 16 | 19 |  | |  |  |  |
|  |  |  |  | | bta-miR-2450d | IL-10 | 14 | 20 |  | |  |  |  |
|  |  |  |  | | bta-miR-10170-5p | IL-10 | 18 | 23 |  | |  |  |  |
|  |  |  |  | | bta-miR-10171-3p | IL-10 | 18 | 35 |  | |  |  |  |
|  |  |  |  | | bta-miR-10175-5p | IL-10 | 14 | 16 |  | |  |  |  |
|  |  |  |  | | bta-miR-10177-5p | IL-10 | 21 | 38 |  | |  |  |  |
|  |  |  |  | | bta-miR-10181-5p | IL-10 | 16 | 20 |  | |  |  |  |
|  |  |  |  | | bta-miR-10181-5p | IL-10 | 13 | 18 |  | |  |  |  |
|  |  |  |  | | bta-miR-11971 | IL-10 | 18 | 21 |  | |  |  |  |
|  |  |  |  | | bta-miR-11976 | IL-10 | 18 | 21 |  | |  |  |  |
|  |  |  |  | | bta-miR-1911 | IL-10 | 14 | 17 |  | |  |  |  |
|  |  |  |  | | bta-miR-299-2 | IL-10 | 13 | 17 |  | |  |  |  |
|  |  |  |  | | bta-miR-12010 | IL-10 | 19 | 24 |  | |  |  |  |
|  |  |  |  | | bta-miR-12011 | IL-10 | 15 | 20 |  | |  |  |  |
|  |  |  |  | | bta-miR-6501 | IL-10 | 19 | 34 |  | |  |  |  |
|  |  |  |  | | bta-miR-12034 | IL-10 | 17 | 22 |  | |  |  |  |
|  |  |  |  | | bta-miR-12043 | IL-10 | 17 | 19 |  | |  |  |  |
|  |  |  |  | | bta-miR-12051 | IL-10 | 14 | 16 |  | |  |  |  |
|  |  |  |  | | bta-miR-12051 | IL-10 | 17 | 26 |  | |  |  |  |
|  |  |  |  | | bta-miR-12060 | IL-10 | 16 | 20 |  | |  |  |  |
|  |  |  |  | | bta-miR-151-3p | TLR2 | 17 | 20 |  | |  |  |  |
|  |  |  |  | | bta-miR-205 | TLR2 | 21 | 44 |  | |  |  |  |
|  |  |  |  | | bta-miR-30b-3p | TLR2 | 18 | 31 |  | |  |  |  |
|  |  |  |  | | bta-miR-193a-5p | TLR2 | 16 | 21 |  | |  |  |  |
|  |  |  |  | | bta-miR-193a-5p | TLR2 | 18 | 24 |  | |  |  |  |
|  |  |  |  | | bta-miR-345-3p | TLR2 | 16 | 20 |  | |  |  |  |
|  |  |  |  | | bta-miR-92a | TLR2 | 13 | 16 |  | |  |  |  |
|  |  |  |  | | bta-miR-132 | TLR2 | 17 | 22 |  | |  |  |  |
|  |  |  |  | | bta-miR-214 | TLR2 | 17 | 22 |  | |  |  |  |
|  |  |  |  | | bta-miR-23a | TLR2 | 14 | 16 |  | |  |  |  |
|  |  |  |  | | bta-miR-24-3p | TLR2 | 16 | 18 |  | |  |  |  |
|  |  |  |  | | bta-miR-23b-5p | TLR2 | 14 | 16 |  | |  |  |  |
|  |  |  |  | | bta-miR-23b-3p | TLR2 | 14 | 16 |  | |  |  |  |
|  |  |  |  | | bta-miR-25 | TLR2 | 16 | 17 |  | |  |  |  |
|  |  |  |  | | bta-miR-25 | TLR2 | 14 | 17 |  | |  |  |  |
|  |  |  |  | | bta-miR-331-3p | TLR2 | 14 | 19 |  | |  |  |  |
|  |  |  |  | | bta-miR-135b | TLR2 | 18 | 22 |  | |  |  |  |
|  |  |  |  | | bta-miR-183 | TLR2 | 12 | 14 |  | |  |  |  |
|  |  |  |  | | bta-miR-296-3p | TLR2 | 20 | 25 |  | |  |  |  |
|  |  |  |  | | bta-miR-329b | TLR2 | 17 | 20 |  | |  |  |  |
|  |  |  |  | | bta-miR-362-5p | TLR2 | 18 | 21 |  | |  |  |  |
|  |  |  |  | | bta-miR-362-5p | TLR2 | 13 | 19 |  | |  |  |  |
|  |  |  |  | | bta-miR-370 | TLR2 | 15 | 21 |  | |  |  |  |
|  |  |  |  | | bta-miR-378 | TLR2 | 16 | 20 |  | |  |  |  |
|  |  |  |  | | bta-miR-485 | TLR2 | 12 | 17 |  | |  |  |  |
|  |  |  |  | | bta-miR-486 | TLR2 | 18 | 22 |  | |  |  |  |
|  |  |  |  | | bta-miR-490 | TLR2 | 18 | 37 |  | |  |  |  |
|  |  |  |  | | bta-miR-543 | TLR2 | 15 | 19 |  | |  |  |  |
|  |  |  |  | | bta-miR-615 | TLR2 | 16 | 18 |  | |  |  |  |
|  |  |  |  | | bta-miR-671 | TLR2 | 17 | 25 |  | |  |  |  |
|  |  |  |  | | bta-miR-760-3p | TLR2 | 14 | 20 |  | |  |  |  |
|  |  |  |  | | bta-miR-767 | TLR2 | 15 | 16 |  | |  |  |  |
|  |  |  |  | | bta-miR-877 | TLR2 | 18 | 23 |  | |  |  |  |
|  |  |  |  | | bta-miR-1224 | TLR2 | 18 | 22 |  | |  |  |  |
|  |  |  |  | | bta-miR-1301 | TLR2 | 16 | 19 |  | |  |  |  |
|  |  |  |  | | bta-miR-1296 | TLR2 | 12 | 15 |  | |  |  |  |
|  |  |  |  | | bta-miR-1197 | TLR2 | 14 | 16 |  | |  |  |  |
|  |  |  |  | | bta-miR-2284i | TLR2 | 13 | 15 |  | |  |  |  |
|  |  |  |  | | bta-miR-2287 | TLR2 | 16 | 23 |  | |  |  |  |
|  |  |  |  | | bta-miR-2295 | TLR2 | 18 | 22 |  | |  |  |  |
|  |  |  |  | | bta-miR-2295 | TLR2 | 18 | 22 |  | |  |  |  |
|  |  |  |  | | bta-miR-2295 | TLR2 | 17 | 21 |  | |  |  |  |
|  |  |  |  | | bta-miR-2299-3p | TLR2 | 18 | 25 |  | |  |  |  |
|  |  |  |  | | bta-miR-2302 | TLR2 | 19 | 24 |  | |  |  |  |
|  |  |  |  | | bta-miR-2305 | TLR2 | 20 | 21 |  | |  |  |  |
|  |  |  |  | | bta-miR-2308 | TLR2 | 15 | 16 |  | |  |  |  |
|  |  |  |  | | bta-miR-1343-5p | TLR2 | 20 | 33 |  | |  |  |  |
|  |  |  |  | | bta-miR-2324 | TLR2 | 19 | 36 |  | |  |  |  |
|  |  |  |  | | bta-miR-2328-3p | TLR2 | 15 | 17 |  | |  |  |  |
|  |  |  |  | | bta-miR-2328-3p | TLR2 | 14 | 16 |  | |  |  |  |
|  |  |  |  | | bta-miR-2330-5p | TLR2 | 20 | 29 |  | |  |  |  |
|  |  |  |  | | bta-miR-2331-5p | TLR2 | 15 | 21 |  | |  |  |  |
|  |  |  |  | | bta-miR-2332 | TLR2 | 20 | 22 |  | |  |  |  |
|  |  |  |  | | bta-miR-2342 | TLR2 | 16 | 20 |  | |  |  |  |
|  |  |  |  | | bta-miR-2343 | TLR2 | 14 | 19 |  | |  |  |  |
|  |  |  |  | | bta-miR-2349 | TLR2 | 19 | 22 |  | |  |  |  |
|  |  |  |  | | bta-miR-2354 | TLR2 | 14 | 18 |  | |  |  |  |
|  |  |  |  | | bta-miR-2284k | TLR2 | 15 | 18 |  | |  |  |  |
|  |  |  |  | | bta-miR-2367-3p | TLR2 | 14 | 20 |  | |  |  |  |
|  |  |  |  | | bta-miR-2368-5p | TLR2 | 16 | 21 |  | |  |  |  |
|  |  |  |  | | bta-miR-2368-5p | TLR2 | 15 | 19 |  | |  |  |  |
|  |  |  |  | | bta-miR-2373-5p | TLR2 | 18 | 26 |  | |  |  |  |
|  |  |  |  | | bta-miR-2379 | TLR2 | 11 | 14 |  | |  |  |  |
|  |  |  |  | | bta-miR-2382-5p | TLR2 | 20 | 25 |  | |  |  |  |
|  |  |  |  | | bta-miR-2389 | TLR2 | 18 | 23 |  | |  |  |  |
|  |  |  |  | | bta-miR-2392 | TLR2 | 17 | 22 |  | |  |  |  |
|  |  |  |  | | bta-miR-2395 | TLR2 | 11 | 16 |  | |  |  |  |
|  |  |  |  | | bta-miR-2397-3p | TLR2 | 13 | 15 |  | |  |  |  |
|  |  |  |  | | bta-miR-2401 | TLR2 | 15 | 18 |  | |  |  |  |
|  |  |  |  | | bta-miR-2409 | TLR2 | 12 | 14 |  | |  |  |  |
|  |  |  |  | | bta-miR-2410 | TLR2 | 14 | 16 |  | |  |  |  |
|  |  |  |  | | bta-miR-1584-5p | TLR2 | 16 | 23 |  | |  |  |  |
|  |  |  |  | | bta-miR-1584-5p | TLR2 | 18 | 20 |  | |  |  |  |
|  |  |  |  | | bta-miR-2411-5p | TLR2 | 19 | 31 |  | |  |  |  |
|  |  |  |  | | bta-miR-2415-5p | TLR2 | 19 | 37 |  | |  |  |  |
|  |  |  |  | | bta-miR-2415-3p | TLR2 | 14 | 19 |  | |  |  |  |
|  |  |  |  | | bta-miR-2416 | TLR2 | 15 | 16 |  | |  |  |  |
|  |  |  |  | | bta-miR-2417 | TLR2 | 13 | 17 |  | |  |  |  |
|  |  |  |  | | bta-miR-2417 | TLR2 | 10 | 11 |  | |  |  |  |
|  |  |  |  | | bta-miR-2422 | TLR2 | 19 | 22 |  | |  |  |  |
|  |  |  |  | | bta-miR-1843 | TLR2 | 20 | 23 |  | |  |  |  |
|  |  |  |  | | bta-miR-2425-5p | TLR2 | 15 | 17 |  | |  |  |  |
|  |  |  |  | | bta-miR-2426 | TLR2 | 14 | 17 |  | |  |  |  |
|  |  |  |  | | bta-miR-2436-5p | TLR2 | 16 | 21 |  | |  |  |  |
|  |  |  |  | | bta-miR-2439-3p | TLR2 | 17 | 24 |  | |  |  |  |
|  |  |  |  | | bta-miR-2441 | TLR2 | 14 | 19 |  | |  |  |  |
|  |  |  |  | | bta-miR-2441 | TLR2 | 14 | 18 |  | |  |  |  |
|  |  |  |  | | bta-miR-2442 | TLR2 | 18 | 38 |  | |  |  |  |
|  |  |  |  | | bta-miR-2449 | TLR2 | 18 | 20 |  | |  |  |  |
|  |  |  |  | | bta-miR-2451 | TLR2 | 17 | 23 |  | |  |  |  |
|  |  |  |  | | bta-miR-2454-5p | TLR2 | 19 | 30 |  | |  |  |  |
|  |  |  |  | | bta-miR-2463 | TLR2 | 15 | 17 |  | |  |  |  |
|  |  |  |  | | bta-miR-2464-3p | TLR2 | 16 | 18 |  | |  |  |  |
|  |  |  |  | | bta-miR-2467-3p | TLR2 | 19 | 23 |  | |  |  |  |
|  |  |  |  | | bta-miR-2469 | TLR2 | 14 | 17 |  | |  |  |  |
|  |  |  |  | | bta-miR-2469 | TLR2 | 16 | 18 |  | |  |  |  |
|  |  |  |  | | bta-miR-2471-5p | TLR2 | 14 | 16 |  | |  |  |  |
|  |  |  |  | | bta-miR-2471-3p | TLR2 | 17 | 27 |  | |  |  |  |
|  |  |  |  | | bta-miR-2472 | TLR2 | 12 | 13 |  | |  |  |  |
|  |  |  |  | | bta-miR-2486-5p | TLR2 | 17 | 24 |  | |  |  |  |
|  |  |  |  | | bta-miR-2882 | TLR2 | 11 | 13 |  | |  |  |  |
|  |  |  |  | | bta-miR-2886 | TLR2 | 15 | 16 |  | |  |  |  |
|  |  |  |  | | bta-miR-2900 | TLR2 | 17 | 19 |  | |  |  |  |
|  |  |  |  | | bta-miR-2900 | TLR2 | 16 | 22 |  | |  |  |  |
|  |  |  |  | | bta-miR-2902 | TLR2 | 21 | 30 |  | |  |  |  |
|  |  |  |  | | bta-miR-2904 | TLR2 | 17 | 23 |  | |  |  |  |
|  |  |  |  | | bta-miR-3432a | TLR2 | 17 | 19 |  | |  |  |  |
|  |  |  |  | | bta-miR-3602 | TLR2 | 17 | 19 |  | |  |  |  |
|  |  |  |  | | bta-miR-149-5p | TLR2 | 16 | 20 |  | |  |  |  |
|  |  |  |  | | bta-miR-149-3p | TLR2 | 19 | 33 |  | |  |  |  |
|  |  |  |  | | bta-miR-3141 | TLR2 | 16 | 20 |  | |  |  |  |
|  |  |  |  | | bta-miR-3141 | TLR2 | 15 | 23 |  | |  |  |  |
|  |  |  |  | | bta-miR-6123 | TLR2 | 13 | 16 |  | |  |  |  |
|  |  |  |  | | bta-miR-6517 | TLR2 | 22 | 42 |  | |  |  |  |
|  |  |  |  | | bta-miR-3154 | TLR2 | 18 | 23 |  | |  |  |  |
|  |  |  |  | | bta-miR-6521 | TLR2 | 18 | 24 |  | |  |  |  |
|  |  |  |  | | bta-miR-6526 | TLR2 | 15 | 18 |  | |  |  |  |
|  |  |  |  | | bta-miR-6528 | TLR2 | 16 | 19 |  | |  |  |  |
|  |  |  |  | | bta-miR-6535 | TLR2 | 19 | 23 |  | |  |  |  |
|  |  |  |  | | bta-miR-2285q | TLR2 | 11 | 13 |  | |  |  |  |
|  |  |  |  | | bta-miR-7860 | TLR2 | 18 | 29 |  | |  |  |  |
|  |  |  |  | | bta-miR-7861 | TLR2 | 15 | 18 |  | |  |  |  |
|  |  |  |  | | bta-miR-4657 | TLR2 | 15 | 16 |  | |  |  |  |
|  |  |  |  | | bta-miR-4449 | TLR2 | 17 | 22 |  | |  |  |  |
|  |  |  |  | | bta-miR-10162-5p | TLR2 | 21 | 38 |  | |  |  |  |
|  |  |  |  | | bta-miR-10171-3p | TLR2 | 19 | 24 |  | |  |  |  |
|  |  |  |  | | bta-miR-10173-5p | TLR2 | 16 | 21 |  | |  |  |  |
|  |  |  |  | | bta-miR-10178-5p | TLR2 | 16 | 21 |  | |  |  |  |
|  |  |  |  | | bta-miR-668-3p | TLR2 | 14 | 18 |  | |  |  |  |
|  |  |  |  | | bta-miR-10179-5p | TLR2 | 20 | 34 |  | |  |  |  |
|  |  |  |  | | bta-miR-10181-5p | TLR2 | 16 | 19 |  | |  |  |  |
|  |  |  |  | | bta-miR-10182-3p | TLR2 | 16 | 22 |  | |  |  |  |
|  |  |  |  | | bta-miR-10183-5p | TLR2 | 19 | 24 |  | |  |  |  |
|  |  |  |  | | bta-miR-10185-5p | TLR2 | 17 | 21 |  | |  |  |  |
|  |  |  |  | | bta-miR-2285ah-5p | TLR2 | 22 | 55 |  | |  |  |  |
|  |  |  |  | | bta-miR-2285ah-5p | TLR2 | 17 | 24 |  | |  |  |  |
|  |  |  |  | | bta-miR-2285ak-5p | TLR2 | 19 | 20 |  | |  |  |  |
|  |  |  |  | | bta-miR-11972 | TLR2 | 18 | 29 |  | |  |  |  |
|  |  |  |  | | bta-miR-11972 | TLR2 | 17 | 20 |  | |  |  |  |
|  |  |  |  | | bta-miR-11979 | TLR2 | 15 | 20 |  | |  |  |  |
|  |  |  |  | | bta-miR-11980 | TLR2 | 15 | 22 |  | |  |  |  |
|  |  |  |  | | bta-miR-11981 | TLR2 | 20 | 28 |  | |  |  |  |
|  |  |  |  | | bta-miR-11983 | TLR2 | 17 | 22 |  | |  |  |  |
|  |  |  |  | | bta-miR-2285bh | TLR2 | 19 | 20 |  | |  |  |  |
|  |  |  |  | | bta-miR-11988 | TLR2 | 18 | 27 |  | |  |  |  |
|  |  |  |  | | bta-miR-11990 | TLR2 | 15 | 20 |  | |  |  |  |
|  |  |  |  | | bta-miR-11990 | TLR2 | 15 | 17 |  | |  |  |  |
|  |  |  |  | | bta-miR-11993 | TLR2 | 15 | 18 |  | |  |  |  |
|  |  |  |  | | bta-miR-2285by | TLR2 | 13 | 14 |  | |  |  |  |
|  |  |  |  | | bta-miR-2285ca | TLR2 | 14 | 17 |  | |  |  |  |
|  |  |  |  | | bta-miR-11997 | TLR2 | 14 | 26 |  | |  |  |  |
|  |  |  |  | | bta-miR-11997 | TLR2 | 14 | 17 |  | |  |  |  |
|  |  |  |  | | bta-miR-12004 | TLR2 | 15 | 21 |  | |  |  |  |
|  |  |  |  | | bta-miR-12007 | TLR2 | 16 | 22 |  | |  |  |  |
|  |  |  |  | | bta-miR-12010 | TLR2 | 17 | 23 |  | |  |  |  |
|  |  |  |  | | bta-miR-12014 | TLR2 | 17 | 28 |  | |  |  |  |
|  |  |  |  | | bta-miR-2285cw | TLR2 | 14 | 15 |  | |  |  |  |
|  |  |  |  | | bta-miR-2285dc | TLR2 | 14 | 16 |  | |  |  |  |
|  |  |  |  | | bta-miR-12029 | TLR2 | 13 | 15 |  | |  |  |  |
|  |  |  |  | | bta-miR-12031 | TLR2 | 16 | 20 |  | |  |  |  |
|  |  |  |  | | bta-miR-12038 | TLR2 | 20 | 50 |  | |  |  |  |
|  |  |  |  | | bta-miR-12042 | TLR2 | 17 | 21 |  | |  |  |  |
|  |  |  |  | | bta-miR-12047 | TLR2 | 16 | 22 |  | |  |  |  |
|  |  |  |  | | bta-miR-12049 | TLR2 | 16 | 22 |  | |  |  |  |
|  |  |  |  | | bta-miR-12054 | TLR2 | 16 | 21 |  | |  |  |  |
|  |  |  |  | | bta-miR-12056 | TLR2 | 20 | 27 |  | |  |  |  |
|  |  |  |  | | bta-miR-12060 | TLR2 | 16 | 23 |  | |  |  |  |
|  |  |  |  | | bta-miR-12061 | TLR2 | 21 | 26 |  | |  |  |  |
|  |  |  |  | | bta-miR-127 | CCL2 | 15 | 20 |  | |  |  |  |
|  |  |  |  | | bta-miR-345-5p | CCL2 | 16 | 22 |  | |  |  |  |
|  |  |  |  | | bta-let-7d | CCL2 | 12 | 14 |  | |  |  |  |
|  |  |  |  | | bta-miR-214 | CCL2 | 17 | 21 |  | |  |  |  |
|  |  |  |  | | bta-let-7b | CCL2 | 13 | 19 |  | |  |  |  |
|  |  |  |  | | bta-let-7c | CCL2 | 13 | 18 |  | |  |  |  |
|  |  |  |  | | bta-miR-195 | CCL2 | 11 | 12 |  | |  |  |  |
|  |  |  |  | | bta-miR-541 | CCL2 | 16 | 23 |  | |  |  |  |
|  |  |  |  | | bta-miR-761 | CCL2 | 11 | 13 |  | |  |  |  |
|  |  |  |  | | bta-miR-767 | CCL2 | 18 | 22 |  | |  |  |  |
|  |  |  |  | | bta-miR-1284 | CCL2 | 18 | 23 |  | |  |  |  |
|  |  |  |  | | bta-miR-2288 | CCL2 | 14 | 19 |  | |  |  |  |
|  |  |  |  | | bta-miR-2302 | CCL2 | 13 | 14 |  | |  |  |  |
|  |  |  |  | | bta-miR-2305 | CCL2 | 14 | 16 |  | |  |  |  |
|  |  |  |  | | bta-miR-2320-5p | CCL2 | 13 | 17 |  | |  |  |  |
|  |  |  |  | | bta-miR-2324 | CCL2 | 19 | 24 |  | |  |  |  |
|  |  |  |  | | bta-miR-2324 | CCL2 | 19 | 24 |  | |  |  |  |
|  |  |  |  | | bta-miR-2328-3p | CCL2 | 16 | 21 |  | |  |  |  |
|  |  |  |  | | bta-miR-2332 | CCL2 | 15 | 18 |  | |  |  |  |
|  |  |  |  | | bta-miR-2360 | CCL2 | 20 | 27 |  | |  |  |  |
|  |  |  |  | | bta-miR-2379 | CCL2 | 10 | 13 |  | |  |  |  |
|  |  |  |  | | bta-miR-2388-3p | CCL2 | 13 | 18 |  | |  |  |  |
|  |  |  |  | | bta-miR-2405 | CCL2 | 18 | 20 |  | |  |  |  |
|  |  |  |  | | bta-miR-2410 | CCL2 | 19 | 25 |  | |  |  |  |
|  |  |  |  | | bta-miR-1584-5p | CCL2 | 15 | 20 |  | |  |  |  |
|  |  |  |  | | bta-miR-2438 | CCL2 | 20 | 27 |  | |  |  |  |
|  |  |  |  | | bta-miR-2438 | CCL2 | 18 | 22 |  | |  |  |  |
|  |  |  |  | | bta-miR-2442 | CCL2 | 20 | 30 |  | |  |  |  |
|  |  |  |  | | bta-miR-2451 | CCL2 | 17 | 27 |  | |  |  |  |
|  |  |  |  | | bta-miR-2454-5p | CCL2 | 18 | 34 |  | |  |  |  |
|  |  |  |  | | bta-miR-2460 | CCL2 | 15 | 17 |  | |  |  |  |
|  |  |  |  | | bta-miR-1777b | CCL2 | 20 | 42 |  | |  |  |  |
|  |  |  |  | | bta-miR-2483-3p | CCL2 | 15 | 18 |  | |  |  |  |
|  |  |  |  | | bta-miR-669 | CCL2 | 14 | 16 |  | |  |  |  |
|  |  |  |  | | bta-miR-669 | CCL2 | 12 | 16 |  | |  |  |  |
|  |  |  |  | | bta-miR-2888 | CCL2 | 18 | 23 |  | |  |  |  |
|  |  |  |  | | bta-miR-2902 | CCL2 | 17 | 34 |  | |  |  |  |
|  |  |  |  | | bta-miR-3141 | CCL2 | 14 | 15 |  | |  |  |  |
|  |  |  |  | | bta-miR-6519 | CCL2 | 10 | 12 |  | |  |  |  |
|  |  |  |  | | bta-miR-3956 | CCL2 | 16 | 19 |  | |  |  |  |
|  |  |  |  | | bta-miR-503-5p | CCL2 | 10 | 12 |  | |  |  |  |
|  |  |  |  | | bta-miR-7860 | CCL2 | 13 | 18 |  | |  |  |  |
|  |  |  |  | | bta-miR-10020 | CCL2 | 17 | 22 |  | |  |  |  |
|  |  |  |  | | bta-miR-10167-3p | CCL2 | 16 | 22 |  | |  |  |  |
|  |  |  |  | | bta-miR-11975 | CCL2 | 16 | 20 |  | |  |  |  |
|  |  |  |  | | bta-miR-11976 | CCL2 | 16 | 20 |  | |  |  |  |
|  |  |  |  | | bta-miR-7180 | CCL2 | 11 | 13 |  | |  |  |  |
|  |  |  |  | | bta-miR-6501 | CCL2 | 19 | 25 |  | |  |  |  |
