# Supplementary material for: Regulatory network of miRNA, lncRNA, transcription factor and target immune response genes in bovine mastitis
Source: Sci Rep. 2021 Nov 9;11:21899. doi: 10.1038/s41598-021-01280-9 (PMC8578396; doi:10.1038/s41598-021-01280-9)
Supplement: Supplementary file 14 — Supplementary Table S3. [file 41598_2021_1280_MOESM14_ESM.docx]

**Supplementary Table 3.** List of miRNAs from all three software and their target candidate gene (three or more). ** indicates the miRNA can bind to two different regions and *** indicated the miRNA can bind to three regions.

| **List of miRNAs** | **Candidate Gene(s)** |
| --- | --- |
| **bta-miR-2467-3p | CCL2 CD4 CSF2 CXCL8 MYD88 TLR2 |
| bta-miR-23-3p | CCL2 CXCL8 ICAM1 TLR2 TLR4 TNFα |
| bta-miR-331-3p | CD4 CSF2 MYD88 TLR2 TLR4 TNFα |
| bta-miR-760-3p | CD86 CSF2 CXCL8 ICAM1 IL-10 MYD88 |
| bta-miR-27-3p | CCL2 CD4 CD86 TLR4 TNFα |
| bta-miR-543 | CCL2 CD86 CXCL8 IL-10 TLR2 |
| **bta-miR-7865 | CD14 CD86 CXCL8 ICAM1 IL-6 |
| bta-miR-328 | CD86 CSF2 ICAM1 IFNγ TNFα |
| bta-miR-24-3p | CD86 ICAM1 IFNγ MYD88 TLR4 |
| bta-miR-181 | CD86 IFNγ IL-4 TLR4 TNFα |
| bta-miR-410 | CSF2 CXCL8 IL-10 IL-4 TLR4 |
| bta-miR-302 | CXCL8 IFNγ IL-10 MYD88 TLR4 |
| ***bta-miR-2360 | CCL2 CD14 CD4 MYD88 |
| ***bta-miR-149-5p | CD14 CD86 IL-10 MYD88 |
| **bta-miR-1197 | CCL2 CD86 TLR4 TNFα |
| **bta-miR-326 | CD4 CD86 ICAM1 TLR4 |
| **bta-miR-485 | CD4 CD86 MYD88 TLR4 |
| bta-miR-124 | CCL2 ICAM1 MYD88 TLR4 |
| bta-miR-143 | CD14 CD4 ICAM1 IFNγ |
| bta-miR-381 | CD4 CD86 CXCL8 TLR4 |
| bta-miR-150 | CD4 CD86 IL-10 TNFα |
| bta-miR-873 | CD4 ICAM1 IL-4 TLR4 |
| bta-miR-491 | CD4 ICAM1 MYD88 TLR4 |
| bta-miR-339 | CD86 CSF2 MYD88 TLR4 |
| bta-miR-145 | CD86 CXCL8 TLR4 TNFα |
| bta-miR-342 | CD86 ICAM1 MYD88 TLR4 |
| bta-miR-494 | CD86 ICAM1 TLR4 TNFα |
| bta-miR-199-5p | CSF2 CXCL8 MYD88 TLR4 |
| bta-miR-223 | IFNγ IL-6 TLR4 TNFα |
| bta-miR-15b, bta-miR-16a | IL-10 IL-18 IL-6 TNFα |
| bta-miR-155 | IL-18 IL-6 TLR4 TNFα |
| ***bta-miR-6535 | MYD88 TLR2 TLR4 |
| ***bta-miR-10171-3p | CD86 IL-6 TLR2 |
| ***bta-miR-2413 | IFNγ IL-10 TLR4 |
| **bta-miR-2888 | CCL2 MYD88 TNFα |
| **bta-miR-2394 | CD14 CD4 MYD88 |
| **bta-miR-2285ah-5p | CD4 CXCL8 IL-6 |
| **bta-miR-874 | CD4 ICAM1 TLR4 |
| **bta-miR-431 | CD14 TLR2 TLR4 |
| **bta-miR-138 | CD14 MYD88 TLR4 |
| **bta-miR-486 | CXCL8 MYD88 TLR2 |
| **bta-miR-185 | ICAM1 MYD88 TLR4 |
| bta-miR-7 | CCL2 CD4 CD86 |
| bta-miR-151-3p | CCL2 CD86 TLR4 |
| bta-miR-495 | CCL2 CXCL8 IFNγ |
| bta-miR-369-3p | CCL2 CXCL8 IL-10 |
| bta-miR-142-5p | CCL2 CXCL8 TLR4 |
| bta-miR-136 | CCL2 ICAM1 TLR4 |
| bta-miR-374 | CCL2 IL-10 TLR4 |
| bta-miR-146 | CD4 TLR2 TLR4 |
| bta-miR-455-5p | CD4 MYD88 TLR4 |
| bta-miR-653 | CD86 ICAM1 TLR4 |
| bta-miR-1 | CD86 IL-10 TNFα |
| bta-miR-448, bta-miR-153 | CXCL8 ICAM1 TLR4 |
| bta-miR-98 | CXCL8 IL-10 IL-4 |
| bta-miR-876 | CXCL8 IL-10 TLR4 |
| bta-miR-421 | CXCL8 MYD88 TLR4 |
| bta-miR-125 | ICAM1 IFNγ TNFα |
| bta-miR-365-3p, bta-miR-129-5p | ICAM1 IL-4 TLR4 |
| bta-miR-377 | ICAM1 TLR2 TLR4 |
| bta-miR-21-5p, bta-miR-204 | ICAM1 TLR4 TNFα |
| bta-miR-26 | IFNγ IL-4 TLR4 |
| bta-miR-18 | IFNγ MYD88 TLR4 |
