# Supplementary material for: Regulatory network of miRNA, lncRNA, transcription factor and target immune response genes in bovine mastitis
Source: Sci Rep. 2021 Nov 9;11:21899. doi: 10.1038/s41598-021-01280-9 (PMC8578396; doi:10.1038/s41598-021-01280-9)
Supplement: Supplementary file 15 — Supplementary Table S4. [file 41598_2021_1280_MOESM15_ESM.docx]

**Supplementary Table 4.** List of Bovine lncRNA mined from NONCODE and their target candidate genes, genomic location, strand type, and length. If available, the accession number for NCBI is listed. * Indicates lncRNA was sued for further analysis

| **List of lncRNA** | **Accession Number** | **No.** | **Candidate Genes** | **Genomic Location** | **Strand (+/-)** | **Length (bp)** |
| --- | --- | --- | --- | --- | --- | --- |
| *NONBTAT001181.2 |  | 16 | MYD88, CD4, IFNγ, IL-4, ICAM1, IL-18, CD86, CSF2, CCL2, TLR4, CXCL8, IL-10, IL-6, CD14, TLR2, TNFα | 1: 106662044-106699223 | (+) | 478 |
| NONBTAT001484.2 | XR_001501401.2 | 2 | ICAM1, CD14 | 1:139995398-139996919 | (+) | 746 |
| NONBTAT007285.2 |  | 5 | MYD88, ICAM1, CSF2, IL-10, TLR2 | 15: 63283113-63334591 | (-) | 653 |
| *NONBTAT007847.2 | XR_003029725.1 | 16 | MYD88, CD4, IFNγ, IL-4, ICAM1, IL-18, CD86, CSF2, CCL2, TLR4, CXCL8, IL-10, IL-6, CD14, TLR2, TNFα | 16: 41070664-41155572 | (+) | 287 |
| NONBTAT008468.2 | XR_003029986.1 | 8 | MYD88, CD4, ICAM1, CD86, TLR4, CD14, TLR2, TNFα | 17: 17725760-17779280 | (+) | 612 |
| *NONBTAT010129.2 |  | 16 | MYD88, CD4, IFNγ, IL-4, ICAM1, IL-18, CD86, CSF2, CCL2, TLR4, CXCL8, IL-10, IL-6, CD14, TLR2, TNFα | 19: 36942478-36943050 | (-) | 392 |
| NONBTAT010746.2 |  | 7 | MYD88, CD4, IFNγ, ICAM1, TLR4, IL-10, TNFα | 2: 20376722-20390203 | (+) | 684 |
| *NONBTAT011890.2 | XR_003030515.1 | 16 | MYD88, CD4, IFNγ, IL-4, ICAM1, IL-18, CD86, CSF2, CCL2, TLR4, CXCL8, IL-10, IL-6, CD14, TLR2, TNFα | 2: 123412260-123473251 | (+) | 350 |
| *NONBTAT017501.2 | XR_003033296.1 | 16 | MYD88, CD4, IFNγ, IL-4, ICAM1, IL-18, CD86, CSF2, CCL2, TLR4, CXCL8, IL-10, IL-6, CD14, TLR2, TNFα | 29: 27573349-27582034 | (+) | 268 |
| *NONBTAT021220.2 | XR_234647.4 | 13 | MYD88, CD4, IFNγ, IL-4, ICAM1, IL-18, CSF2, TLR4, CXCL8, IL-10, CD14, TLR2, TNFα | 5: 73503146-73517836 | (-) | 603 |
| NONBTAT021221.2 |  | 12 | MYD88, CD4, IL-4, ICAM1, IL-18, CSF2, CCL2, TLR4, IL-10, CD14, TLR2, TNFα | 5: 73503149-73517830 | (-) | 556 |
| NONBTAT025187.2 |  | 12 | MYD88, CD4, IFNγ, ICAM1, CD86, CSF2, TLR4, CXCL8, IL-10, IL-6, CD14, TNFα | 9: 24344414-24355627 | (+) | 896 |
| NONBTAT026653.2 | XR_816906.3 | 4 | CSF2, CCL2, TLR4, IL-10 | X: 82024473-82028666 | (+) | 785 |
| *NONBTAT027932.1 |  | 16 | MYD88, CD4, IFNγ, IL-4, ICAM1, IL-18, CD86, CSF2, CCL2, TLR4, CXCL8, IL-10, IL-6, CD14, TLR2, TNFα | 13: 66951889-66984441 | (+) | 361 |
| NONBTAT031343.1 |  | 10 | MYD88, CD4, ICAM1, CSF2, TLR4, IL-10, IL-6, CD14, TLR2, TNFα | 8: 42267775-42272300 | (+) | 433 |
| NONBTAT031541.1 | XR_808717.3 | 10 | MYD88, CD4, IL-4, ICAM1, CD86, CSF2, TLR4, CD14, TLR2, TNFα | 9: 36995530-36998857 | (+) | 434 |
| NONBTAT026661.2 | XR_001495594.2 | 5 | MYD88, CD4, CSF2, IL-10, CD14 | X: 82261155-82294467 | (+) | 19275 |
| *NONBTAT013032.2 |  | 13 | MYD88, CD4, ICAM1, IL-18, CD86, CSF2, CCL2, TLR4, CXCL8, IL-10, CD14, TLR2, TNFα | 21: 45339881-45340345 | (-) | 449 |
| NONBTAT021533.2 |  | 7 | CD4, ICAM1, IL-18, CCL2, IL-10, CD14, TNFα | 5: 102201490-102202286 | (+) | 782 |
| NONBTAT027035.2 |  | 11 | MYD88, CD4, IL-4, ICAM1, CD86, CSF2, CCL2, TLR4, CXCL8, IL-10, IL-6 | X: 134309343-134309878 | (+) | 517 |
