# Supplementary material for: Regulatory network of miRNA, lncRNA, transcription factor and target immune response genes in bovine mastitis
Source: Sci Rep. 2021 Nov 9;11:21899. doi: 10.1038/s41598-021-01280-9 (PMC8578396; doi:10.1038/s41598-021-01280-9)
Supplement: Supplementary file 16 — Supplementary Table S5. [file 41598_2021_1280_MOESM16_ESM.docx]

**Supplementary Table 5.** GO Pathway Analysis for candidate bovine mastitis genes.

| **Pathway** | **Gene(s)** | **P-Value** |
| --- | --- | --- |
| Malaria | CXCL8, CCL2, ICAM1, IFNG, IL-10, IL-18, IL-6, MYD88, TLR2, TLR4, TNF | 9.20E-19 |
| African trypanosomiasis | ICAM1, IFNG, IL-10, IL-18, IL-6, MYD88, TNF | 7.00E-11 |
| Legionellosis | CXCL8, CD14, IL-18, IL-6, MYD88, TLR2, TLR4, TNF | 1.00E-11 |
| Rheumatoid arthritis | CXCL8, CD86, CCL2, CSF2, ICAM1, IFNG, IL-18, IL-6, TLR2, TLR4, TNF | 4.60E-16 |
| Inflammatory bowel disease (IBD) | IFNG, IL10, IL-18, IL-4, IL-6, TLR2, TLR4, TNF | 4.60E-11 |
| Allograft rejection | CD86, IFNG, IL-10, IL-4, TNF | 2.70E-06 |
| Leishmaniasis | IFNG, IL-10, IL-4, MYD88, TLR2, TLR4, TNF | 4.10E-09 |
| NOD-like receptor signaling pathway | CXCL8, CCL2, IL-18, IL-6, TNF | 3.20E-06 |
| Salmonella infection | CXCL8, CD14, CSF2, IFNG, IL-18, IL-6, MYD88, TLR4 | 1.60E-10 |
| Graft-versus-host disease | CD86, IFNG, IL-6, TNF | 8.50E-05 |
