# Supplementary material for: Regulatory network of miRNA, lncRNA, transcription factor and target immune response genes in bovine mastitis
Source: Sci Rep. 2021 Nov 9;11:21899. doi: 10.1038/s41598-021-01280-9 (PMC8578396; doi:10.1038/s41598-021-01280-9)
Supplement: Supplementary file 17 — Supplementary Table S6. [file 41598_2021_1280_MOESM17_ESM.docx]

**Supplementary Table 6.** All transcription factors identified for each of the target bovine mastitis genes.

| **Target Genes** | **No. of TFs** | **TF(s)** |
| --- | --- | --- |
| IL-10 | 363 | JUN, AC002126, AP2, AP4, AR, ARID3A, ARNT, ARNTL, ARNTL2, ASCL1, ASCL2, ATF2, ATF3, ATF5, BACH2, BARX2, BATF, BATF:JUN, BCL11A, BCL3, BCL6, BCOR, BHLHE40, BHLHE41, BMI1, BRD2, BRD3, BRD4, BRD7, BTAF1, C17orf96, CASP8AP2, CBFB, CBX3, CBX4, CDK7, CDK8, CDK9, CEBPA, CEBPB, CENPA, CHD1, CREB1, CREB3L2, CREBBP, CTBP2, DBP, DEAF1, DMRT1, DMRT2, DMRT3, DUX4, E2F3, E2F4, E2F7, EBF1, EED, EGR1, EGR2, EHF, ELF1, ELF2, ELK1, ELL2, ELSPBP1, ENO1, EOMES, EP300, EPAS1, ERG, ESR1, ESRRA, ETS1, ETS2, ETV1, EWSR1, EZH1, EZH2, FLI1, FOS, FOSL1, FOSL2, FOXA2, FOXA3, FOXG1, FOXH1, FOXJ3, FOXK1, FOXM1, FOXN3, FOXO3, FOXO6, FOXP1, FOXP2, FOXP3, FUBP1, GABPA, GATA1, GATA2, GATA3, GATA4, GFI1B, GLI3, GLYR1, GMEB2, GTF2A1:GTF2A2, GTF2I, GTF3C2, HCFC1, HDAC1, HDAC2, HDAC6, HES1, HESX1, HIC1, HIF1A, HIRA, HIVEP1, HNF4:COUP, HNF4A, HNF4G, HOXA9, HSF1, IKZF1, INSM1, IRF1, IRF2, IRF3, IRF4, IRF5, IRF8, IRX4, ISGF3, JUND, KAT5, KDM1A, KDM5B, KLF1, KLF11, KLF13, KLF15, KLF3, KLF4, KLF5, KLF7, KMT2A, LHX2, LIN9, LMNB1, LMO2, LRF, LYL1, MAF, MAFF, MAFG, MAFK, MAX, MAZ, MBD2, MED1, MEF2A, MEF2C, MEF2D, MEIS1, MITF, MLL, MTA3, MTF1, MXI1, MYB, MYC, MYEF2, MYF6, MYH11, MYOD1, MYOG, MZF1, NANOG, NCOR1, NCOR2, NFAT5, NFATC1, NFE2, NFE4, NFIC, NFKB1, NFYA, NFYB, NFYC, NHLH1, NKX3-1, NOTCH1, NR1H4, NR2C2, NR2F2, NR2F6, NR3C1, NR3C2, NR5A2, NR6A1, NRF1, ORC1, OTX2, P50:P50, PATZ1, PAX3, PAX5, PAX6, PBX1, PBX3, PCGF2, PDX1, PEBP1, PGR, PHF8, PLAGL1, PML, POLR2A, POLR3A, POU2F1, POU5F1, PPAR:HNF4:COUP:RAR, PPARD/PPARG, PPARGC1A, PRAME, PRDM1, PRKDC, PTEN, PURA, RARA, RARB, RARG, RBL2, RBPJ, RCOR1, RELA, REPIN1, REST, RFX2, RFX5, RNF2, RREB1, RUNX1, RUNX1T1, RUNX2, RUNX3, RXR:RAR, RXR:RAR_DR5, RXRA, RXRB, RXRG, SETDB1, SIN3A, SIRT6, SIX5, SMAD1, SMAD2/SMAD3, SMAD2:SMAD3:SMAD4, SMAD3, SMAD4, SMARCA4, SMARCB1, SNAI2, SOAT1, SOX10, SOX17, SOX2, SP1, SP2, SP3, SP4, SP7, SP8, SPDEF, SPI1, SPIB, SRC, SREBF1, SREBF2, SREBP1, SRF, STAT1, STAT2, STAT2:STAT1, STAT3, STAT3:STAT3, STAT4, STAT5A, STAT6, SUMO2, SUZ12, T, TAF1, TAF3, TAL1, TBL1XR1, TBP, TBR1, TBX2, TBX22, TBX3, TBX5, TCF12, TCF21, TCF3, TCF4, TCF7L2, TEAD1, TEAD4, TEF, TFAP2A, TFAP2C, TFAP2E, TFAP4, TFCP2, TFEB, TFEC, THAP1, THAP11, THRA, THRB, TLX1, TP53, TP73, TRIM24, TRIM28, TRIM63, UBTF, USF1, USF2, VDR, VDR:CAR:PXR, VEZF1, WHSC1, WT1, XBP1, YY1, ZBTB10, ZBTB17, ZBTB18, ZBTB33, ZBTB7A, ZBTB7B, ZEB1, ZFX, ZFY, ZKSCAN1, ZNF143, ZNF219, ZNF238, ZNF250, ZNF263, ZNF274, ZNF280D, ZNF35, ZNF350, ZNF384, ZNF711, ZNF740, ZNF75A, ZNF76, ZNF92, ZSCAN16 |
|  |  |  |
| IFNγ | 283 | JUN, AP1, AP4, AR, ARID3A, ARID5B, ARNT, ARRB1, ASCL2, ATF2, ATF3, BACH1, BACH2, BATF, BCL6, BCOR, BDP1, BHLHE40, BRCA1, BRD3, BRD4, BRD7, BRF1, BTAF1, C17orf96, CBFB, CBX3, CBX4, CDK8, CDK9, CEBPA, CEBPB, COL11A2, CREB1, CREBBP, CTBP2, CTCF, DMRT2, DUX4, E2F4, E2F6, E2F7, EBF1, EHF, ELL2, EN1, ENSG00000250096, EP300, EPAS1, ERG, ESR1, ETS1, ETS2, ETV4, EWSR1, EZH2, FLI1, FOS, FOSL1, FOSL2, FOXA1, FOXA2, FOXA3, FOXC2, FOXD3, FOXF1, FOXI1, FOXJ2, FOXJ3, FOXK1, FOXM1, FOXN3, FOXO1, FOXO3, FOXP1, FOXP3, FOXP4, GABPA, GATA1, GATA2, GATA3, GATA4, GFI1B, GLI1, GLI3, GTF2A1:GTF2A2, GTF2B, GTF2I, GTF3C2, HAND2, HDAC1, HDAC6, HEY1, HIC2, HIF1A, HMG20B, HMGA1, HNF1A, HNF1B, HNF4A, HOXA13, HOXA9, HOXB13, HOXC13, HOXD10, HOXD13, HSF1, IKZF1, IRF1, IRF2, IRF3, IRF4, IRF5, IRF7, IRF8, IRF9, JUNB, JUND, KDM1A, KDM5B, KLF15, KLF3, KLF4, KLF5, KMT2B, LEF1, LEF1:TCF1, LHX2, LIN54, LMNB1, LMO2, LTF, LYL1, MAFK, MAX, MAZ, MBD2, MED1, MEF2A, MEF2C, MEIS1, MEIS3, MITF, MTA3, MXI1, MYB, MYBL2, MYC, MYEF2, MYH11, MYOD1, MZF1, NANOG, NCOR1, NCOR2, NF1, NFAT5, NFATC1, NFATC4, NFE2, NFE2L2, NFIB, NFIC, NFIX, NFYB, NKX2-1, NOTCH1, NR0B1, NR1D1, NR2C1, NR2C2, NR2E1, NR2E3, NR2F6, NR3C1, NR3C2, NR5A2, ONECUT2, OTX2, PAX3, PAX5, PAX6, PAX7, PBX3, PCGF2, PGR, PITX2, PITX3, PML, POLR2A, POLR3A, POU2F1, POU3F2, PPARG, PRDM1, PURA, RAC3, RAD21, RB1, RBL2, RCOR1, RELA, REPIN1, REST, RFX2, RNF2, RORA, RREB1, RUNX1, RUNX1T1, RUNX2, RUNX3, RXRA, RXRG, SETDB1, SIN3A, SMAD1, SMAD2, SMAD3, SMAD4, SMARCA4, SOX11, SOX17, SOX2, SOX9, SP1, SP2, SP4, SPI1, SPIB, SRC, SREBF2, SRF, SRY, STAG1, STAT1, STAT3, STAT4, STAT5A, STAT5B, STAT6, SUMO2, SUMO2/SUMO3, T, TAF1, TAL1, TBL1XR1, TBP, TBX10, TCF12, TCF3, TCF4, TCF7L2, TEAD4, TFAP2A, TFAP4, TFDP1, TOP1, TP53, TRIM24, TRIM28, UBN1, USF1, VDR, VEZF1, WT1, ZBTB10, ZBTB17, ZC3H8, ZFHX3, ZFX, ZFY, ZKSCAN1, ZNF143, ZNF202, ZNF250, ZNF263, ZNF274, ZNF280D, ZNF350, ZNF384, ZNF76, ZNF92 |
| IL-4 | 283 | JUN, AP1, AP4, AR, ARID3A, ARID5B, ARNT, ARRB1, ASCL2, ATF2, ATF3, BACH1, BACH2, BATF, BCL6, BCOR, BDP1, BHLHE40, BRCA1, BRD3, BRD4, BRD7, BRF1, BTAF1, C17orf96, CBFB, CBX3, CBX4, CDK8, CDK9, CEBPA, CEBPB, COL11A2, CREB1, CREBBP, CTBP2, CTCF, DMRT2, DUX4, E2F4, E2F6, E2F7, EBF1, EHF, ELL2, EN1, ENSG00000250096, EP300, EPAS1, ERG, ESR1, ETS1, ETS2, ETV4, EWSR1, EZH2, FLI1, FOS, FOSL1, FOSL2, FOXA1, FOXA2, FOXA3, FOXC2, FOXD3, FOXF1, FOXI1, FOXJ2, FOXJ3, FOXK1, FOXM1, FOXN3, FOXO1, FOXO3, FOXP1, FOXP3, FOXP4, GABPA, GATA1, GATA2, GATA3, GATA4, GFI1B, GLI1, GLI3, GTF2A1:GTF2A2, GTF2B, GTF2I, GTF3C2, HAND2, HDAC1, HDAC6, HEY1, HIC2, HIF1A, HMG20B, HMGA1, HNF1A, HNF1B, HNF4A, HOXA13, HOXA9, HOXB13, HOXC13, HOXD10, HOXD13, HSF1, IKZF1, IRF1, IRF2, IRF3, IRF4, IRF5, IRF7, IRF8, IRF9, JUNB, JUND, KDM1A, KDM5B, KLF15, KLF3, KLF4, KLF5, KMT2B, LEF1, LEF1:TCF1, LHX2, LIN54, LMNB1, LMO2, LTF, LYL1, MAFK, MAX, MAZ, MBD2, MED1, MEF2A, MEF2C, MEIS1, MEIS3, MITF, MTA3, MXI1, MYB, MYBL2, MYC, MYEF2, MYH11, MYOD1, MZF1, NANOG, NCOR1, NCOR2, NF1, NFAT5, NFATC1, NFATC4, NFE2, NFE2L2, NFIB, NFIC, NFIX, NFYB, NKX2-1, NOTCH1, NR0B1, NR1D1, NR2C1, NR2C2, NR2E1, NR2E3, NR2F6, NR3C1, NR3C2, NR5A2, ONECUT2, OTX2, PAX3, PAX5, PAX6, PAX7, PBX3, PCGF2, PGR, PITX2, PITX3, PML, POLR2A, POLR3A, POU2F1, POU3F2, PPARG, PRDM1, PURA, RAC3, RAD21, RB1, RBL2, RCOR1, RELA, REPIN1, REST, RFX2, RNF2, RORA, RREB1, RUNX1, RUNX1T1, RUNX2, RUNX3, RXRA, RXRG, SETDB1, SIN3A, SMAD1, SMAD2, SMAD3, SMAD4, SMARCA4, SOX11, SOX17, SOX2, SOX9, SP1, SP2, SP4, SPI1, SPIB, SRC, SREBF2, SRF, SRY, STAG1, STAT1, STAT3, STAT4, STAT5A, STAT5B, STAT6, SUMO2, SUMO2/SUMO3, T, TAF1, TAL1, TBL1XR1, TBP, TBX10, TCF12, TCF3, TCF4, TCF7L2, TEAD4, TFAP2A, TFAP4, TFDP1, TOP1, TP53, TRIM24, TRIM28, UBN1, USF1, VDR, VEZF1, WT1, ZBTB10, ZBTB17, ZC3H8, ZFHX3, ZFX, ZFY, ZKSCAN1, ZNF143, ZNF202, ZNF250, ZNF263, ZNF274, ZNF280D, ZNF350, ZNF384, ZNF76, ZNF92 |
| CXCL8 | 271 | JUN, AP1, AP2, AP4, AR, ASCL2, ATF2, ATF3, BACH2, BARX2, BCL11A, BCL3, BCL6, BCOR, BDP1, BHLHE40, BPTF, BRCA1, BRD2, BRD3, BRD4, BRD7, BRF1, BTAF1, CBFB, CBX1, CBX3, CBX4, CDK8, CDK9, CDX2, CEBPA, CEBPB, CEBPD, CEBPG, CENPA, CHD7, CREB1, CREBBP, CTCF, DMRT1, DMRT2, DMRT3, DMRTA2, DPRX, E2F7, E2F8, EBF1, EGR3, EHF, ELK3, EOMES, EP300, EPAS1, ERG, ESR1, ESR2, ETS1, ETS2, EZH1, EZH2, FERD3L, FEZF1, FLI1, FOS, FOSL2, FOXA1, FOXA2, FOXA3, FOXC2, FOXD3, FOXF2, FOXH1, FOXI1, FOXJ2, FOXJ3, FOXK2, FOXL2, FOXM1, FOXN3, FOXO1, FOXO3, FOXO4, FOXP1, FOXQ1, GATA1, GATA2, GATA3, GATA4, GATA5, GATA6, GFI1B, GTF2I, GTF3C2, HDAC1, HDAC2, HDAC3, HIF1A, HIRA, HIVEP1, HMG20B, HNF4A, HOXA13, HOXA9, HOXB13, HOXD10, HOXD13, HSF1, ICE1, ICE2, IKZF1, IRF1, IRF2, IRF3, IRF4, IRF5, IRF7, IRF8, ISGF3, JUNB, JUND, KDM1A, KDM5B, KLF1, KLF11, KLF4, KMT2B, LHX2, LIN54, LMO2, LYL1, MAF, MAFA, MAX, MAZ, MBD3, MED1, MEF2A, MEF2C, MEIS1, MEOX2, MTA3, MYB, MYBL2, MYC, MYH11, MYOG, NANOG, NCOR2, NF1, NFAT5, NFATC1, NFATC2, NFE4, NFIB, NFIC, NFIX, NFKB1, NFYB, NKX2-1, NKX2-3, NKX3-1, NOTCH1, NPAT, NR2C2, NR2E3, NR2F6, NR3C1, NR5A2, ONECUT1, ONECUT3, OTX2, PALB2, PAX2, PAX3, PAX5, PAX6, PGR, PIAS1, PML, POLR2A, POLR3A, POU1F1, POU2F1, POU3F2, POU3F3, POU5F1, PPARG, PRDM1, PRDM14, PTF1A, RARA, RARG, RCOR1, REL, RELA, REST, REX1, RFX5, RNF2, RREB1, RUNX1, RUNX2, RUNX3, RXRA, RXRG, SETDB1, SMAD1, SMAD2/SMAD3, SMAD3, SMAD4, SMARCA4, SMARCC1, SMARCC2, SOX17, SOX8, SP1, SP2, SPDEF, SPI1, SPIB, SPIC, SRC, SREBF2, SRY, STAT1, STAT2, STAT2:STAT1, STAT3, STAT4, STAT5A, STAT5B, SUMO2, SUMO2/SUMO3, SUZ12, TAF1, TAL1, TBL1XR1, TBP, TBX5, TCF12, TCF21, TCF3, TCF4, TCF7L1, TCF7L2, TEAD1, TEAD4, TEF, TFAP4, TOP1, TOPORS, TP53, TP63, TRIM24, TRIM28, TRIM63, UBN1, USF1, VDR:CAR:PXR, YY1, ZBTB12, ZBTB17, ZKSCAN1, ZNF143, ZNF219, ZNF274, ZNF384, ZNF524, ZNF76, ZNF92 |
| TNFα | 330 | JUN, AR, ARID3A, ASCL1, ATF2, ATF5, BACH1, BACH2, BARX2, BCL11A, BCL3, BCL6, BCOR, BDP1, BHLHE40, BMI1, BRCA1, BRD3, BRD4, BRD7, BRF1, BTAF1, C17orf96, CACD, CBFB, CBX3, CDK8, CDK9, CDX2, CEBPA, CEBPB, CEBPD, CENPA, CHD1, CLOCK, CREB1, CREBBP, CTBP2, CTCF, DDX5, DUX4, E2F1, E2F4, E2F6, E2F7, EBF1, EGR1, EGR2, EGR4, EHF, ELF1, ELF2, ELK1, ELK3, ELK4, ELL2, ENSG00000250096, EOMES, EP300, EPAS1, ERF, ERG, ESR1, ESR2, ESRRA, ESRRB, ETS1, ETS2, ETV1, ETV3, ETV4, EWSR1, EZH1, EZH2, FLI1, FOS, FOXA1, FOXA2, FOXH1, FOXJ2, FOXJ3, FOXK1, FOXM1, FOXN3, FOXO3, FOXP1, FOXP3, FOXP4, GABPA, GATA1, GATA2, GATA3, GATA4, GATA5, GATA6, GLI2, GLI3, GLYR1, GMEB2, GTF2B, GTF2I, GTF3C2, HAND1, HCFC1, HDAC2, HEY2, HIF1A, HMG20B, HOXA10, HOXA6, HOXA9, HSF1, ICE2, IKZF1, IRF1, IRF3, IRF4, IRF5, JMJD6, JUND, KDM1A, KDM5B, KLF1, KLF11, KLF15, KLF16, KLF3, KLF4, KLF5, KLF6, KLF7, KLF9, KMT2A, LMNB1, LMO2, LRF, MAFB, MAX, MAZ, MED1, MED12, MEF2A, MEF2C, MRE11A, MTA3, MTF1, MXI1, MYB, MYBL2, MYC, MYH11, MYOD1, MZF1, NANOG, NCOR1, NCOR2, NF1, NFAT5, NFATC1, NFE2, NFIA, NFIC, NFKB1, NFYA, NFYB, NKX2-1, NOTCH1, NR0B1, NR1D2, NR1H4, NR2C2, NR2F2, NR2F6, NR3C1, NR4A1, NR5A1, NR6A1, NRF1, NRIP1, OTX2, PALB2, PAX2, PAX3, PAX5, PAX6, PBX1, PBX3, PCGF2, PEBP1, PGR, PHF8, PLAG1, PLAGL1, PML, POLR2A, POLR3A, POU2F1, POU3F2, PPARG, PPARG:RXRA, PPARGC1A, PRAME, PRDM1, PRDM4, PRKDC, PURA, RAC3, RAD21, RARA, RARG, RB1, RBCK1, RBL2, RCOR1, REL, RELA, RELB, REPIN1, REST, RFX2, RNF2, RORA, RREB1, RUNX1, RUNX1T1, RUNX2, RUNX3, RXRA, RXRG, SETDB1, SF1, SIN3A, SIRT6, SIX5, SMAD1, SMAD2, SMAD2/SMAD3, SMAD3, SMAD4, SMARCA4, SMARCB1, SMC1A, SMC3, SNAI1, SOX17, SOX2, SP1, SP1:SP3, SP2, SP3, SP4, SP7, SP8, SPDEF, SPI1, SPIB, SRC, SREBF1, SREBF2, SREBP1, SRF, STAG1, STAT1, STAT1:STAT1, STAT2, STAT3, STAT4, STAT5A, STAT5B, STAT6, SUMO2, SUZ12, T, TAF1, TAF3, TAL1, TAL1:TCF3, TBL1XR1, TBP, TBX20, TBX21, TCF12, TCF21, TCF3, TCF4, TEAD4, TFAP2A, TFAP2C, TFAP2E, TFAP4, TFDP1, THAP1, THAP11, TP53, TP63, TP73, TRIM24, TRIM28, TTF2, TWIST1, USF1, USF2, VDR, VEZF1, WT1, YY1, ZBTB10, ZBTB17, ZBTB33, ZBTB4, ZBTB7A, ZBTB7B, ZEB1, ZFHX3, ZFP42, ZKSCAN1, ZNF143, ZNF148, ZNF219, ZNF250, ZNF263, ZNF274, ZNF280D, ZNF281, ZNF384, ZNF589, ZNF740, ZNF76, ZNF92, ZSCAN4 |
| CCL2 | 280 | JUN, AL844527, AP1, AR, ARNT, ASCL1, ATF3, ATF4, ATOH7, BATF, BATF:JUN, BCL6, BDP1, BHLHE40, BRCA1, BRD4, BRF1, BTAF1, C17orf96, CBFB, CBX3, CDK8, CDK9, CEBPA, CHD1, COL11A2, COUP-TF:HNF4, CREB1, CREBBP, CTBP2, CTCF, DDX5, E2F4, E2F7, EHF, ELF1, ELF2, ELF3, ELK1, ELK3, ENSG00000250096, EP300, ERG, ERMAP, ESR1, ESR2, ESRRA, ETS1, ETS2, ETV4, ETV7, EWSR1, EZH2, FEV, FLI1, FOS, FOSL1, FOSL2, FOXA1, FOXA2, FOXD3, FOXI1, FOXJ2, FOXJ3, FOXM1, FOXN3, FOXO1, FOXO3, FOXO6, FOXP1, FOXP4, FUBP1, GABPA, GATA3, GATA4, GATA6, GFI1B, GLI2, GLIS2, GLYR1, GTF2B, GTF2I, GTF3C2, HAND1, HDAC2, HEY1, HIF1A, HIVEP2, HLF, HNF4A, HNF4G, HOXA1, HOXB1, HOXB6, HOXB7, HOXB8, HOXC8, HSF1, ICE2, IKZF1, INSM1, IRF1, IRF2, IRF3, IRF4, IRF5, JMJD6, JUND, KDM1A, KDM5B, KLF1, KLF15, KLF16, KLF4, KLF5, KLF7, KLF9, KMT2B, MAF, MAFG, MAX, MAZ, MBD3, MED1, MEF2A, MEIS1, MRE11A, MTA3, MTF1, MXI1, MYB, MYBL2, MYC, MYEF2, MZF1, NANOG, NCOR1, NCOR2, NF1, NFATC1, NFATC2, NFE2, NFE2L1, NFIL3, NFKB1, NFYB, NKX2-1, NKX6-1, NOTCH1, NR2C2, NR2E3, NR2F1, NR2F2, NR3C1, NR5A2, NRF1, ORC1, OTX2, P50:P50, PATZ1, PAX5, PBX1, PBX3, PCGF2, PDX1, PEBP1, PGR, PKNOX1, PLAG1, PML, POLR2A, POLR3A, POU2F1, POU3F3, POU5F1, PPAR:HNF4:COUP:RAR, PPARA, PPARG, PPARG:RXRA, PPARGC1A, PRDM1, PURA, RAD21, RARA, RB1, RBL2, RBPJ, RCOR1, RELA, REST, RFX2, RFX5, RNF2, RREB1, RUNX1, RUNX2, RXRA, RXRG, SETDB1, SIN3A, SMAD1, SMAD3, SMAD4, SMARCA4, SMARCC1, SMARCC2, SMC1A, SMC3, SNAI1, SOX2, SP1, SP2, SP3, SP4, SP7, SP8, SPI1, SPIB, SRC, SREBF1, SREBF2, SREBP1, SRF, STAG1, STAT1, STAT1:STAT1, STAT2, STAT3, STAT4, STAT5A, STAT5B, SUMO1, SUMO2, SUMO2/SUMO3, SUZ12, TAF1, TAL1, TBP, TBX2, TBX3, TCF12, TCF3, TCF4, TCF7L2, TEAD4, TFAP2A, THAP1, THAP11, TOP1, TOPORS, TP53, TRIM24, TRIM28, USF1, USF2, VDR, VDR:CAR:PXR, VEZF1, WT1, YY1, ZBTB17, ZBTB33, ZBTB4, ZBTB7A, ZBTB7B, ZC3H8, ZEB1, ZFP42, ZKSCAN1, ZNF143, ZNF148, ZNF250, ZNF263, ZNF274, ZNF280D, ZNF281, ZNF32, ZNF384, ZNF92, ZSCAN4 |
| IL-6 | 338 | JUN, AL773544, AP1, AP2, AR, ARID3A, ASCL1, ASCL2, ATF2, ATF3, ATF4, ATOH7, BACH1, BACH2, BARHL1, BCL11A, BCL3, BCL6, BHLHE40, BMI1, BPTF, BRCA1, BRD2, BRD4, BTAF1, C17orf96, CBFB, CBX3, CDK7, CDK8, CDK9, CEBPA, CEBPB, CEBPE, CEBPG, CENPA, CHD7, CIC, CIITA, CLOCK, CREB1, CREBBP, CTBP2, CTCF, CTF1, DDX5, DEAF1, E2F1, E2F4, E2F7, E4F1, EBF1, EED, EGR1, EGR2, EGR4, EHF, ELF1, ELK3, ELL2, ENSG00000250096, EOMES, EP300, EPAS1, ERG, ESR1, ESR2, ETS1, ETS2, ETV1, ETV5, ETV7, EWSR1, EZH1, EZH2, FEV, FLI1, FOS, FOSL1, FOSL2, FOXA1, FOXA2, FOXG1, FOXJ2, FOXK1, FOXM1, FOXO3, FOXP1, FOXP3, FUBP1, GABPA, GATA1, GATA3, GFI1B, GLI1, GLI3, GLYR1, GRHL2, GTF2B, GTF2I, GTF3C2, HDAC1, HDAC2, HDAC6, HIC1, HIF1A, HIVEP1, HIVEP2, HMX2, HNF4A, HOXA5, HOXA6, HOXA9, HOXB1, HOXD3, HSF1, ICE2, IKZF1, INSM1, IRF1, IRF2, IRF3, IRF4, IRF5, JUNB, JUND, KDM1A, KDM5B, KLF1, KLF15, KLF16, KLF4, KLF5, KLF7, KLF9, KMT2A, KMT2B, LEF1, LHX2, LMNB1, LMO2, LXR:PXR:CAR:COUP:RAR, LYL1, MAF, MAFB, MAFF, MAFK, MAX, MAZ, MBD3, MED1, MEF2A, MEIS1, MLL, MNX1, MRE11A, MTF1, MXI1, MYB, MYC, MYEF2, MYH11, MYOD1, MYOG, NANOG, NCOR2, NEUROD1, NEUROG3, NFATC4, NFE2, NFE2:MAF, NFE2L1, NFE2L2, NFIC, NFIX, NFKB1, NFYA, NFYB, NHLH1, NHLH2, NKX2-1, NKX2-2, NKX6-1, NOTCH1, NR1I3, NR2C2, NR2F2, NR3C1, NR5A2, NRF1, NRL, ONECUT1, ORC1, OTX2, P50:P50, P50:RELA-P65, PATZ1, PAX5, PAX6, PBX1, PBX3, PCGF2, PDX1, PGR, PITX2, PKNOX1, PLAG1, PML, POLR2A, POLR3A, POU1F1, POU2F1, POU3F3, POU5F1, POU6F1, PPARD/PPARG, PPARG, PPARG:RXRA, PPARGC1A, PRAME, PRDM1, PRKDC, PURA, RAD21, RARA, RARB, RARG, RB1, RCOR1, REL, RELA, REPIN1, REST, RFX2, RNF2, RREB1, RUNX1, RUNX1T1, RUNX2, RUNX3, RXRA, RXRB, RXRG, SALL4, SATB1, SCRT2, SETDB1, SIN3A, SIX5, SMAD1, SMAD3, SMARCA4, SMARCB1, SMARCC1, SMARCC2, SMC1A, SMC3, SOX17, SOX2, SOX7, SP1, SP1:SP3, SP2, SP3, SP4, SP7, SP8, SPDEF, SPI1, SRC, SREBF1, SREBF2, SRF, SRY, STAG1, STAT1, STAT2, STAT3, STAT5A, STAT5B, SUMO2, SUMO2/SUMO3, SUZ12, T, TAL1, TBL1X, TBL1XR1, TBP, TBX10, TBX21, TBX3, TCF12, TCF21, TCF3, TCF4, TCF7L2, TEAD4, TFAP2A, TFAP2B, TFAP2C, TFAP4, TFF1, THAP11, THRA, TLX1:NFIC, TOP1, TP53, TP73, TRIM24, TRIM28, TWIST1, USF1, USF2, VEZF1, WT1, XBP1, YY1, ZBTB17, ZBTB33, ZBTB7A, ZBTB7B, ZFP42, ZFX, ZIC1, ZIC2, ZKSCAN1, ZNF143, ZNF148, ZNF219, ZNF263, ZNF274, ZNF280D, ZNF281, ZNF384, ZNF410, ZNF589, ZNF76, ZNF92 |
| CSF2 | 338 | JUN, AL773544, AP1, AP2, AR, ARID3A, ASCL1, ASCL2, ATF2, ATF3, ATF4, ATOH7, BACH1, BACH2, BARHL1, BCL11A, BCL3, BCL6, BHLHE40, BMI1, BPTF, BRCA1, BRD2, BRD4, BTAF1, C17orf96, CBFB, CBX3, CDK7, CDK8, CDK9, CEBPA, CEBPB, CEBPE, CEBPG, CENPA, CHD7, CIC, CIITA, CLOCK, CREB1, CREBBP, CTBP2, CTCF, CTF1, DDX5, DEAF1, E2F1, E2F4, E2F7, E4F1, EBF1, EED, EGR1, EGR2, EGR4, EHF, ELF1, ELK3, ELL2, ENSG00000250096, EOMES, EP300, EPAS1, ERG, ESR1, ESR2, ETS1, ETS2, ETV1, ETV5, ETV7, EWSR1, EZH1, EZH2, FEV, FLI1, FOS, FOSL1, FOSL2, FOXA1, FOXA2, FOXG1, FOXJ2, FOXK1, FOXM1, FOXO3, FOXP1, FOXP3, FUBP1, GABPA, GATA1, GATA3, GFI1B, GLI1, GLI3, GLYR1, GRHL2, GTF2B, GTF2I, GTF3C2, HDAC1, HDAC2, HDAC6, HIC1, HIF1A, HIVEP1, HIVEP2, HMX2, HNF4A, HOXA5, HOXA6, HOXA9, HOXB1, HOXD3, HSF1, ICE2, IKZF1, INSM1, IRF1, IRF2, IRF3, IRF4, IRF5, JUNB, JUND, KDM1A, KDM5B, KLF1, KLF15, KLF16, KLF4, KLF5, KLF7, KLF9, KMT2A, KMT2B, LEF1, LHX2, LMNB1, LMO2, LXR:PXR:CAR:COUP:RAR, LYL1, MAF, MAFB, MAFF, MAFK, MAX, MAZ, MBD3, MED1, MEF2A, MEIS1, MLL, MNX1, MRE11A, MTF1, MXI1, MYB, MYC, MYEF2, MYH11, MYOD1, MYOG, NANOG, NCOR2, NEUROD1, NEUROG3, NFATC4, NFE2, NFE2:MAF, NFE2L1, NFE2L2, NFIC, NFIX, NFKB1, NFYA, NFYB, NHLH1, NHLH2, NKX2-1, NKX2-2, NKX6-1, NOTCH1, NR1I3, NR2C2, NR2F2, NR3C1, NR5A2, NRF1, NRL, ONECUT1, ORC1, OTX2, P50:P50, P50:RELA-P65, PATZ1, PAX5, PAX6, PBX1, PBX3, PCGF2, PDX1, PGR, PITX2, PKNOX1, PLAG1, PML, POLR2A, POLR3A, POU1F1, POU2F1, POU3F3, POU5F1, POU6F1, PPARD/PPARG, PPARG, PPARG:RXRA, PPARGC1A, PRAME, PRDM1, PRKDC, PURA, RAD21, RARA, RARB, RARG, RB1, RCOR1, REL, RELA, REPIN1, REST, RFX2, RNF2, RREB1, RUNX1, RUNX1T1, RUNX2, RUNX3, RXRA, RXRB, RXRG, SALL4, SATB1, SCRT2, SETDB1, SIN3A, SIX5, SMAD1, SMAD3, SMARCA4, SMARCB1, SMARCC1, SMARCC2, SMC1A, SMC3, SOX17, SOX2, SOX7, SP1, SP1:SP3, SP2, SP3, SP4, SP7, SP8, SPDEF, SPI1, SRC, SREBF1, SREBF2, SRF, SRY, STAG1, STAT1, STAT2, STAT3, STAT5A, STAT5B, SUMO2, SUMO2/SUMO3, SUZ12, T, TAL1, TBL1X, TBL1XR1, TBP, TBX10, TBX21, TBX3, TCF12, TCF21, TCF3, TCF4, TCF7L2, TEAD4, TFAP2A, TFAP2B, TFAP2C, TFAP4, TFF1, THAP11, THRA, TLX1:NFIC, TOP1, TP53, TP73, TRIM24, TRIM28, TWIST1, USF1, USF2, VEZF1, WT1, XBP1, YY1, ZBTB17, ZBTB33, ZBTB7A, ZBTB7B, ZFP42, ZFX, ZIC1, ZIC2, ZKSCAN1, ZNF143, ZNF148, ZNF219, ZNF263, ZNF274, ZNF280D, ZNF281, ZNF384, ZNF410, ZNF589, ZNF76, ZNF92 |
| CD14 | 314 | JUN, AIRE, AL773544, ALX3, ALX4, AP1, AP4, AR, ARID3A, ARNT2, ARRB1, ARX, ASCL1, ATF3, BACH1, BACH2, BCL11A, BCL3, BCL6, BHLHE40, BMI1, BRCA1, BRD2, BRD3, BRD4, BRD7, BTAF1, C17orf96, CASP8AP2, CBFB, CBX1, CBX3, CDK7, CDK8, CDK9, CDX2, CEBPA, CEBPE, CEBPG, CENPA, CHD1, CHD7, CIC, CREB1, CREBBP, CTBP2, CTCF, DLX1, DRGX, E2F1, E2F4, E2F7, E2F8, EBF1, EED, EGR1, EGR4, EHF, ELF1, ELF2, ELK3, ELL2, EN2, EOMES, EP300, EPAS1, ERG, ESR1, ESR2, ETS1, ETS2, ETV4, EVX2, EWSR1, EZH1, EZH2, FERD3L, FLI1, FOS, FOSL2, FOXA1, FOXA2, FOXD1, FOXG1, FOXM1, FOXO1, FOXP4, GABPA, GATA1, GATA2, GATA4, GBX1, GCM1, GFI1B, GLIS2, GLYR1, GSX1, GTF2I, GTF3C2, HCFC1, HDAC2, HDAC6, HIC1, HIF1A, HIVEP1, HLF, HMX3, HNF4A, HNF4G, HOXA10, HOXA2, HOXA5, HOXB3, HOXB8, HOXB9, HOXD1, HOXD12, HSF, HSF1, IKZF1, IRF1, IRF2, IRF3, IRF4, IRF5, ISL1, JUNB, JUND, KAT5, KAT8, KDM1A, KDM5B, KLF15, KLF4, KLF5, KMT2A, KMT2B, LEF1, LHX2, LHX3, LHX5, LMO2, LTF, LYL1, MAF, MAFA, MAFB, MAFK, MAX, MAZ, MBD3, MED1, MED12, MEF2A, MEF2C, MEIS1, MIXL1, MLXIPL, MYB, MYC, MYH11, MYOD1, MZF1, NANOG, NCOR1, NCOR2, NFAT5, NFATC1, NFE2, NFE2L2, NFE4, NFIL3, NFYA, NFYB, NHLH1, NHLH2, NOTO, NPAT, NR1H4, NR1I3, NR2C2, NR2F2, NR3C1, NRF1, NRL, OGT, ORC1, OTX2, P50:P50, PATZ1, PAX5, PAX6, PBX1, PBX3, PCGF2, PHF8, PHOX2A, PLAG1, PML, POLR2A, POLR3A, POU2F1, POU2F3, POU3F2, POU5F1, PPARA, PPARG, PR, PRDM1, PRKDC, PTF1A, PURA, RAC3, RAD21, RARA, RARB, RARG, RBL2, RBPJ, RCOR1, RELA, REST, RNF2, RUNX1, RXRA, RXRG, SETDB1, SIN3A, SMAD1, SMAD2/SMAD3, SMAD3, SMAD4, SMARCA4, SMARCC1, SMARCC2, SOAT1, SOX1, SOX2, SP1, SP2, SP3, SP4, SP7, SPI1, SPIB, SPIC, SRC, SREBF2, SRF, SRY, STAG1, STAT1, STAT1:STAT1, STAT2, STAT3, STAT4, STAT5A, STAT5B, STAT6, SUMO2/SUMO3, SUZ12, TAF1, TAF3, TAL1, TBL1XR1, TBP, TCF12, TCF21, TCF3, TCF4, TCF7L2, TEAD4, TEF, TFAP2A, TFAP2B, TFAP2C, TFAP2D, TFAP4, TOPORS, TP53, TP73, TRIM24, TRIM28, USF1, USF2, VDR, VEZF1, VSX1, VSX2, WHSC1, WT1, XBP1, YY1, ZBTB10, ZBTB14, ZBTB17, ZBTB33, ZBTB6, ZBTB7A, ZBTB7B, ZFHX2, ZFX, ZKSCAN1, ZNF143, ZNF148, ZNF263, ZNF274, ZNF280D, ZNF281, ZNF35, ZNF384, ZNF740, ZNF92, ZSCAN4 |
|  |  |  |
