# Supplementary material for: Regulatory network of miRNA, lncRNA, transcription factor and target immune response genes in bovine mastitis
Source: Sci Rep. 2021 Nov 9;11:21899. doi: 10.1038/s41598-021-01280-9 (PMC8578396; doi:10.1038/s41598-021-01280-9)
Supplement: Supplementary file 18 — Supplementary Table S7. [file 41598_2021_1280_MOESM18_ESM.docx]

**Supplementary Table 7.** lncRNA-miRNA predicted binding data.

| **lncRNA** | **lncRNA Length** | **miRNA** | **miRNA Length** | **dG** | **ndG** | **Start Position lncRNA** | **End Position lncRNA** | **Start Position miRNA** | **End Position miRNA** |
| --- | --- | --- | --- | --- | --- | --- | --- | --- | --- |
| XR_234647.4 | 603 | bta-miR-328 | 22 | -5.75 | -2.875 | 1 | 10 | 13 | 22 |
| NONBTAT010129.2 | 392 | bta-miR-149-5p | 23 | -4.97 | -2.485 | 79 | 101 | 1 | 23 |
| NONBTAT001181.2 | 478 | bta-miR-185 | 22 | -4 | -2 | 60 | 81 | 1 | 22 |
| XR_234647.4 | 603 | bta-miR-223 | 22 | -7.53 | -1.506 | 514 | 535 | 1 | 22 |
| NONBTAT027932.1 | 361 | bta-miR-185 | 22 | -6.71 | -1.342 | 104 | 125 | 1 | 22 |
| NONBTAT027932.1 | 361 | bta-miR-149-5p | 23 | -6.76 | -1.1267 | 7 | 29 | 1 | 23 |
| XR_003030515.1 | 350 | bta-miR-149-5p | 23 | -7.68 | -0.96 | 1 | 17 | 7 | 23 |
| NONBTAT001181.2 | 478 | bta-miR-328 | 22 | -6.71 | -0.9586 | 248 | 269 | 1 | 22 |
| XR_003029725.1 | 287 | bta-miR-185 | 22 | -6.55 | -0.9357 | 119 | 140 | 1 | 22 |
| NONBTAT001181.2 | 478 | bta-miR-223 | 22 | -4.02 | -0.804 | 248 | 269 | 1 | 22 |
| NONBTAT001181.2 | 478 | bta-miR-874 | 22 | -7.82 | -0.782 | 264 | 285 | 1 | 22 |
| NONBTAT010129.2 | 392 | bta-miR-24-3p | 22 | -5.43 | -0.7757 | 370 | 391 | 1 | 22 |
| XR_003030515.1 | 350 | bta-miR-185 | 22 | -5.43 | -0.7757 | 161 | 182 | 1 | 22 |
| NONBTAT001181.2 | 478 | bta-miR-149-5p | 23 | -4.96 | -0.7086 | 259 | 281 | 1 | 23 |
| XR_003029725.1 | 287 | bta-miR-223 | 22 | -5.53 | -0.6913 | 205 | 226 | 1 | 22 |
| NONBTAT027932.1 | 361 | bta-miR-24-3p | 22 | -6.41 | -0.641 | 303 | 324 | 1 | 22 |
| XR_003029725.1 | 287 | bta-miR-328 | 22 | -6.72 | -0.6109 | 229 | 250 | 1 | 22 |
| XR_003030515.1 | 350 | bta-miR-223 | 22 | -4.25 | -0.6071 | 165 | 186 | 1 | 22 |
| NONBTAT013032.2 | 449 | bta-miR-24-3p | 22 | -5.18 | -0.5756 | 247 | 268 | 1 | 22 |
| XR_003033296.1 | 268 | bta-miR-149-5p | 23 | -5.33 | -0.533 | 239 | 261 | 1 | 23 |
| XR_003033296.1 | 268 | bta-miR-24-3p | 22 | -6.36 | -0.53 | 26 | 47 | 1 | 22 |
| NONBTAT001181.2 | 478 | bta-miR-24-3p | 22 | -6.1 | -0.5083 | 379 | 400 | 1 | 22 |
| XR_234647.4 | 603 | bta-miR-24-3p | 22 | -6.05 | -0.5042 | 441 | 462 | 1 | 22 |
| NONBTAT027932.1 | 361 | bta-miR-874 | 22 | -7.54 | -0.5027 | 333 | 354 | 1 | 22 |
| XR_003030515.1 | 350 | bta-miR-328 | 22 | -6.86 | -0.49 | 14 | 35 | 1 | 22 |
| XR_234647.4 | 603 | bta-miR-185 | 22 | -5.58 | -0.465 | 312 | 333 | 1 | 22 |
| XR_003029725.1 | 287 | bta-miR-874 | 22 | -5.92 | -0.4554 | 1 | 19 | 4 | 22 |
| XR_003033296.1 | 268 | bta-miR-874 | 22 | -6.74 | -0.4493 | 51 | 72 | 1 | 22 |
| NONBTAT010129.2 | 392 | bta-miR-328 | 22 | -6.2 | -0.4429 | 59 | 80 | 1 | 22 |
| NONBTAT010129.2 | 392 | bta-miR-185 | 22 | -7.27 | -0.4276 | 244 | 265 | 1 | 22 |
| NONBTAT027932.1 | 361 | bta-miR-223 | 22 | -4.7 | -0.4273 | 220 | 241 | 1 | 22 |
| NONBTAT027932.1 | 361 | bta-miR-328 | 22 | -7.61 | -0.4228 | 207 | 228 | 1 | 22 |
| NONBTAT013032.2 | 449 | bta-miR-223 | 22 | -4.27 | -0.3558 | 315 | 336 | 1 | 22 |
| XR_003029725.1 | 287 | bta-miR-24-3p | 22 | -4.53 | -0.3485 | 71 | 92 | 1 | 22 |
| NONBTAT013032.2 | 449 | bta-miR-149-5p | 23 | -5.82 | -0.3424 | 150 | 172 | 1 | 23 |
| XR_234647.4 | 603 | bta-miR-149-5p | 23 | -4.45 | -0.3423 | 1 | 17 | 7 | 23 |
| NONBTAT010129.2 | 392 | bta-miR-223 | 22 | -4.09 | -0.3408 | 48 | 69 | 1 | 22 |
| XR_234647.4 | 603 | bta-miR-874 | 22 | -6.1 | -0.3389 | 333 | 354 | 1 | 22 |
| XR_003030515.1 | 350 | bta-miR-24-3p | 22 | -5.07 | -0.338 | 36 | 57 | 1 | 22 |
| NONBTAT013032.2 | 449 | bta-miR-874 | 22 | -5.38 | -0.3362 | 388 | 409 | 1 | 22 |
| NONBTAT010129.2 | 392 | bta-miR-874 | 22 | -6.31 | -0.3321 | 345 | 366 | 1 | 22 |
| XR_003030515.1 | 350 | bta-miR-874 | 22 | -6.62 | -0.331 | 198 | 219 | 1 | 22 |
| XR_003033296.1 | 268 | bta-miR-328 | 22 | -4.18 | -0.3215 | 46 | 67 | 1 | 22 |
| NONBTAT013032.2 | 449 | bta-miR-185 | 22 | -5.82 | -0.3063 | 18 | 39 | 1 | 22 |
| XR_003033296.1 | 268 | bta-miR-185 | 22 | -4.96 | -0.2918 | 105 | 126 | 1 | 22 |
| NONBTAT013032.2 | 449 | bta-miR-328 | 22 | -5.95 | -0.2833 | 380 | 401 | 1 | 22 |
| XR_003029725.1 | 287 | bta-miR-149-5p | 23 | -5.57 | -0.2652 | 130 | 152 | 1 | 23 |
